# Repetition Suppression Reveals Cue-Specific Spatial Representations for Landmarks and Self-Motion Cues in the Human Retrosplenial Cortex

**DOI:** 10.1523/ENEURO.0294-23.2024

**Published:** 2024-04-08

**Authors:** Xiaoli Chen, Ziwei Wei, Thomas Wolbers

**Affiliations:** ^1^Department of Psychology and Behavioral Sciences, Zhejiang University, Hangzhou 310058, P.R. China; ^2^German Center for Neurodegenerative Diseases (DZNE), Magdeburg 39120, Germany; ^3^Department of Neurology, Otto-von-Guericke University Magdeburg, Magdeburg 39106, Germany; ^4^Center for Behavioral Brain Sciences (CBBS), Otto-von-Guericke University, Magdeburg 39106, Germany

**Keywords:** adaptation, entorhinal cortex, landmark, path integration, retrosplenial cortex, spatial navigation

## Abstract

The efficient use of various spatial cues within a setting is crucial for successful navigation. Two fundamental forms of spatial navigation, landmark-based and self-motion-based, engage distinct cognitive mechanisms. The question of whether these modes invoke shared or separate spatial representations in the brain remains unresolved. While nonhuman animal studies have yielded inconsistent results, human investigation is limited. In our previous work (Chen et al., 2019), we introduced a novel spatial navigation paradigm utilizing ultra-high field fMRI to explore neural coding of positional information. We found that different entorhinal subregions in the right hemisphere encode positional information for landmarks and self-motion cues. The present study tested the generalizability of our previous finding with a modified navigation paradigm. Although we did not replicate our previous finding in the entorhinal cortex, we identified adaptation-based allocentric positional codes for both cue types in the retrosplenial cortex (RSC), which were not confounded by the path to the spatial location. Crucially, the multi-voxel patterns of these spatial codes differed between the cue types, suggesting cue-specific positional coding. The parahippocampal cortex exhibited positional coding for self-motion cues, which was not dissociable from path length. Finally, the brain regions involved in successful navigation differed from our previous study, indicating overall distinct neural mechanisms recruited in our two studies. Taken together, the current findings demonstrate cue-specific allocentric positional coding in the human RSC in the same navigation task for the first time and that spatial representations in the brain are contingent on specific experimental conditions.

## Significance Statement

Effective navigation depends on efficient utilization of various spatial cues within an environment. Understanding how neural representations derived from distinct spatial cues relate—whether they are cue-specific or cue-independent—is paramount. The current study employed desktop virtual reality, ultra-high field fMRI, and a novel repetition suppression paradigm that contrasted landmarks and self-motion cues. While not replicating our previous finding of positional coding in the entorhinal cortex under the new experimental conditions, the current study reveals cue-specific allocentric neural representations of spatial locations in the human retrosplenial cortex for the first time. This finding enriches our understanding of how the brain processes diverse sources of spatial information for cognitive map formation.

## Introduction

How do we keep track of our location as we move about in an environment? Positional coding can rely both on environmental landmarks and self-motion cues, which provide discrete and continuous information, respectively. While one's position can be immediately deduced upon seeing a familiar landmark, self-motion cues—involving body-based cues and optic flow—allow for path integration during locomotion. Given the substantial body of evidence that landmark-based navigation and path integration recruit relatively independent cognitive (Etienne et al., 1996; Chen et al., 2017) and neural processes (Knierim et al., 2014; Chen et al., 2019), a crucial inquiry pertains to whether these two modes of navigation invoke common or distinct spatial representations in the brain. Resolving this question would provide significant insights into a fundamental question in spatial navigation—how the brain integrates different sources of spatial information to construct a coherent cognitive map (Tolman, 1948; O’Keefe and Nadel, 1978; Steel et al., 2021).

In nonhuman animals, positional codes have long been observed in the form of hippocampal place and entorhinal grid cells. Importantly, while some studies suggest that hippocampal spatial maps are cue-specific (Geva-Sagiv et al., 2016), others showed inconclusive results (Quirk et al., 1990; Markus et al., 1994; Save et al., 2000). Furthermore, this cue specificity appears to depend on the behavioral relevance of the cues (Radvansky et al., 2021). In contrast, a significant body of evidence has indicated cue-specific spatial coding in entorhinal cortex (EC), where different subregions process different types of spatial information. Grid cells in the medial entorhinal cortex (MEC) have been frequently implicated in path integration (Gil et al., 2018; Ying et al., 2023). Conversely, the lateral entorhinal cortex (LEC) contains cells that exhibit neuronal activity modulated by the animal's distance to local objects (Deshmukh and Knierim, 2011; Wang et al., 2018). In support of these findings, grid-cell–like activity, which is associated with path integration (Stangl et al., 2018; Bierbrauer et al., 2020), has been observed in the human EC and is predominantly localized to the pmEC (Doeller et al., 2010).

Beyond the EC and the hippocampus (HIPP), position-selective firing is also common in the retrosplenial cortex (RSC; Mao et al., 2017; Miller et al., 2021). The rodent RSC integrates different types of idiothetic and allocentric information, including locomotion, head direction, position, and landmark information to support spatial cognition (Stacho and Manahan-Vaughan, 2022). A defining feature of the positional codes in the RSC is that they typically evolve at later stages of learning (Miller et al., 2019). Critically, place-cell–like cell populations in the RSC largely maintain consistent collective firing patterns regardless of environmental illumination (Mao et al., 2017), which provides preliminary evidence for cue-independent spatial representations.

In humans, the question of cue specificity of spatial coding remains relatively unexplored. One notable exception is an fMRI study by Huffman and Ekstrom (2019) who varied body-based self-motion cues across different environments. Their findings provide preliminary evidence for cue-independent spatial representations in a large-scale brain network, including the RSC and the HIPP.

In addition, our recent fMRI study employed a spatial localization task that allowed for a direct comparison between landmark-based navigation and path integration within the same navigation task and the same environment (Chen et al., 2019). We found that the anterior-lateral EC (alEC) and posterior-medial EC (pmEC) in the right hemisphere were sensitive to inter-location distance for landmark-based navigation and optic-flow-based path integration, respectively. These findings provide preliminary evidence for cue-specific spatial coding in the EC, with different subregions encoding different cues.

However, the generalizability of our previous findings remains uncertain, because multiple factors can influence the neural dynamics of spatial coding, including learning stage (Wolbers and Büchel, 2005; Patai et al., 2019; Diersch et al., 2021) and task difficulty (Patai et al., 2019). Furthermore, this study did not detect neural coding of inter-location distance in other brain regions crucial for navigation such as the HIPP and RSC. To address these issues, the current study modified the virtual navigation task used in our previous study, in which participants navigated along a linear track based on either path integration cues or visual landmarks. To preview, while participants were clearly able to learn spatial locations based on either cue type, we observed little evidence of spatial coding in the EC. In contrast, there was strong evidence for cue-specific allocentric spatial representations in the RSC. Nevertheless, the brain regions involved in successful navigation were different from our previous study, suggesting overall distinct neural mechanisms between the two studies.

## Materials and Methods

### Participants

Twenty healthy adult volunteers from the Magdeburg community participated in this experiment (10 male; mean (±SD) age, 25.35 (±3.19) years). All participants were right-handed, had normal or corrected-to-normal vision, and had no neurological diseases. Three additional participants were tested but were excluded from data analysis, either because they dropped out in the middle of the experiment or because the fMRI data were corrupted by technical problems. All participants gave written informed consent prior to the experiment and received monetary compensation after the experiment. The experiment was approved by the Ethics Committee of the University of Magdeburg.

**Figure 1.**
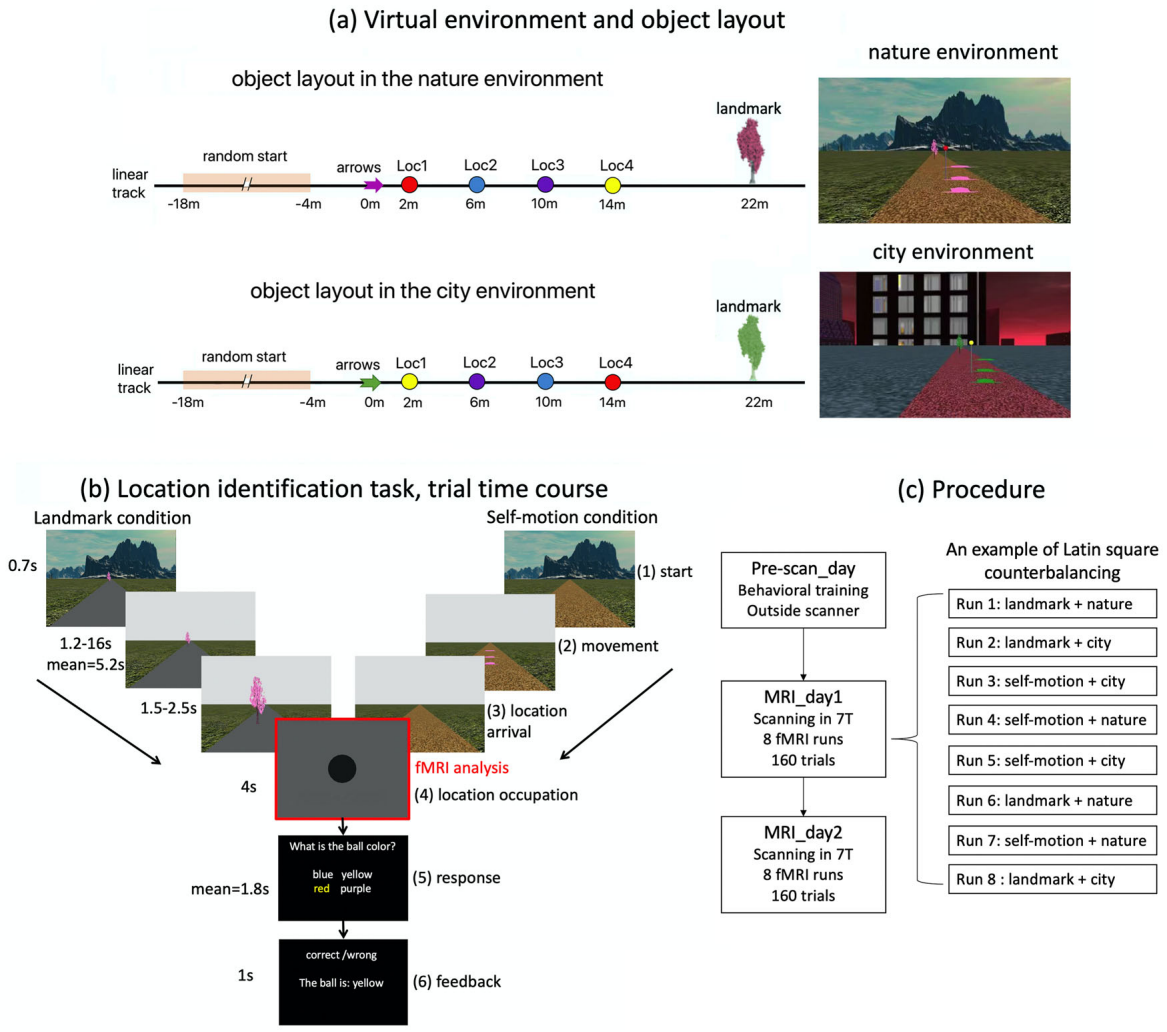
Experimental setup. ***a***, There were two different virtual environments (left): nature (top panel) and city (bottom panel). The two environments shared the same object layout on the linear track (left). There were arrows, four differently colored balls on poles, and a tree on the linear track. The four balls were positioned at the four test locations, that is, Loc1, Loc2, Loc3, and Loc4. To improve visibility, we used three identical arrows positioned above the ground to denote the same spatial position, meaning that the arrows vertically projected to the same position on the ground and only differed in height. The arrows, the tree, and the floor texture of the linear track had the same physical appearances but in different colors in the two environments. The four balls positioned at the test locations were the same but reversed in order in the two environments. The floor texture outside of the linear track also differed between the two environments. Displayed on the right are snapshots of the two environments, with the background environment, the linear track, the tree, the arrows, and the ball positioned closest to the arrows. ***b***, The time course of the location identification task. Here, the trial is depicted in the nature environment. Each trial had six phases. In Phase 1 “start,” the participant was positioned at the starting location, which was randomized trial by trial based on a uniform distribution [−18, −4 m] (Fig. 1*a*, left). In Phase 2 “movement,” the participant was passively transported to one of the four test locations. In Phase 3, after arriving at the test location, the participant's first-person perspective was smoothly turned down to vertically face the ground. In Phase 4 “location occupation,” the participant's perspective was fixed at the ground for four seconds. In Phase 5 “response,” the participant was required to identify the color of the ball positioned at that location within 20 s. In Phase 6 “feedback,” feedback was provided, telling the participant whether the response was accurate and, if incorrect, what the correct answer was. Note that the balls remained invisible throughout the trial, so that participants needed to recall from memory the color of the ball associated with the test location. In the landmark condition, the arrows were invisible, the tree was displayed, and the floor of the linear track remained blank. In the self-motion condition, the arrows were displayed, the tree was invisible, and the texture of the linear track was displayed. In both conditions, the background environment only appeared briefly at the beginning of the trial (=0.7 s) and disappeared once the passive movement started. The fMRI analyses focused on the 4 s location occupation period (i.e., Phase 4), when the visual inputs were the same for both cue conditions. ***c***, Participants were familiarized with the virtual environments and trained in the location identification task on the first day (Pre-scan_day). On the following two days (MRI_day1 and MRI_day2), they completed the location identification task while undergoing MRI scanning in the 7 T scanner. In each scanning session, each of the four condition combinations was conducted for two runs, and the eight runs were counterbalanced with the Latin square design. For full details of the virtual environments and the experimental tasks, see Materials and Methods and the video.

The experiment took place on three separate days. On the first day (referred to as Pre-scan_day), participants received behavioral training. MRI scanning took place on the second day (MRI_day1) and the third day (MRI_day2), as illustrated in Figure 1*c*. The decision to scan participants for two separate days was motivated by the goal of acquiring a substantial number of trials to enhance the statistical power of the study. For the majority of participants, the time interval between Pre-scan_day and MRI_day1 ranged from 1 to 6 d (mean interval, 3.75 d); the time interval between MRI_day1 and MRI_day2 ranged from 1 to 7 d (mean interval, 5.35 d). Due to logistical constraints, three participants experienced a longer time interval of 14 or 17 d. However, given that these participants exhibited no discernible differences in terms of their behavior or fMRI results compared with other participants, they were included in the subsequent analyses.

For the following reasons, we determined our current sample size of 20 participants was appropriate to yield sufficient statistical power. First, our sample size was comparable to a recent fMRI study (Huffman and Ekstrom, 2019) that investigated cue specificity of spatial representations in the human brain. Second, our sample size was closely matched to that of our previous study (Chen et al., 2019), which employed a similar design and analysis methods and involved 22 participants. Importantly, in the current study, we included a considerably larger number of effective trials (i.e., 160 trials per cue type) compared with those in our previous study (Chen et al. 2019; i.e., 40 trials per cue type). Given that statistical power of fMRI experiments depends on both the number of trials and the number of participants (Murphy et al., 2007), the current study possessed a higher empirical statistical power compared to our previous study (Chen et al. 2019). Finally, we estimated the empirical statistical power (based on 20 participants) for replicating the landmark-related neural coding detected in the right alEC in the ROI-based analysis; the calculated statistical power amounted to 0.77, which closely approximates the commonly accepted threshold of 0.8.

### Virtual environment

Two distinct virtual environments were created and rendered in WorldViz 5.0 (https://www.worldviz.com): a city environment and a nature environment (Fig. 1*a*). Both environments shared an identical object layout arranged on a linear track. An arrow set and a tree were positioned at the two ends of the object layout. The arrow set consisted of three identical red arrows positioned at the same position but at different heights from the ground. The tree was positioned with a 0.5 m offset to the left relative to the imaginary midline of the linear track. Between the arrow set and the tree, four balls of different colors were positioned at four predetermined test locations. These test locations were evenly spaced along the linear track, with intervals of 4 m. The surface of the linear track was uniformly textured but rendered in different colors for each environment. Likewise, the arrow set and the tree remained consistent in their appearance but exhibited different colors in the two environments. To further differentiate the two environments, the order of the four balls was reversed while their respective test locations remained constant in the two environments. Additionally, the background view and the ground texture beyond the linear track differed between the two environments.

### Experimental design

The primary experimental task was the “location identification task”, designed to dissociate landmarks and self-motion cues in each trial. To familiarize participants with the positions of the four test locations, they also completed a learning task at times. Details regarding the learning task can be found in Extended Data 1 (Section 1.1; and see all supplementary methods and results in Extended Data 1).

10.1523/ENEURO.0294-23.2024.d1 Extended Data 1 Download Extended Data 1, DOCX file.

10.1523/ENEURO.0294-23.2024.video Video 1 Demo of the experimental environments and tasks, related to Figure 1 and the section 'Stimuli and navigation task' in Methods. Demo of the learning trials starts at 0'0" and ends at 1'24". Demo of the location identification task (i.e., test) starts at 1'25" and ends at 2'48". Download Video 1, MP4 file.

In the “location identification task,” participants were passively transported to one of the four test locations and were required to recall the color of the ball positioned at that location. The ball remained invisible throughout the trial. The temporal sequence of a trial is depicted in Figure 1*b* (also refer to the video, specifically the segment titled “TEST: location identification task”). In each trial, the initial position of the passive movement was randomly selected from a uniform distribution ranging from −18 to −4 m (Fig. 1*a*; the arrow set was positioned at 0 m). Once the passive movement ceased, participants' first-person perspective was fixed at the test location for 4 s, after which they had to report the color of the ball they believed was positioned at their current location. Importantly, the order of the four answer options displayed on the screen was randomized on a trial-by-trial basis, and participants pressed a designated button on the joystick to cycle through the options. This setup ensured that each test location was not associated with a fixed position on the screen or a consistent pattern of joystick movements. To prevent the use of timing or counting strategies, the movement speed during the passive transport was randomly sampled from a uniform distribution ranging from 2 to 5 m/s on a trial-by-trial basis. Accuracy was emphasized, but participants were instructed to not spend longer time than necessary.

To dissociate the use of self-motion cues and landmark cues, we employed an approach similar to our previous study with several adjustments (Chen et al., 2019). This manipulation followed the logic of dissociating landmark and self-motion cues in well-established behavioral paradigms (Nardini et al., 2008; Bates and Wolbers, 2014; Chen et al., 2017). In the self-motion condition, both the arrow set and the linear track texture were visible. As the arrow set served as a reference point for path integration based on traveled distance, participants were able to perform path integration using optic flow after passing the arrow set. However, the landmark was not visible, thereby eliminating landmark-based navigation. To prevent participants from associating the test locations with spatially isolated features on the ground, which would resemble a landmark-based navigation strategy (e.g., the red ball's position was within the brightest patch of the ground), both the texture of the linear track and the floor texture outside the linear track were randomly shifted along the linear track on a trial-by-trial basis, following a uniform distribution ranging from −50 to 50 m.

In contrast, in the landmark condition, the landmark was visible, and it was the only cue participants could use for localization. To eliminate path integration, we removed the arrow set and left the ground texture of the linear track blank to remove texture-based optic flow cues. While there was still peripheral optical flow originating from the floor texture outside of the linear track, participants could not employ path integration effectively. This was due to randomized starting position of the passive movement on a trial-by-trial basis and the absence of the reference point for path integration (i.e., the arrow set). Consequently, participants lacked the necessary information to accurately gauge the traveled distance required to reach a specific ball position. The cue manipulation in the landmark condition aligns with the disorientation manipulation commonly used in spatial navigation studies to eliminate self-motion information (Cheng, 1986; Sutton et al., 2010).

### Experimental procedure

The experiment took place on three separate days: the first day (Pre-scan_day) consisted of behavioral training, while the second day (MRI_day1) and third day (MRI_day2) involved MRI scanning (Fig. 1*c*). The purpose of the behavioral training was twofold: to familiarize participants with the task and to ensure the formation of stable memories of the four test locations before the MRI scanning sessions. For further details regarding the behavioral training on the Pre-scan_day, please refer to Extended Data 1 (Section 1.2).

Both MRI scanning sessions followed the same procedure. At the beginning of each scanning day, participants were refamiliarized with the task through a practice session while undergoing structural MRI scanning inside the scanner. This practice stage lasted ∼5 min and was not analyzed further. Following the practice stage, participants performed the “location identification task” during the subsequent functional scanning (Fig. 1*b*; also refer to the video “TEST: location identification task”).

Each fMRI session consisted of a total of eight runs, with two runs for each of the four combinations of cue condition (self-motion vs landmark) and environment (city vs nature). The eight runs were organized into two blocks, where each block comprised four runs corresponding to the four condition combinations. Within each block, the four condition combinations were semirandomized using Latin square designs and with the restriction that the combinations occurring in two successive runs were different in each fMRI session.

A continuous carry-over design (Aguirre, 2007) was employed to present the trials. We selected eight de Bruijn sequences with high detection power and low correlation coefficient**.** These de Bruijn sequences were generated using a second order counterbalancing approach known as the “path-guided” method (Aguirre et al., 2011). Each de Bruijn sequence consisted of five event types: fixation periods at the four test locations, where participants stayed at the test locations for 4 s, and null events, during which participants fixated their gaze on a centrally displayed cross against a blank screen. Each de Bruijn sequence consisted of 25 events in total, with five repetitions for each event type. To allow the hemodynamic response to reach a steady state before the start of the sequence, the final event in the sequence was duplicated and placed at the beginning. Although this duplicated event was modeled in the first-level GLMs, it was not utilized for the main fMRI analyses. Consequently, each run consisted of 20 effective trials for the main fMRI analyses, with five trials corresponding to each of the four test locations. These eight de Bruijn sequences were randomly assigned to the eight runs in each scanning session for each participant.

During each MRI session, the functional scanning lasted ∼1 h, with the total scanning time lasted up to ∼1.75 h.

### Comparisons with the task in our previous study (Chen et al. 2019)

While the current task shared similarities with the task used in our previous study (Chen et al., 2019) in terms of utilizing a linear track navigation task and dissociating landmarks and self-motion cues, it is important to highlight some key differences. First, participants in our previous study completed the entire experiment within a single day, learning the spatial location of a single target over a brief period (∼15 min) before undergoing functional scanning. In contrast, in the current study, participants received extensive training (∼ 45 min) on a separate day prior to the two MRI scanning sessions. Their behavioral performance had reached a plateau prior to the scanning and remained stable throughout the scanning period (Extended Data 1, Fig. E2), indicating a relatively late spatial memory stage compared with that in our previous study (Chen et al. 2019).

Second, we made the four test locations much easier to discriminate by increasing the spatial distances among them; that is, distance between two adjacent test locations was four virtual meters compared with two virtual meters in the previous study. This modification also resulted in longer temporal intervals between successive location occupation events, as participants needed to traverse longer distances to reach the test locations.

Third, the starting position was randomized on a trial-by-trial basis for both cue conditions in the current study. This allowed for evaluating allocentric positional codes while excluding potential influences of path length for each cue type. In contrast, our previous study implemented this dissociation only in the landmark condition, as the starting position remained fixed for the self-motion condition.

Fourth, unlike our previous study where participants memorized the position of a single target location and judged the relative positions of four equidistant test locations to the target location positioned at the center of the test location layout, the current task required participants to explicitly memorize and recognize the positions of the four equidistant test locations.

Fifth, in our previous study, flashing dots positioned on the floor were used to deliver optic flow information, which are not representative of the optic flow inputs in our daily life. The current study used textured carpets to provide a more realistic optic flow experience.

Sixth, while our previous study employed a single virtual environment, we incorporated two distinct background environments. This adjustment aimed to explore potential effects of the environment on spatial coding, which has been frequently investigated in previous studies (Colgin et al., 2008). Environment served as a secondary factor in our design, while cue type remained the primary focus.

Lastly, while our previous study incorporated both a high-reliability condition and a low-reliability condition for each cue type, in the current study, we did not manipulate cue reliability, and the spatial cues were relatively high in reliability. First, our current focus has shifted away from examining the influence of cue reliability on spatial representations. In addition, our previous study has shown that spatial representations were discernible only when cues reached a sufficient level of reliability. Finally, by excluding the low-reliability conditions, we could maximize the number of trials, thereby enhancing statistical power for detecting and characterizing the neural codes of positional information.

### MRI acquisition

**Figure 2.**
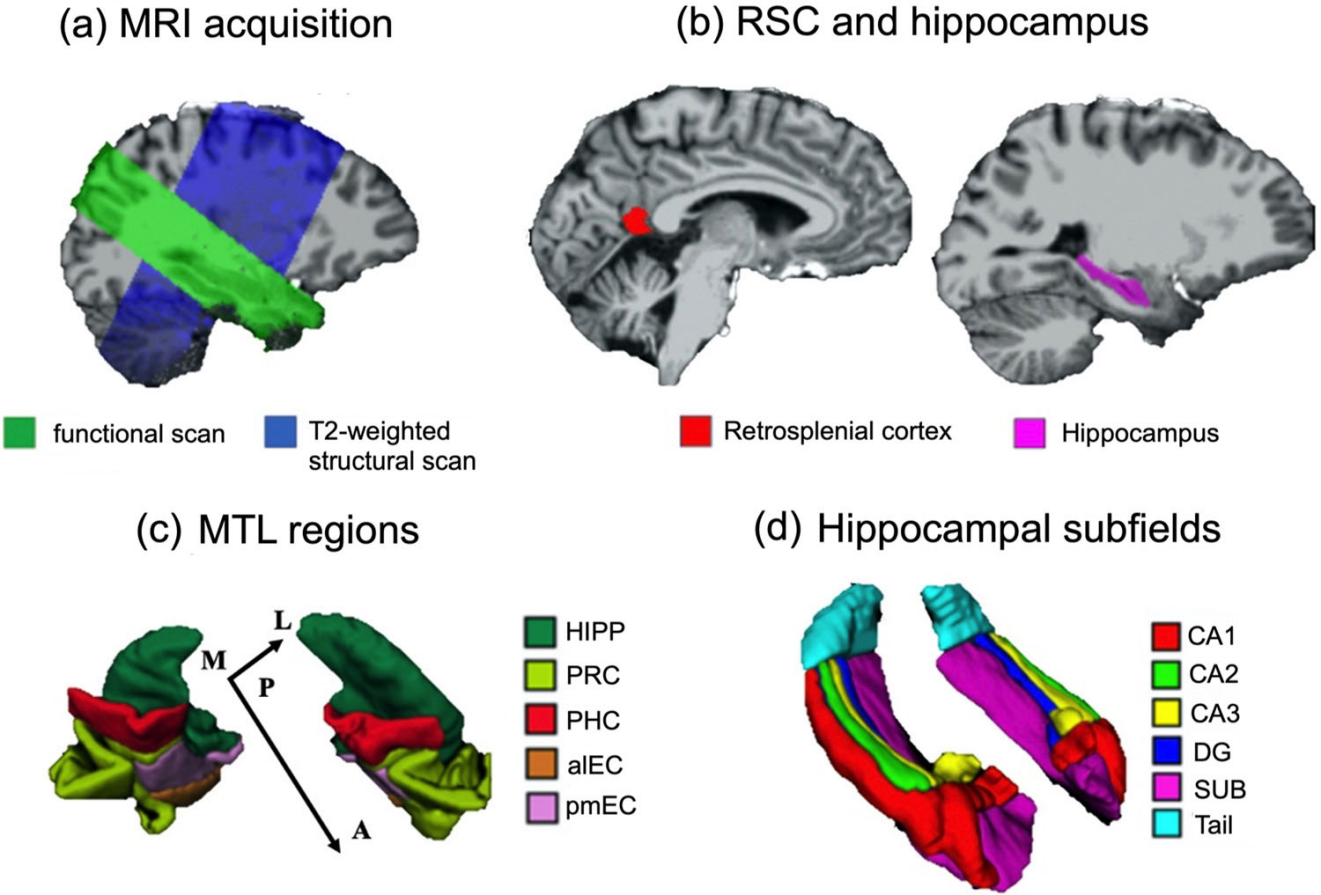
MRI acquisition and anatomical masks of ROIs. ***a***, MRI scanning and ROIs. For an exemplary participant, the functional scan (in green) and the T2-weighted structural scan (in blue) are overlaid on the brain extracted from the T1-weighted structural scan. ***b***, For an exemplary participant, the anatomical mask of the RSC (in red) and the anatomical mask of the HIPP (in violet) are overlaid on the brain extracted from the T1-weighted structural scan. ***c***, Manually segmented anatomical masks for regions in the MTL in one exemplary participant. ***d***, Manually segmented anatomical masks for hippocampal subfields in one exemplary participant. DG, dentate gyrus; SUB, subiculum; PRC, perirhinal cortex; PHC, parahippocampal cortex; alEC, anterior-lateral entorhinal cortex; pmEC, posterior-medial entorhinal cortex. A, anterior; P, posterior; M, medial; L, lateral.

The protocol for MRI acquisition was the same as our previous study (Chen et al., 2019). Structural and functional images were acquired in a 7 T MR scanner (Siemens) at the Leibniz Institute for Neurobiology in Magdeburg with a 32-channel head coil (Nova Medical). A high-resolution whole-brain T1-weighted structural scan was acquired with the following MP-RAGE sequence: TR, 1,700 ms; TE, 2.01 ms; flip angle, 5°; slices, 176; orientation, sagittal; resolution, 1 mm isotropic. A partial-volume turbo spin echo high-resolution T2-weighted structural scan was acquired perpendicular to the long axis of the HIPP (TR, 8,000 ms; TE, 76 ms; flip angle, 60°; slices, 55; slice thickness, 1 mm; distance factor, 10%; in-plane resolution, 0.4 × .0.4 mm; echo spacing, 15.1 ms; turbo factor, 9; echo trains per slice, 57). Functional scans were acquired with a T2*-weighted 2D echo planar image slab centered on the HIPP and parallel to its long axis (TR, 2,000 ms; TE, 22; flip angle, 85°; slices, 35; resolution, 1 mm isotropic; parallel imaging with grappa factor 1; echo spacing, 0.82 ms). We also obtained 10 volumes of whole-brain functional scans for the purpose of coregistering anatomical masks obtained on the T2-weighted structural scan to functional scans with a MPRAGE sequence (TR, 5,000 ms; TE, 22 ms; flip angle, 85°; slices, 100; resolution, 1.6 mm isotropic). The T1-weighted structural image was bias-corrected in SPM12. The functional scans were motion- and distortion-corrected on-line via point spread function mapping (In and Speck, 2012). Figure 2*a* shows the T2-weighted structural scan and a functional scan overlaid on the T1-weighted structural scan for an exemplary participant.

### Anatomical masks for regions of interest

As our regions of interest (ROIs), we focused on the entorhinal subregions, HIPP, RSC, parahippocampal cortex (PHC), and perirhinal cortex (PRC). To illustrate, Figure 2, *b–d*, displays the anatomical masks for an exemplary participant.

As shown in Figure 2*c*, brain regions in the medial temporal lobe (MTL) were manually segmented on each participant's T2-weighted structural scan in ITK-SNAP (Yushkevich et al., 2006; http://www.itksnap.org/pmwiki/pmwiki.php), following an established protocol (Berron et al., 2017). The EC was further divided into the alEC subregion and the pmEC subregion, following the procedure developed in our previous study (Chen et al. 2019). As shown in Figure 2*d*, the HIPP was further segmented into different subfields (CA1, CA2, CA3, subiculum, dentate gyrus, and tail).

RSC mask was automatically extracted from each participant's T1-weighted structural scan (bias-corrected in Advanced Normalization Tools) in FreeSurfer (Dale et al., 1999), using the “recon-all” command. The RSC was defined as the posterior-ventral portion of the cingulate gyrus, which mainly consists of BA29/30. Note that the definition of the RSC is anatomically different from the retrosplenial complex, which is a functionally defined region typically extending into the parieto-occipital sulcus (Epstein, 2008). In addition, although different functional subregions have been identified within the rodent and monkey RSC (Vann et al., 2009), there are currently no established standards for demarcating functional subregions within the RSC in the human brain.

The anatomical mask for the RSC, along with the T1-weighted structural scan, was first coregistered to the mean whole-brain functional scan, which had already been coregistered to the mean functional scan; then the coregistered anatomical mask was resliced using nearest-neighbor interpolation, with the mean functional scan as the reference image. The anatomical masks for the MTL regions were coregistered to the mean functional scan of the first scanning day in SPM12, using the following procedure: first, the mean whole-brain functional scan was coregistered to the mean functional scan; second, the T2-weighted structural scan, along with the anatomical masks, was coregistered to the mean whole-brain functional scan obtained from the first step; and third, the coregistered anatomical masks were resliced using nearest-neighbor interpolation, with the mean functional scan as the reference image.

### Behavioral and fMRI statistical analyses

#### Behavioral data analysis

We calculated behavioral accuracy based on whether the answer was correct (coded as 1) or not (coded as 0), with a chance level of 0.25. For the two scanning days, the first trial of the sequence in each block was not included in the analysis, because it was not included in the main fMRI analyses and did not appear to differ from other trials in the sequence.

#### fMRI univariate adaptation analyses

To investigate spatial coding in the brain, we conducted the location-based fMRI adaptation analysis, based on the logic that the closer the preceding location is to the currently occupied location, the lower the brain activation. To ensure effective comparisons between the current study and our previous study, the fMRI univariate adaptation analyses were conducted in the same way as in our previous study (Chen et al., 2019). In particular, the first-level GLMs were constructed in the same manner as in our previous study.

##### First-level analysis

To assess fMRI adaptation in relation to inter-location distance, we constructed a first-level general linear model (GLM1). Along with the regressors that modeled the location occupation periods (Fig. 1*b*, Phase 4 “location occupation”), we included parametric regressors modeling the modulatory effects of spatial distance between sequentially visited locations. These parametric regressors captured the continuous variation of inter-location distance, specifically with values of 0, 4, 8, and 12 m. Separate regressors were created to model the location occupation periods that were not suitable for adaptation, that is, being preceded by a null event or being in the first trial. To control for potential effects of the passive movement stage on the adaptation effects associated with the ensuing location occupation period, we included regressors modeling the passive movement phase for each run and each cue type, irrespective of the test location. Events were modeled with boxcar regressors convolved with the canonical hemodynamic response function (plus temporal derivative), as implemented in SPM12. Head motion parameters (three rotation parameters and three translation parameters) were included as nuisance regressors, separately for the 16 runs.

To visualize the adaptation effects, we constructed a separate first-level GLM (GLM2), in which separate regressors modeled the location occupation periods with different inter-location distances (i.e., 0, 4, 8, and 12 m). The beta estimates of these regressors were then plotted as a function of inter-location distance.

It is important to note that we did not include separate regressors for all phases in the location identification task. Since natural navigation involves consecutive phases without blank intervals, using separate regressors for each could lead to severe multicollinearity issues, compromising reliability of the parameter estimates. This concern is heightened by our inclusion of the time derivatives for each event regressor, which doubled the number of regressors in the GLM. Therefore, we deliberately minimized the number of regressors, focusing on phases critical for hypothesis testing and with substantial duration to influence neural responses noticeably. This selective approach aimed to balance between capturing essential aspects of the experimental design and maintaining statistical integrity. The detailed rationale follows.

First, Phase 4 “location occupation” was of primary interest; hence, we considered it essential to model this phase explicitly in the GLM. Modeling this phase allowed us to investigate the neural responses associated with specific spatial locations, akin to the investigation of place-sensitive cells, which fire when the mobile agent occupies certain locations in space.

Second, Phase 2 “movement” occupied a substantial portion of the trial and contained sensory information that markedly differed between cue conditions. Consequently, modeling this phase was crucial for accounting for its potential impact on neural responses associated with the location occupation phase.

Third, Phase 1 “start” and Phase 3 “location arrival” were not modeled in the GLM due to their brief durations. The “start” phase averaged 0.7 s in both studies, and the “location arrival” phase on average lasted ∼0.5 s in our previous study and 2 s in the current study. Furthermore, the “location arrival” phase immediately preceded the location occupation phase with no breaks, making it challenging to separate these phases effectively.

Fourth, since Phase 5 “response” and Phase 6 “feedback” followed immediately our phase of interest (Phase 4 “location occupation”), modeling these two phases would introduce multicollinearity issues. In addition, these two phases were the same regardless of cue condition and occupied location, and in the current study we designed the response mode in such way that any location choice was not associated with any consistent pattern of finger movements. Therefore, these two phases should not confound the results associated with the “location occupation” phase.

Nevertheless, to thoroughly evaluate the impact of the preceding navigation experiences on location-based adaptation associated with the location occupation phase, we constructed another GLM, in which the regressors modeling the “movement” phase were extended to include the entire navigation stage (Phase 1 + Phase 2 + Phase 3). Modeling these phases together as a single navigation event considered their continuous nature, avoiding multicollinearity issues. Notably, the main findings on location-based adaptation remained consistent, as the parametric modulation regressors modeling location-based adaptation were minimally correlated with the regressors modeling the extended movement phase in this revised GLM1 (|rs| < 0.011), similar to the original GLM1 (see details in Control analyses, Passive movement phase). This consistency underscores the robustness of our findings across analyses.

##### Second-level ROI-based analysis

First, beta estimates for adaptation of all voxels in each ROI were averaged for each participant. Next, participant-specific beta estimates of adaptation were tested using directional one-sample *t* tests, yielding the uncorrected significance levels. We performed one-sided statistical tests because we hypothesized that BOLD responses should decrease as the distance between successively visited locations decreased, that is, positive adaptation. In addition, negative adaptation is difficult to interpret (Barron et al., 2016). To correct for multiple comparisons, we employed the nonparametric permutation-based maximum *t* statistic approach (Extended Data 1, Section 1.3).

For each group-level *t* test, we calculated the Bayes factor (BF_10_), which indicates the relative likelihood of the alternative hypothesis (i.e., the group mean was >0) over the null hypothesis (i.e., the group mean was not >0; Rouder et al., 2009). The effect size scale (*r*) adopted was 0.707. A BF_10_ greater than 3/10/30 indicates moderate/strong/very-strong evidence for the alternative hypothesis, whereas a BF_10_ less than 0.333/0.1/0.033 indicates moderate/strong/very-strong evidence for the null hypothesis (Jeffreys, 1961). BF_10_ was also calculated in other ROI-based group-level fMRI analyses.

Statistical outliers were identified using the boxplot rule, that is, a value would be considered as an outlier if it is larger than 3rd quartile + 3*interquartile range or smaller than 1st quartile − 3*interquartile range. Statistical outliers were winsorized to the nearest inlier (Reifman and Keyton, 2010).

##### Second-level voxel-wise analysis

Voxel-wise analysis is an important supplement to the ROI analysis. Participant-specific adaptation maps were normalized to the Montreal Neurological Institute (MNI) template and spatially smoothed with 3 mm isotropic FWHM. We conducted directional one-sample *t* tests against 0. To localize adaptation effects within our ROIs, we created group-level anatomical masks for small volume correction (Extended Data 1, Section 1.4). Multiple comparisons were corrected using the nonparametric permutation-based approach (Nichols and Holmes, 2002), using the voxel-level inference approach or the cluster-level inference approach (voxel-wise *T* >3). To explore beyond our ROIs, we corrected for multiple comparisons across the entire volume, also using the nonparametric permutation-based test.

#### fMRI adaptation pattern similarity analysis

The primary goal of this study was to investigate whether or not the neural representations of the four test locations were specific to or independent of the underlying cue type (self-motion vs landmark). Since the voxel-wise adaptation effects served as an index of spatial coding, we investigated cue specificity of this coding with an adaptation pattern similarity analysis. This analysis is analogous to multivariate representational similarity analysis, but with voxel-wise adaptation magnitude instead of voxel-wise activation level as the basic element. If the voxel-to-voxel adaptation pattern differed between landmarks and self-motion cues, this would suggest dissociable neural representations evoked by the two cue types.

The adaptation pattern similarity analysis consisted of several steps. First, an adaptation vector was estimated for each of the 16 runs for each cue type, containing adaptation estimates (signed) of all voxels in a given ROI. This resulted in a total of eight adaptation vectors per cue type. The secondary factor “environment” was averaged out by calculating the mean of two adaptation vectors from adjacent runs from different environments for the same cue type on a voxel-by-voxel basis (see a similar treatment of averaging out a factor not of primary interest in Shine et al., 2019).

Next, the adaptation pattern similarity was computed in a cross-validated manner by calculating Pearson’s correlation between pairwise adaptation vectors (Walther et al., 2016). Specifically, within-cue similarity was calculated as the Pearson’s correlation between adaptation vectors of the same cue type. In contrast, between-cue similarity was computed by correlating adaptation vectors from different cue types. The final estimates of within-cue similarity and between-cue similarity were obtained by averaging all Pearson’s correlations (Fisher transformed) calculated from all possible pairs of adaptation vectors.

Finally, we calculated an adaptation pattern distinction score by subtracting the between-cue similarity from the within-cue similarity. The adaptation pattern distinction score was then tested against zero with a directional one-sample *t* test, as we expected the adaptation pattern to be more similar within the same cue type than between different cue types. A positive adaptation pattern distinction score indicates that the spatially distributed adaptation pattern differed between different cue types.

To verify that our findings were not solely driven by a particular environment, we also conducted the analysis for each environment separately.

#### fMRI analysis of successful navigation

To assess the involvement of brain regions in the navigation task, we constructed GLM3 to compare brain activation during the “location occupation” phase between correct trials and incorrect trials. This approach is commonly employed to infer brain regions' engagement in cognitive tasks (Pessoa et al., 2002; Chrastil et al., 2015). To ensure consistency and effective comparisons, this analysis was conducted in exactly the same manner as in our previous study (Chen et al., 2019). In particular, the first-level GLMs were constructed in the same way as in our previous study.

##### First-level analysis

We constructed GLM3, which had a structure similar to GLM1, with three differences. First, there were no parametric regressors that modeled the modulatory effects of inter-location spatial relation on BOLD signals. Second, there were separate event regressors modeling the “location occupation phase” for the incorrect trials and the correct trials separately, collapsed across the four test locations. This resulted in 2 (landmark vs self-motion) * 2 (correct vs incorrect) = 4 event regressors. Third, the scans were concatenated across all the 16 runs, because some subjects did not make a single mistake in some runs, meaning that no trials could be labeled as “incorrect” in the run. In the scan concatenation, 16 effect regressors were included to model the average activity in each run, and each run had its own set of head motion regressors.

##### Second-level ROI-based analysis

For each ROI, mean beta estimates of brain activation during the “location occupation” phase were submitted to a repeated-measures ANOVA with cue type (landmark vs self-motion) and correctness (correct vs incorrect) as independent variables. Statistical outliers were identified using the boxplot rule; that is, a value would be considered as an outlier if it is larger than 3rd quartile + 3*interquartile range or smaller than 1st quartile − 3*interquartile range. Statistical outliers were then winsorized to the nearest inlier (Reifman and Keyton, 2010).

##### Second-level voxel-wise analysis

The participant-specific beta images were normalized to the MNI template. The normalized beta images were then spatially smoothed with a 3 mm full-width half-maximum (FWHM) Gaussian filter. To obtain participant-specific contrast images for evaluating the main effect of successful navigation in the within-subjects design in GLM3, we applied appropriate weighting schemes to the participant-specific normalized beta images, assigning weights [1, −1, 1, −1] to the beta images of the landmark correct, landmark incorrect, self-motion correct, and self-motion incorrect conditions. Next, similar to the univariate adaptation analysis, a nonparametric two-tailed one-sample *t* test was conducted on the contrast images for the main effect of successful navigation, using the SnPM13 toolbox (Nichols and Holmes, 2002). We created group-level anatomical masks for small volume correction (Extended Data 1, Section 1.4). To explore beyond our ROIs, we corrected for multiple comparisons across the entire volume.

## Results

### Behavioral results

#### Behavioral evidence for a dissociation of landmarks and self-motion cues

**Figure 3.**
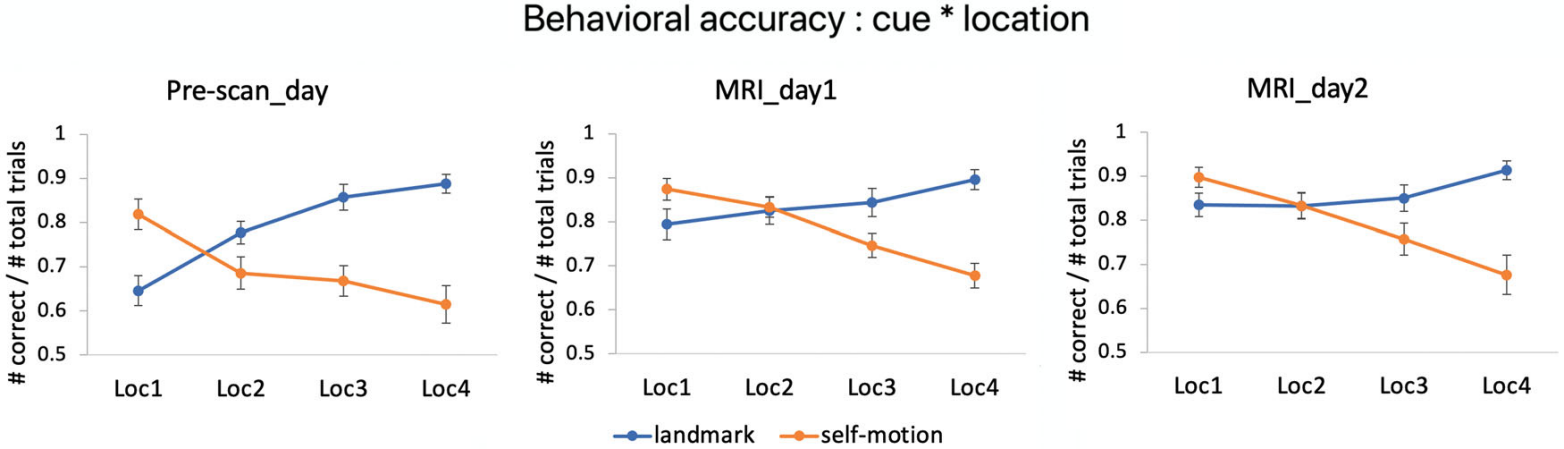
Behavioral results. Behavioral accuracy is plotted as a function of cue type and test location in each experimental day. Error bars represent ±S.E.

We focused on behavioral data obtained during the two scanning sessions (Fig. 3). We submitted behavioral accuracy to a repeated-measures ANOVA with cue type, test location, scanning session, and environment as independent variables. This analysis revealed the main effects of cue type (*F*_(1,19)_ = 10.552; *p* = 0.004, $\eta_{p}^{2}$ = 0.357)^a1, Table 1^ and location (*F*_(3,57)_ = 9.170; *p* < 0.001; $\eta_{p}^{2}$ = 0.326)^a2^, which were qualified by a significant interaction between the two variables (*F*_(3,57)_ = 25.051; *p* < 0.001; $\eta_{p}^{2}$^ ^= 0.569)^a3^: in the landmark condition, behavioral accuracy increased as the test location got closer to the tree (i.e., the anchoring point for landmark-based navigation), whereas in the self-motion condition, behavioral accuracy increased as the test location got closer to the arrows (i.e., the anchoring point for path integration). Accordingly, the interaction between cue type and the linear trend of test location was significant (*t*_(57)_ = 8.487; *p* < 0.001)^a4^. Further analyses revealed that the linear trend of test location was significant in both the landmark condition (*t*_(112)_ = 3.020; *p* = 0.003)^a5^ and the self-motion condition (*t*_(112)_ = 9.798; *p* < 0.001)^a6^. No effects involving environment or scanning session were significant (ps > 0.3; $\eta s_{p}^{2}$ < 0.062), meaning that behavioral performance was similar in the two environments and stable across the two scanning sessions.

Furthermore, we employed a cognitive modeling approach to investigate whether using different navigational cues specifically affected representational precision by disentangling representational precision, response bias, and potential attentional lapse. We found that representational precision exhibited a similar pattern as the behavioral accuracy as a function of cue type and test location, after accounting for response bias and attentional lapse (Extended Data 1, Section 2.1, Fig. E1).

Finally, further analysis that also included data from the Pre-scan_day showed that (1) performance had already reached ceiling toward the end of the Pre-scan_day and (2) performance remained stable across the two MRI scanning days (Extended Data 1, Section 2.2, Fig. E2).

In summary, the key behavioral finding was the distinct profiles of behavioral accuracy observed across test locations in the two cue conditions. Importantly, this finding was not confounded by response bias or attentional failure, indicating the success of our cue dissociation manipulation. Finally, participants' behavioral performance had reached a plateau prior to the MRI scanning.

### fMRI results

fMRI analyses focused on the location occupation phase of the location identification task, when the camera was panned down to the ground to render visual inputs identical between the landmark condition and the self-motion condition (Fig. 1*b*, Phase 4). We investigated whether BOLD signals contained information pertaining to the spatial relations among the test locations. To this end, we employed fMRI adaptation, a well-documented phenomenon in which BOLD signal exhibits a reduction when the current location is preceded by the same or a nearby location, with the degree of suppression proportional to the spatial proximity of the two locations (Morgan et al., 2011; Barron et al., 2016; Chen et al., 2019).

To evaluate a potential impact of scanning session and environment on adaptation, we conducted an omnibus repeated-measures ANOVA on adaptation, with ROI (RSC, HIPP, PRC, PHC, left alEC, left pmEC, right alEC, right pmEC), cue type, environment, and scanning session as independent variables. While the main effect of ROI was significant (*F*_(7,133)_ = 5.180; *p* < 0.001; $\eta_{p}^{2}$ = 0.214)^b^, there were no significant effects involving environment or scanning session (Fs < 3.1; ps > 0.09; $\eta s_{p}^{2}$ < 0.15), which mirrors the behavioral results. Therefore, we averaged adaptation effects across both environments and scanning sessions for all the further analyses.

#### Little evidence for positional coding for either landmarks or self-motion cues in EC

**Figure 4.**
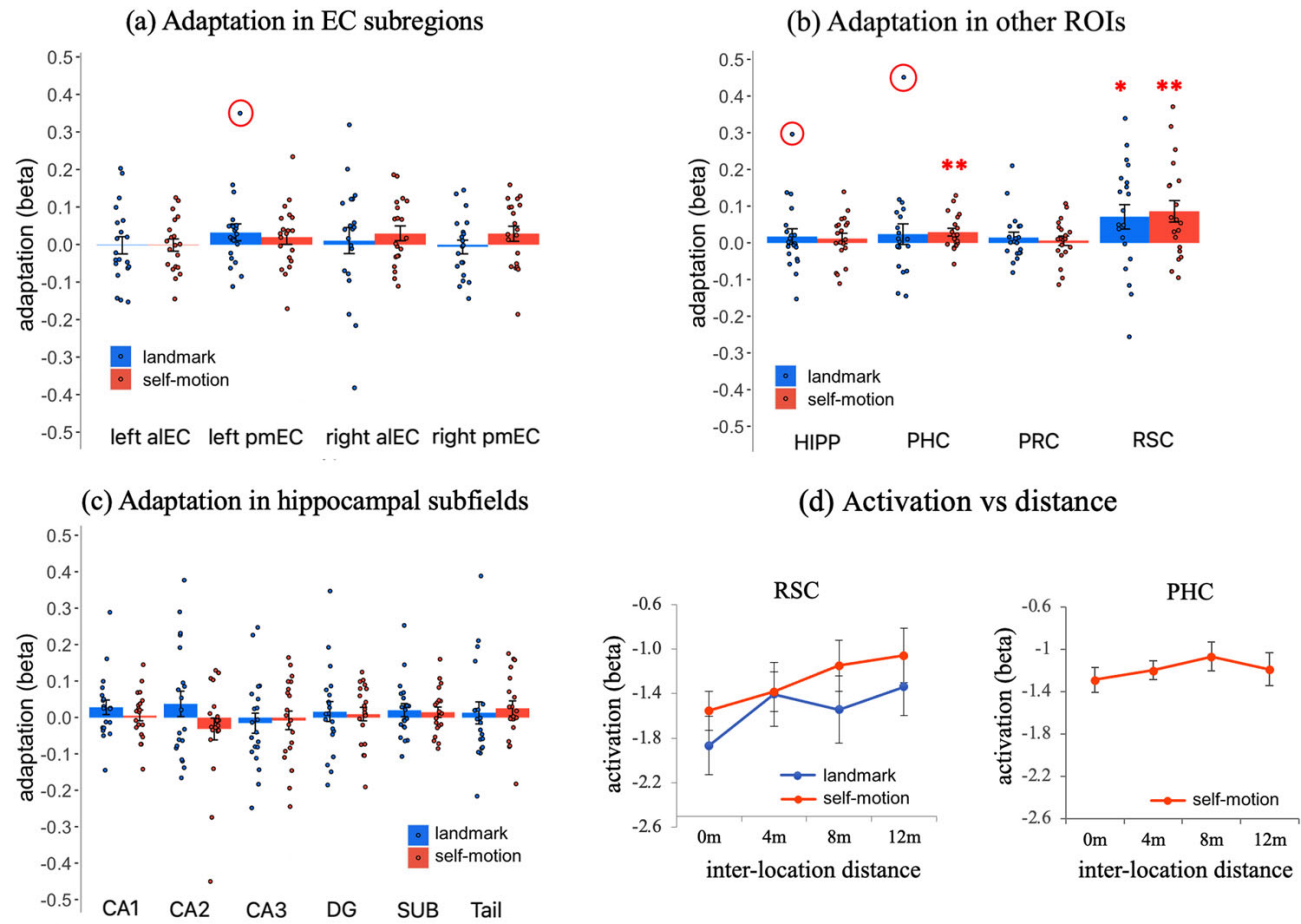
Adaptation in all ROIs. ***a***, Adaptation in the entorhinal subregions with inter-location distance modeled. ***b***, Adaptation in hippocampal subfields with inter-location distance modeled. ***c***, Adaptation in the RSC, HIPP, PHC, and PRC with inter-location distance modeled. ***d***, Visualization of significant adaptation effects in the RSC for both cue types and in the PHC for self-motion cues. The ROI's activation was plotted as a function of inter-location distance. Statistical outliers were highlighted in red circles, which were winsorized to the nearest inlier in statistical tests. Error bars represent ±SE. *p_1-tailed _< 0.05, **p_1-tailed _< 0.01. RSC, retrosplenial cortex; HIPP, hippocampus; PHC, parahippocampal cortex; PRC, perhirnal cortex; alEC, anterior-lateral entorhinal cortex; pmEC, posterior-medial entorhinal cortex. DG, dentate gyrus; SUB, subiculum.

First, we examined whether we could replicate our previous observation of adaptation-based positional coding in the entorhinal subregions (Chen et al., 2019). The ROI-based analysis revealed no significant adaptation in any of the entorhinal subregions in either hemisphere for either cue type, even at the uncorrected significance level (ts < 1.52; ps_1-tailed_ > 0.07; BFs_10_ < 1.14; Fig. 4*a*; see Table 2 for detailed statistics). Consistently, the voxel-wise analysis revealed no significant adaptation for either cue type in the left EC when using the group-level anatomical mask of the left EC for small volume correction (Ts < 4.5; ps_ FWE-corr_ > 0.1) and no significant adaptation for either cue type in the right EC when using the group-level anatomical mask of the right EC for small volume correction (Ts < 3; ps_ FWE-corr_ > 0.8).[Table T2]

**Table 1.** Statistical table

Description	Manuscript reference	Data structure	Type of test	*p* value	Effect size ( $\eta_{p}^{2}$) or Bayes factor (BF_10_)
Analysis of behavioral accuracy	a1	Normally distributed	*F* test, main effect	0.004	$\eta_{p}^{2}$ = 0.357
	a2	Normally distributed	*F* test, main effect	<0.001	$\eta_{p}^{2}$^ ^= 0.326
	a3	Normally distributed	*F* test, interaction effect	<0.001	$\eta_{p}^{2}$^ ^= 0.569
	a4	Normally distributed	*F* test, contrast	<0.001	N.A.
	a5	Normally distributed	*F* test, contrast	0.003	N.A.
	a6	Normally distributed	*F* test, contrast	0.001	N.A.
Effect of ROI on adaptation	b	Normally distributed	*F* test, main effect	<0.001	$\eta_{p}^{2}$ = 0.214
Analysis of adaptation in RSC	c1	Normally distributed	One-sided one-sample *t* test	0.023	BF_10_ = 2.843
	c2	Normally distributed	One-sided one-sample *t* test	0.004	BF_10_ = 11.769
	c3	Normally distributed	One-sided one-sample *t* test	0.002	BF_10 _= 25.919
	c4	Normally distributed	One-sided one-sample *t* test	0.032	BF_10_ = 2.191
	c5	Normally distributed	One-sided one-sample *t* test	0.030	BF_10_ = 2.353
	c6	Normally distributed	One-sided one-sample *t* test	0.464	BF_10_ = 0.249
Analysis of adaptation in the PHC	d	Normally distributed	One-sided one-sample *t* test	0.006	BF_10_ = 8.492
Analysis of adaptation across retrosplenial portions	e1	Normally distributed	*F* test, main effect	0.008	$\eta_{p}^{2}$= 0.120
	e2	Normally distributed	*F* test, contrast	<0.001	N.A.
	e3	Normally distributed	*F* test, interaction effect	0.415	$\eta_{p}^{2}$ = 0.050
	e4	Normally distributed	*F* test, main effect	0.833	$\eta_{p}^{2}$^ ^= 0.002
Comparing adaptation patterns between cue types, with environment averaged out	f1	Normally distributed	two-sided one-sample *t* test	0.011	BF_10_ = 4.799
	f2	Normally distributed	One-sided one-sample *t* test	0.005	BF_10_ = 10.625
	f3	Normally distributed	One-sided one-sample *t* test	0.007	BF_10_ = 7.694
	f4	Normally distributed	One-sided one-sample *t* test	0.779	BF_10_ = 0.141
	f5	Normally distributed	One-sided one-sample *t* test	0.867	BF_10_ = 0.120
Comparing adaptation patterns between cue types, in each environment	g1	Normally distributed	One-sided one-sample *t* test	0.035	BF_10_ = 2.035
	g2	Normally distributed	One-sided one-sample *t* test	0.009	BF_10_ = 6.474
	g3	Normally distributed	One-sided one-sample *t* test	0.466	BF_10_ = 0.248
	g4	Normally distributed	One-sided one-sample *t* test	0.636	BF_10_ = 0.182
	g5	Normally distributed	One-sided one-sample *t* test	0.029	BF_10_ = 2.406
	g6	Normally distributed	One-sided one-sample *t* test	0.034	BF_10_ = 2.093
	g7	Normally distributed	One-sided one-sample *t* test	0.778	BF_10_ = 0.142
	g8	Normally distributed	One-sided one-sample *t* test	0.856	BF_10_ = 0.123
Contributions of retrosplenial portions to cue-specific adaptation patterns	h	Normally distributed	*F* test, main effect	0.820	$\eta_{p}^{2}$ = 0.029
Comparing adaptation patterns between environments for each cue type	i1	Normally distributed	One-sided one-sample *t* test	0.588	BF_10 _= 0.198
	i2	Normally distributed	One-sided one-sample *t* test	0.009	BF_10_ = 6.400
	i3	Normally distributed	One-sided one-sample *t* test	0.013	BF_10 _= 4.564
	i4	Normally distributed	One-sided one-sample *t* test	0.008	BF_10_ = 7.004
Disentangling path length and location in adaptation in the RSC	j1	Normally distributed	One-sided one-sample *t* test	0.125	BF_10_ = 0.430
	j2	Normally distributed	One-sided one-sample *t* test	0.093	BF_10_ = 0.525
	j3	Normally distributed	One-sided one-sample *t* test	0.004	BF_10_ = 12.694
	j4	Normally distributed	One-sided one-sample *t* test	0.034	BF_10_ = 2.102
Disentangling path length and location in adaptation in the PHC	k1	Normally distributed	One-sided one-sample *t* test	0.324	BF_10_ = 0.342
	k2	Normally distributed	One-sided one-sample *t* test	0.126	BF_10_ = 0.739
Comparing DP_rel_ between location and path length	l	Normally distributed	*F* test, main effect	<0.001	$\eta_{p}^{2}$ = 0.326
Disentangling location and subjective response in adaptation in the RSC	m1	Normally distributed	One-sided one-sample *t* test	0.454	BF_10_ = 0.254
	m2	Normally distributed	One-sided one-sample *t* test	0.778	BF_10_ = 0.143
	m3	Normally distributed	One-sided one-sample *t* test	0.025	BF_10_ = 2.694
	m4	Normally distributed	One-sided one-sample *t* test	0.023	BF_10_ = 2.892
Disentangling location and response in adaptation in the PHC	n1	Normally distributed	One-sided one-sample *t* test	0.283	BF_10_ = 0.383
	n2	Normally distributed	One-sided one-sample *t* test	0.139	BF_10_ = 0.683
Comparing DP_rel_ between location and response	o	Normally distributed	*F* test, main effect	<0.001	$\eta_{p}^{2}$ = 0.500
Disentangling location and temporal distance in adaptation in the RSC	p1	Normally distributed	One-sided one-sample *t* test	0.002	BF_10_ = 28.206
	p2	Normally distributed	One-sided one-sample *t* test	0.003	BF_10_ = 15.359
	p3	Normally distributed	One-sided one-sample *t* test	0.128	BF_10_ = 0.728
	p4	Normally distributed	One-sided one-sample *t* test	0.028	BF_10_ = 2.488
	p5	Normally distributed	One-sided one-sample *t* test	0.107	BF_10_ = 0.841
Disentangling location and temporal distance in adaptation in the PHC	q1	Normally distributed	One-sided one-sample *t* test	0.032	BF_10_ = 2.186
	q2	Normally distributed	One-sided one-sample *t* test	0.016	BF_10_ = 3.841
Evaluation of influences of passive movement phase on adaptation	r1	Normally distributed	One-sided one-sample *t* test	<0.001	BF_10 _= 43.706
	r2	Normally distributed	One-sided one-sample *t* test	0.003	BF_10 _= 13.818
	r3	Normally distributed	One-sided one-sample *t* test	0.006	BF_10_ = 8.572
	r4	Normally distributed	One-sided one-sample *t* test	0.007	BF_10_ = 7.511
	r5	Normally distributed	One-sided one-sample *t* test	0.001	BF_10_ = 31.856
	r6	Normally distributed	One-sided one-sample *t* test	0.003	BF_10_ = 17.435
	r7	Normally distributed	One-sided one-sample *t* test	0.005	BF_10_ = 9.724
	r8	Normally distributed	One-sided one-sample *t* test	0.011	BF_10 _= 5.400
	r9	Normally distributed	One-sided one-sample *t* test	0.007	BF_10_ = 7.880
Analysis of successful navigation effect	s1	Normally distributed	*F* test, main effect	0.001	$\eta_{p}^{2}$ = 0.476
	s2	Normally distributed	*F* test, main effect	0.021	$\eta_{p}^{2}$^ ^= 0.248
	s3	Normally distributed	*F* test, main effect	0.042	$\eta_{p}^{2}$ = 0.201

**Table 2. T2:** Adaptation in all ROIs

Brain region	Cue	Mean	*t*	df	*p*_1-tailed_	BF_10_
**RSC**	**Landmark**	**0**.**071**	**2**.**127**	**19**	**0**.**023**	**2**.**843**
	**Landmark^%^**	**0**.**098**	**3**.**356**	**19**	**0**.**002**	**25**.**919**
	**Self-motion**	**0**.**086**	**2**.**940**	**19**	**0**.**004**	**11**.**769**
HIPP	Landmark	0.017	0.811	19	0.214	0.480
	Landmark^#^	0.009	0.547	19	0.295	0.370
	Self-motion	0.012	0.830	19	0.208	0.489
**PHC**	Landmark	0.024	0.844	19	0.205	0.499
	Landmark^#^	0.007	0.393	19	0.349	0.321
	**Self-motion**	**0**.**029**	**2**.**759**	**19**	**0**.**006**	**8**.**492**
PRC	Landmark	0.014	0.958	19	0.175	0.568
	Self-motion	0.006	0.445	19	0.331	0.335
Right alEC	Landmark	0.011	0.305	19	0.382	0.297
	Self-motion	0.030	1.516	19	0.073	1.136
Right pmEC	Landmark	−0.007	−0.361	19	0.639	0.181
	Self-motion	0.028	1.421	19	0.172	1.013
Left alEC	Landmark	−0.002	−0.092	19	0.536	0.217
	Self-motion	−0.001	−0.091	19	0.536	0.217
Left pmEC	Landmark	0.033	1.454	19	0.081	1.042
	Landmark^#^	0.023	1.387	19	0.091	0.958
	Self-motion	0.020	1.012	19	0.162	0.606
CA1	Landmark	0.028	1.401	19	0.089	0.976
	Self-motion	0.006	0.372	19	0.357	0.315
CA2	Landmark	0.037	1.065	19	0.150	0.641
	Self-motion	−0.032	−1.056	19	0.848	0.125
CA3	Landmark	−0.016	−0.574	19	0.714	0.159
	Self-motion	−0.008	−0.315	19	0.622	0.187
DG	Landmark	0.016	0.594	19	0.280	0.387
	Self-motion	0.009	0.494	19	0.313	0.351
SUB	Landmark	0.020	1.108	19	0.141	0.675
	Self-motion	0.014	0.998	19	0.165	0.593
Tail	Landmark	0.012	0.411	19	0.343	0.326
	Self-motion	0.025	1.207	19	0.121	0.761

*p*_1-tailed_ represents the statistical significance one-tailed one-sample *t* test on adaptation. In correspondence to Figure 4. Significant results are highlighted in bold. BF_10_ represents the Bayes factor showing evidence for the alternative hypothesis over the null hypothesis. “Landmark^#^” represents when the statistical outlier was winsorized. “Landmark^%^” represents when location identity was modeled in adaptation in the RSC for the landmark condition. In all other cases, inter-location distance was modeled in adaptation.

#### The RSC shows strong evidence for positional coding for both landmarks and self-motion cues

Next, we examined whether adaptation existed in other ROIs, that is, RSC, HIPP, PHC, and PRC. The results are depicted in Figure 4*b*, and detailed statistics can be found in Table 2.

**Figure 5.**
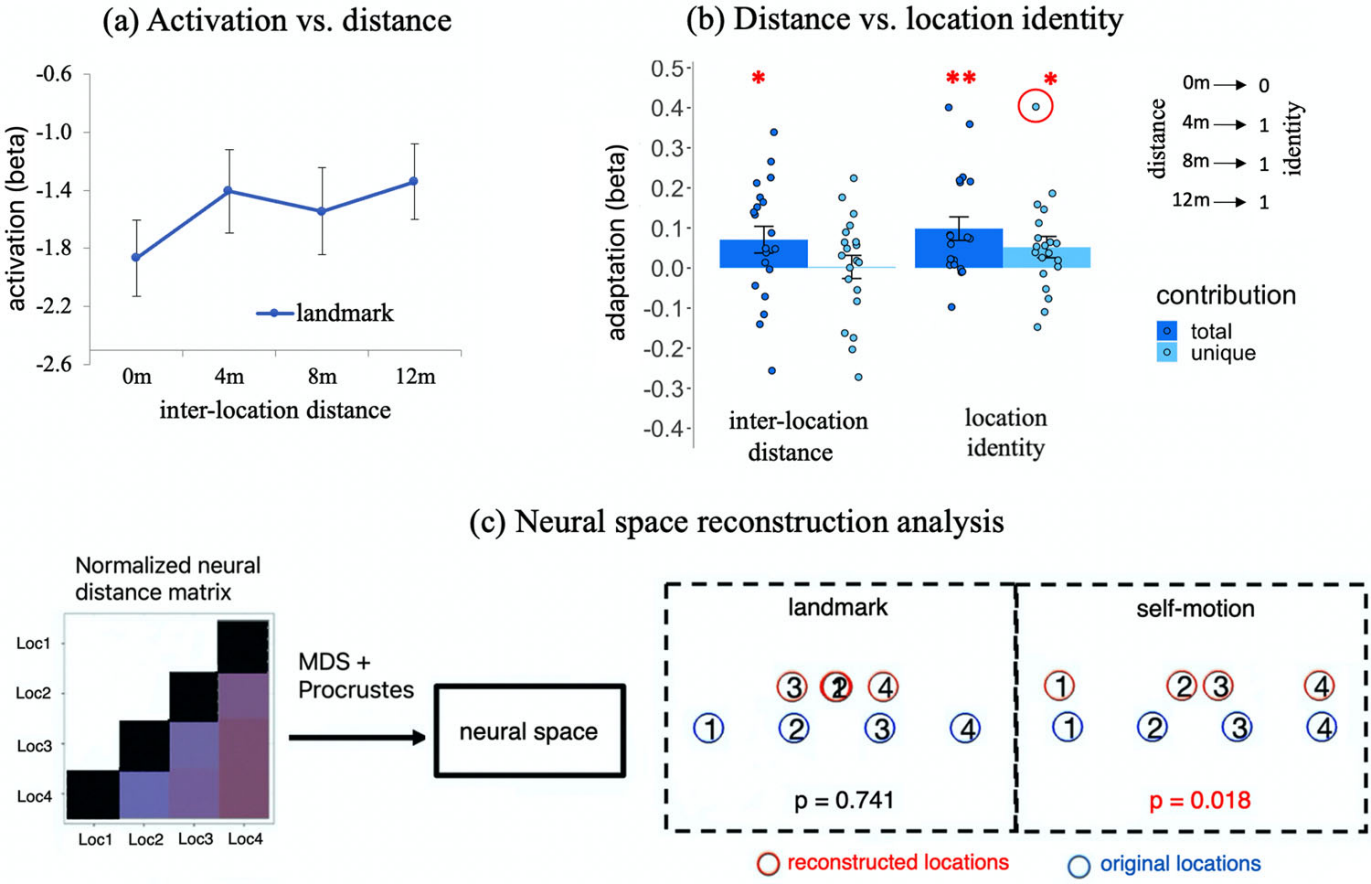
Contrasting inter-location distance and location identity in adaptation in the RSC. ***a***, Visualization of adaptation effect in the RSC for landmarks. The ROI’s activation is plotted as a function of inter-location distance, same as Figure 4*d* (left, landmark). ***b***, Disentangling inter-location distance and location identity in adaptation for landmarks. The total contribution and unique contribution of inter-location distance and location identity are plotted. The unique contribution represents the contribution of the variable after the other variable has been accounted for. ***c***, The neural space reconstruction analysis. The multidimensional scaling analysis and Procrustes analysis were applied to the normalized neural distance matrix calculated from trial-wise activation levels of the brain region. The reconstructed neural space (red circles) was compared with the physical space (blue circles). Significant results (*p* < 0.05) indicate resemblance of the neural space to the physical space.

The RSC showed significant adaptation in both the landmark condition and the self-motion condition (landmark, *t*_(19)_ = 2.127, *p*_1-tailed _= 0.023, BF_10_ = 2.843^c1^; self-motion, *t*_(19)_ = 2.940, *p*_1-tailed_ = 0.004, BF_10_ = 11.769 > 10^c2^). As shown in Figure 4*d* (left), the RSC’s activation increased linearly with increasing inter-location distance in the self-motion condition. However, the landmark condition exhibited a different pattern: the RSC’s activation was higher for all nonzero inter-location distances relative to the zero inter-location distance (i.e., when two successively visited locations were the same) but remained stable for different nonzero distances. This implies that in the landmark condition, RSC adaptation was related to location identity rather than continuous inter-location distance. Therefore, we modeled location identity by setting the value of the parametric regressor to 0 if the preceding test location was the same as the current one and to 1 if the preceding test location was different from the current one; we observed strong evidence for location-identity-based adaptation for landmarks (*t*_(19)_ = 3.356; *p*_1-tailed _= 0.002; BF_10 _= 25.919 > 10 ^c3^), as shown in Figure 5*b* (dark blue bar). Furthermore, when we disentangled location identity and inter-location distance in the same first-level GLM by including both variables as the parametric modulation regressors (Mumford et al., 2015), the unique contribution of location identity to adaptation—after accounting for inter-location distance—was significant (*t*_(19)_ = 1.963, *p*_1tailed _= 0.032, BF_10_ = 2.191^c4^; one outlier winsorized: *t*_(19)_ = 2.008, *p*_1tailed _= 0.030, BF_10_ = 2.353^c5^; light blue bar). In contrast, the unique contribution of inter-location distance—after accounting for location identity—was not significant (*t*_(19)_ = 0.093; *p*_1tailed _= 0.464; BF_10_ = 0.249^c6^). These results indicate that adaptation was predominantly driven by location identity rather than inter-location distance for landmarks in the RSC.

Besides the RSC, the PHC showed significant adaptation in the self-motion condition (*t*_(19)_ = 2.759; *p*_1-tailed_ = 0.006; BF_10_ = 8.492 ^d^), as shown in Figure 4*b*. In this condition, PHC activation increased linearly as inter-location distance increased, although there was a slight drop at the longest inter-location distance (=12 m; Fig. 4*d*, right). However, unlike the RSC, the unique contribution of either location identity or inter-location distance was not significant (ts < 0.9; ps > 0.2; BFs_10_ < 0.51), meaning these two forms of adaptation could not be disentangled.

Other ROIs (PRC, HIPP, and hippocampal subfields) did not show significant adaptation, even at the uncorrected significance level (ts < 1.5; ps_ _> 0.08; BFs_10_ < 1; Fig. 4*b*,*c*).

To ascertain the robustness of the adaptation effects observed in the RSC and PHC, we employed a nonparametric maximum *t* statistic multiple-comparisons correction across all four ROIs and both cue types, resulting in a total of eight one-sample *t* tests in total (Extended Data 1, Section 1.3). All the aforementioned significant adaptation effects survived the multiple-comparisons correction: landmark condition (*p*_corrected_ = 0.005) and self-motion condition (*p*_corrected_ = 0.022) in the RSC and self-motion condition in the PHC (*p*_corrected_ = 0.033) exhibited robust statistical significance.

**Figure 6.**
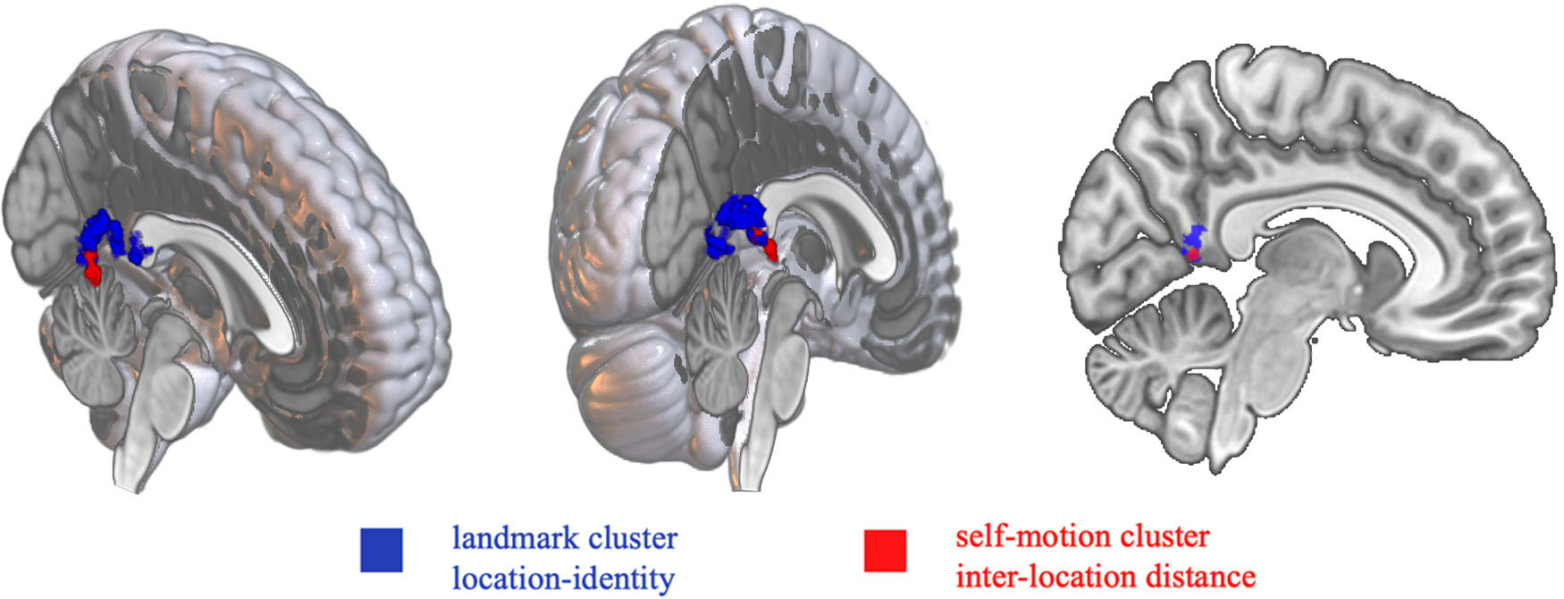
Retrosplenial clusters showing significant location-based adaptation. The two clusters are rendered in the MNI template brain, viewed from the front (left) and the back (middle) in the 3D brain, as well as in the sagittal view of the brain (right). The clusters are within the group-level anatomical mask of the RSC. The cluster-defining threshold is *T* > 3. The landmark cluster consists of 1,029 voxels (blue), and the self-motion cluster consists of 153 voxels (red). The adaptation reflects location identity for landmarks and inter-location distance for self-motion cues.

Finally, to further investigate the robustness of the adaptation effects observed in the RSC and PHC in the ROI-based analysis, we conducted voxel-wise analysis, using a group-level mask that encompassed all of our ROIs (i.e., bilateral MTL plus RSC) for small volume correction. In the landmark condition, we observed a significant cluster showing location identity-based adaptation (*k* = 1,029; *p*_FWE-corr_ < 0.001; Fig. 6), and the peak voxel was within the group-level anatomical mask of the RSC (*T* = 6.76; MNI: 3, −56, 12). Notably, this peak voxel within the RSC mask also represents the second highest level of statistical significance across the entire search volume and was included in a larger cluster that survived the multiple-comparisons correction across the entire search volume (*K* = 4,085; *p*_FWE-corr_ = 0.002). This analysis also revealed two other clusters showing significant location identity-based adaptation in the parahippocampal, lingual, and fusiform gyri. Conversely, the analysis of inter-location distance-based adaptation did not yield any significant results (ks < 53; ps_FWE-corr _> 0.25), which mirrors the ROI-based analysis. In the self-motion condition, we observed one significant cluster associated with inter-location distance-based adaptation (*k* = 153; *p*_ FWE-corr_ = 0.015). The peak voxel was within the group-level anatomical mask of the RSC (Fig. 6). Conversely, no significant clusters were found for location identity-based adaptation (ks < 90; ps_FWE-corr _> 0.06). In brief, the voxel-wise analysis confirmed the ROI-based analysis that revealed significant location identity-based adaptation for landmarks and inter-location distance-based adaptation for self-motion cues in the RSC. Detailed results of the voxel-wise analysis are summarized in Extended Data 1 (Section 2.3, Fig. E4, Table E1).

We also compared the two hemispheres for the RSC and HPC in adaptation and obtained very similar results in the two hemispheres (Extended Data 1, Fig. E5).

#### Successful reconstruction of physical space from adaptation in the RSC for self-motion cues

To further characterize the abovementioned significant adaptation effects observed in the RSC and PHC, we asked whether it was possible to reconstruct individual test locations from adaptation effects in these areas. If fine-grained distance information between different locations was contained in the adaptation effect, we should be able to recover the physical space from it. This analysis can also further illustrate the aforementioned distinction between location identity-based adaptation and inter-location distance-based adaptation, because only the latter form should contain fine-grained distance information to allow for a successful reconstruction of the physical space.

To address this question, we employed a neural space reconstruction analysis (Fig. 5*c*) that was based on neural distances between different test locations. Neural distance was quantified as a brain region's activation for one location when preceded by another. Lower activation levels correspond to stronger repetition suppression and closer positioning of two test locations in the neural space (Extended Data 1, Section 1.5).

Regarding the RSC, in the landmark condition, the neural representation of space did not correspond to the physical space (*p*_1-tailed_ = 0.741), with some locations even swapped in order. This observation aligns with the earlier finding that in the landmark condition, the repetition suppression effect only distinguished between identical and different locations, without discerning between different nonzero inter-location distances (Fig. 5*c*). In contrast, in the self-motion condition, the neural representation of space demonstrated a significant resemblance to the physical space (*p*_1-tailed _= 0.018). This finding is consistent with the earlier observation that in the self-motion condition, the repetition suppression effect contained fine-grained distance information between different locations (Fig. 5*c*).

Although the preceding results showed that the PHC exhibited significant inter-location distance-based adaptation for self-motion cues, the corresponding reconstructed neural space did not significantly resemble the physical space (*p*_1-tailed _= 0.252).

In brief, we could reconstruct the original physical space from adaptation in the RSC with respect to self-motion cues, but not based on landmarks. This underscores again that adaptation in this region reflected continuous inter-location distance for self-motion cues but not for landmarks. The inability to reconstruct the physical space through adaptation in the PHC for self-motion cues highlights the pivotal role of the RSC in faithfully representing fine-grained distance information extracted from self-motion cues.

#### Positional coding in the RSC is distinct between cue types

To iterate, the primary goal of this study was to assess the specificity or independence of spatial representations in the brain with respect to landmarks and self-motion cues. The preceding analyses revealed spatial coding for both landmarks and self-motion cues in the RSC. As depicted in Figure 6, the retrosplenial clusters exhibiting significant adaptation for landmarks and self-motion cues displayed little overlap. However, it remains to be determined whether the spatial coding in the RSC was cue-specific or cue-independent. To answer this question, we conducted two main types of analyses: univariate analysis in either the standard brain or participants' native brains and multivariate analysis leveraging spatially fine-grained signals across all voxels within the RSC.

##### Univariate analysis in standard brain

First, we tested differences between cue types in location-based adaptation within the RSC. We normalized participant-specific beta images to the MNI template, which were then spatially smoothed. We then conducted a nonparametric permutation-based paired *t* test, using the group-level anatomical mask of the RSC for small volume correction. There were no voxels (ps_FWE-corr_ > 0.2, 1-tailed) or clusters (ps_FWE-corr_ > 0.45, 1-tailed; cluster-defining threshold: *T* > 3) showing significant differences between cue types, meaning no positive evidence for cue-specific spatial representations in the RSC.

Next, we tested commonalities between cue types in location-based adaptation within the RSC, by conducting the conjunction analysis as implemented in the flexible factorial design in SPM12 (Friston et al., 1999). The group-level anatomical mask of the RSC was used for small volume correction. There were no voxels (ps_FWE-corr_ > 0.35, 1-tailed) or clusters (ps_FWE-corr_ > 0.14, 1-tailed; cluster-defining threshold: *T* > 3) showing significantly location-based adaptation for both cue types, meaning no positive evidence for cue-independent spatial representations in the RSC.

In brief, the univariate analyses in the standard brain revealed no positive evidence for either cue-specific or cue-independent spatial representation in the RSC.

##### Univariate analysis in native brain

The preceding univariate analyses were voxel-based and performed in a standard template (i.e., MNI template), which overlooks individual brain variability and may introduce spatial smoothing artifacts. To address these limitations, we conducted another univariate analysis in participants' native brains. Specifically, we segmented the RSC in a biologically valid way in each participant's brain; if different cue conditions exhibit differential patterns of adaptation change across the RSC portions, it would indicate that different parts of the RSC contained spatial coding for different cue types.

Currently, there are no established standards for segregating the human RSC (Vann et al., 2009). To achieve biologically meaningful segmentation, we employed the *ConGrads* toolbox (Haak et al., 2018), which is a data-driven approach that partitions a brain region into functionally discrete subregions based on voxel-to-voxel functional connectivity patterns (Schröder et al., 2015; Grady, 2020).

**Figure 7.**
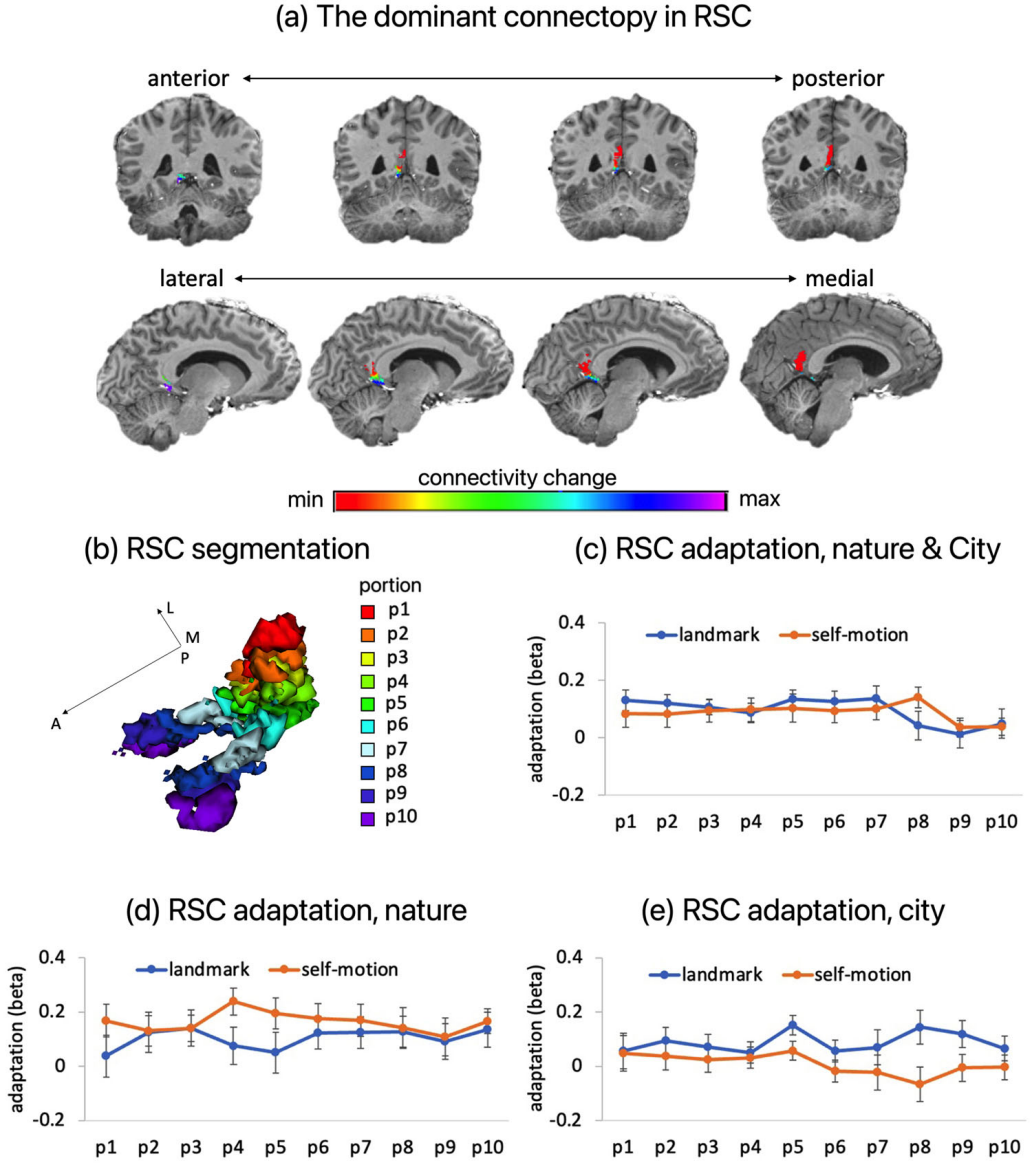
Functional segmentation of the RSC and location-based adaptation. ***a***, Visualization of the gradient in the RSC in the current study, based on the dominant connectopy. ***b***, The RSC was divided into 10 equally sized portions, based on the gradient shown in ***a***. ***c***, fMRI adaptation for landmarks (location identity based) and self-motion cues (inter-location distance based) for the 10 retrosplenial portions, averaged across the nature environment and the city environment. ***d***, fMRI adaptation for landmarks (location identity based) and self-motion cues (inter-location distance based) for the 10 retrosplenial portions, in the nature environment. ***e***, fMRI adaptation for landmarks (location identity based) and self-motion cues (inter-location distance based) for the 10 retrosplenial portions, in the city environment.

First, we applied the *ConGrads* toolbox to the participant-specific residual scans acquired from GLM1 as a proxy for resting-state functional data (Extended Data 1, Section 1.6). In the RSC, we observed a gradient along its long axis, as dictated by the dominant connectopy (Fig. 7*a*), which is in broad concordance with previous research (Chrastil, 2018; Peer et al., 2019). Notably, we obtained a consistent pattern of results when applying the *ConGrads* toolbox to a separate functional resting-state dataset acquired at a 3 T MRI scanner (Extended Data 1, Sections 1.6 and 2.5; Fig. E6).

Next, we divided the RSC into 10 equally-sized portions for each participant based on the dominant mode of the functional connectivity change (Fig. 7*b*). We then submitted location-based adaptation into a repeated-measures ANOVA test, with cue type, portion, and environment as independent variables. As shown in Figure 7*c–e*, the main effect of portion was statistically significant (*F*_(1,171)_ = 2.589; *p* = 0.008; $\eta_{p}^{2}$ = 0.120)^e1^, driven by relatively lower adaptation in the two anterior portions than the eight posterior portions (*t*_(174)_ = 4.536; *p* < 0.001)^e2^; the strength of adaptation was very close across the eight posterior portions. Critically, the interaction between cue type and portion was not significant (*F*_(9,171)_ = 1.010; *p* = 0.415; $\eta_{p}^{2}$ = 0.050)^e3^. The main effect of cue type was not significant (*F*_(1,19)_ = 0.046; *p* = 0.833; $\eta_{p}^{2}$ = 0.002)^e4^. None of the effects involving environment were significant (Fs < 2.6; ps > 0.1; $\eta s_{p}^{2}$ < 0.15). These results mean that (1) overall, adaptation effects were rather distributed across a large part of the RSC (i.e., 80% of the RSC toward its posterior end) and (2) adaptation change along the long axis of the RSC was similar between the cue types.

To summarize, the univariate analyses conducted in participants' native brains revealed no positive evidence for cue specificity of the positional coding in the RSC.

##### Multivariate analysis

The preceding univariate analyses, both in the standard space and the native space, revealed no significant differences or commonalities between landmarks and self-motion cues in adaptation. It is well-documented that fMRI univariate analyses are relatively limited in statistical power (Norman et al., 2006), because fine-grained spatial information across voxels is blurred and potential contributions from voxels with subthreshold signals are ignored. To address these limitations, we adopted the multivariate analysis, which offers greater statistical power by overcoming the limitations of the univariate analysis. Moreover, it is possible that the underlying neural units coding positional information for different cue types are spatially intermixed across the entire region at a relatively high frequency, a pattern that is detectable only through the multivariate approach.

**Figure 8.**
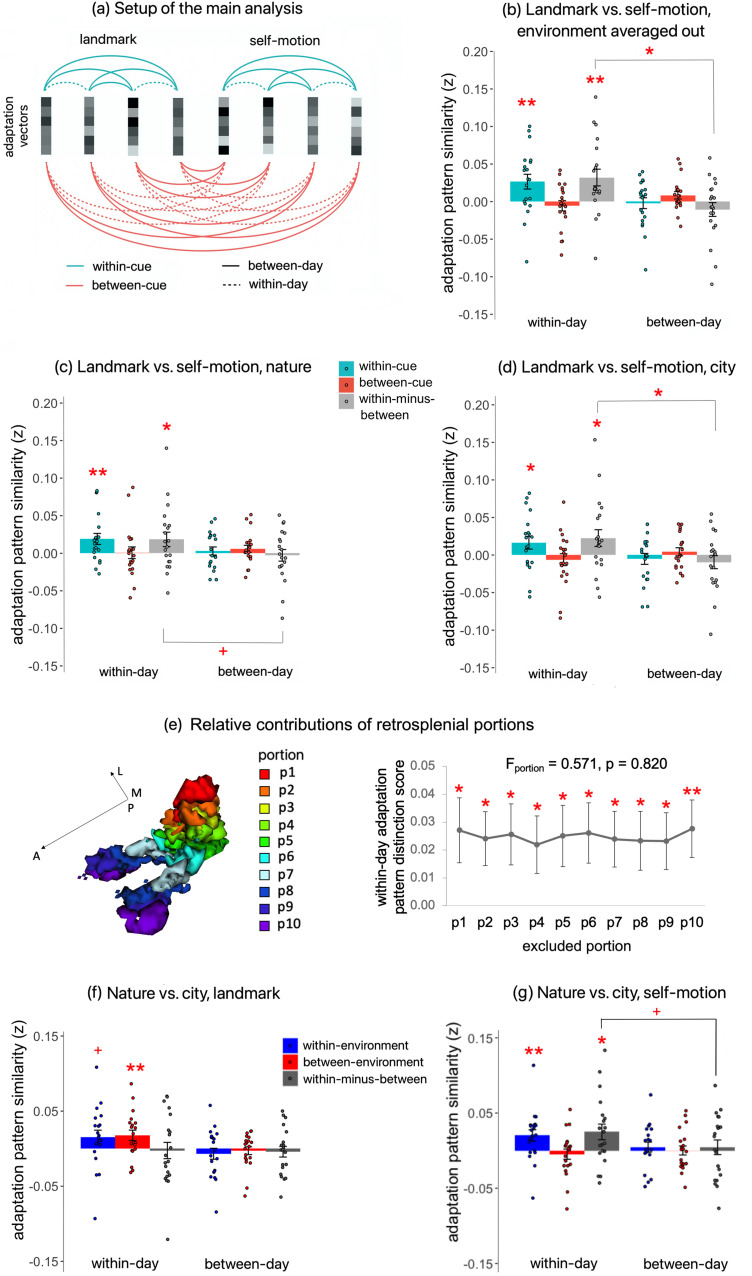
Results of fMRI adaptation pattern similarity analysis. ***a***, Setup of the fMRI adaptation pattern similarity analysis. The adaptation vectors were paired up to one another, resulting in 2 × 2 = 4 different types of pairing: cue relation (within-cue vs between-cue) × day relation (within-day vs between-day). ***b***, Adaptation patterns were compared between cues, with the “environment” factor averaged out. Adaptation pattern similarity is plotted as a function of cue relation and day relation. The adaptation pattern distinction score (within-minus-between, gray bars) equals to within-cue similarity (light blue bars) minus between-cue similarity (salmon bars). ***c***, Results in the nature environment, comparing adaptation patterns between cues. ***d***, Results in the city environment, comparing adaptation patterns between cues. ***e***, Assessing relative contributions of individual retrosplenial portions to the overall within-day adaptation pattern distinction score, which corresponds to scores indicated by the gray bars in ***b***, ***c***, and ***d***. The left panel shows RSC segmentation based on the gradient of the dominant connectopy, same as in Figure 7*b*. The right panel shows the within-day adaptation pattern distinction score as a function of the left-out portion. The scores were all significantly >0 and did not differ from one another. ***f***, Results for landmark cues, comparing adaptation patterns between the two environments. ***g***, Results for self-motion cues, comparing adaptation patterns between the two environments. Error bars represent ±SE. *p_1-tailed/2-tailed _< 0.05, **p_1-tailed/2-tailed _< 0.01, ^+^*p*_1-tailed/2-tailed _< 0.1.

To reiterate, in the multivariate analysis, we assessed the similarity of the multi-voxel adaptation patterns (Fig. 8*a*; Materials and Methods); that is, we determined whether voxels showing higher adaptation for one cue also showed higher adaptation for the other cue. Specifically, we derived an adaptation pattern distinction score, which was quantified as within-cue similarity (cross-validated Pearson’s correlation between adaptation vectors of the same cue type) minus between-cue similarity (cross-validated Pearson’s correlation between adaptation vectors of different cue types; Walther et al., 2016). The adaptation pattern distinction score was then tested against zero with a one-sided *t* test, as we expected the adaptation pattern to be more similar within the same cue type than between different cue types. A pattern distinction score significantly >0 would indicate that the across-voxel adaptation pattern was distinct between the two cue types. Given the preceding results showing that the adaptation in the RSC for landmark was predominately driven by location identity, we adopted the location-identity-based adaptation for landmarks to increase sensitivity of the following statistical analyses.

First, we assessed cue-specificity/generalizability of positional coding while averaging across environments (Shine et al., 2019), because “environment” served as a secondary factor in the experimental design and the preceding results showed no significant influences of this factor. As shown in Figure 8*b*, since the within-day pattern distinction score was significantly greater than the between-day distinction score (*t*_(19)_ = 2.825; *p*_2-tailed_ = 0.011; BF_10_ = 4.799^f1^; gray bars), we tested them separately. The within-day pattern distinction score was significantly >0 (*t*_(19)_ = 2.885; *p*_1-tailed_ = 0.005; BF_10_ = 10.625^f2^; gray bar), because the within-cue similarity was significantly positive (*t*_(19)_ = 2.708; *p*_1-tailed_ = 0.007; BF_10_ = 7.694^f3^; light blue bar) while the between-cue similarity was not (*t*_(19)_ = −0.807; *p*_1-tailed_ = 0.779; BF_10_ = 0.141^f4^; salmon bar). In other words, while the adaptation pattern was stable for a given cue type, it differed between the two cue types. In contrast, the between-day pattern distinction score was not significantly >0 (*t*_(19)_ = −1.145; *p*_1-tailed_ = 0.867; BF_10_ = 0.120^f5^; gray bar).

To exclude the possibility that the aforementioned cue-specificity finding was solely driven by one of the two environments, we examined the two environments separately. The two environments showed a consistent pattern of results. In the nature environment (Fig. 8*c*), the within-day adaptation pattern distinction score was significantly >0 (*t*_(19)_ = 1.915; *p*_1-tailed_ = 0.035; BF_10_ = 2.035^g1^; gray bar), because the within-cue similarity was significantly positive (*t*_(19)_ = 2.611; *p*_1-tailed_ = 0.009; BF_10_ = 6.474^g2^; light blue bar) while the between-cue similarity was not (*t*_(19)_ = 0.086; *p*_1-tailed_ = 0.466; BF_10_ = 0.248^ g3^; salmon bar). In contrast, the between-day pattern distinction score was not significantly >0 (*t*_(19)_ = −0.354; *p*_1-tailed_ = 0.636; BF_10_ = 0.182^g4^; gray bar). Similarly, in the city environment (Fig. 8*d*), the within-day pattern distinction score was significantly >0 (*t*_(19)_ = 2.022; *p*_1-tailed_ = 0.029; BF_10_ = 2.406^g5^; gray bar), because the within-cue similarity was significantly positive (*t*_(19)_ = 1.933; *p*_1-tailed_ = 0.034; BF_10_ = 2.093^g6^; light blue bar) while the between-cue similarity was not (*t*_(19)_ = −0.782; *p*_1-tailed_ = 0.778; BF_10_ = 0.142^g7^; salmon bar). In contrast, the between-day pattern distinction score was not significantly >0 (*t*_(19)_ = −1.092; *p*_1-tailed_ = 0.856; BF_10_ = 0.123^g8^; gray bar). Together, these results mean that in both environments, within the same scanning day, the adaptation pattern was stable for a given cue type but differed between different cue types.

To characterize the anatomical distribution of cue-specific adaptation in the RSC, we segmented the RSC into 10 equally-sized portions based on the dominant connectopy (Fig. 8*e*), as described previously. We then assessed the contribution of each RSC portion to the within-day adaptation pattern distinction score by leaving it out from the calculation (i.e., the jackknife procedure). The resulting scores were subjected to a repeated-measures ANOVA test, with portion as the independent variable. The main effect of portion was not significant (*F*_(9,171)_ = 0.571; *p* = 0.820; $\eta_{p}^{2}$. = 0.029)^h^, meaning comparable contributions among the 10 portions to the overall adaptation pattern distinction score. Interestingly, this finding contrasts with the earlier finding of lower univariate mass adaptation effects in the two anterior portions compared to the eight posterior portions of the RSC, meaning that portions lower in adaptation contributed to discriminating different cue conditions as comparably as the other portions. Together, our results suggest that the positional coding within the RSC was rather distributed anatomically, probably with neural units coding positional information for different cue types intermixed at a relatively high spatial frequency.

Our task design also allowed us to evaluate whether adaptation patterns were specific or generalizable between different environments for each cue type, although this was not our main interest of inquiry. For completeness, we compared adaptation patterns between different environments for each cue type. As shown in Figure 8*f*, in the landmark condition, the adaptation pattern distinction score within the same day was not significantly >0 (*t*_(19)_ = −0.226; *p*_1-tailed _= 0.588; BF_10 _= 0.198)^i1^, mainly due to the fact that the between-environment adaptation pattern similarity was significantly >0 (*t*_(19)_ = 2.604; *p*_1-tailed _= 0.009; BF_10_ = 6.400)^i2^. This result means that the adaptation pattern was significantly correlated between the two environments, indicating environment-independent spatial representations for landmarks. On the contrary, as shown in Figure 8*g*, in the self-motion condition, the adaptation pattern distinction score within the same day was significantly >0 (*t*_(19)_ = 2.411; *p*_1-tailed _= 0.013; BF_10_ = 4.564)^i3^, meaning distinct adaptation patterns between environments; this result was mainly driven by the within-environment adaptation similarity score being significantly >0 (*t*_(19)_ = 2.655; *p*_1-tailed_ = 0.008; BF_10_ = 7.004)^i4^.

To summarize, we observed distinct voxel-to-voxel adaptation patterns between landmarks and self-motion cues in the RSC: adaptation patterns were correlated within the same cue type but uncorrelated between different cue types. This effect occurred within the same scanning session and was regardless of the environment in which these cues were experienced. In addition, this cue-specific positional coding within the RSC seemed to be anatomically distributed across the region. Intriguingly, while the adaptation pattern was similar between environments for landmarks, it differed between environments for self-motion cues. This finding, however, does not undermine our interpretation of the main finding regarding distinct adaptation patterns between cue types. Since adaptation patterns were uncorrelated between cue types in each environment in the first place, the mean adaptation patterns averaged across environments would remain uncorrelated between cue types, regardless of whether the adaptation patterns are correlated or uncorrelated between environments for each cue type. Instead, this finding indicates that our multivariate analysis is sensitive to both the specificity and commonality in neural representations across experimental conditions. Together, our results demonstrate that spatial representations in the RSC were cue-specific and were sensitive to the physical features of the spatial inputs.

##### Summary

While univariate analyses did not reveal any significant differences or commonalities in adaptation between cue types, the more sensitive multivariate analysis unveiled cue-specific positional coding in the RSC, meaning that the voxel-to-voxel adaptation pattern was distinct between the cue types. These findings indicate that the underlying neural units responsible for encoding positional information for different cue types are probably intermingled at a relatively high spatial frequency in the RSC.

Notably, our results collectively suggest a rather distributed arrangement of spatial representations across the RSC. First, while the univariate analysis failed to uncover noticeable differences between cue conditions in adaptation, the multivariate analysis revealed significant differences in voxel-to-voxel adaptation pattern between cue types. Such a scenario typically occurs when the neural representations are spatially distributed across the brain region. Second, the eight posterior portions of the RSC exhibited greater adaptation compared with its two anterior portions, yet the magnitude of adaptation across these eight posterior portions was very similar. This finding suggests that the neural representations were widely distributed throughout at least 80% of the RSC volume. Finally, no discernible differences were observed among the 10 portions in their contributions to the cue-specific multi-voxel adaptation patterns, indicating that even the two anterior portions showing weaker adaptation contributed equally to discriminating cue conditions as the eight posterior portions showing greater adaptation.

#### Control analyses

Compared with our previous study (Chen et al., 2019), one important advantage of the current paradigm is that it allowed us to evaluate whether the adaptation-based spatial coding was truly allocentric for each cue condition, by dissociating the length of the path leading to the test location and the allocentric position of the test location, since the starting position of the passive movement was randomized on a trial-by-trial basis for each cue type. In addition, as in our previous study, we also considered potential confounding influences of response adaptation, temporal distance, and passive movement. Given the preceding results showing that the adaptation in the RSC for landmark was predominately driven by location identity, we adopted the location-identity-based adaptation for landmarks to increase sensitivity of the following statistical analyses.

To preview, we found that spatial coding in the RSC for both cue types reflected allocentric positions of the test locations and was not confounded by the aforementioned factors. In contrast, for the spatial coding in the PHC for self-motion cues, allocentric location could not be unambiguously disentangled from path length and response.

##### Path length

To investigate whether the adaptation effects in the RSC reflected path length or allocentric positions of the test locations, we directly compared these two variables by including both the allocentric-location-defined and the path-length-defined parametric regressors in the same first-level GLM (Mumford et al., 2015). For example, if the participant traveled 10 m to Loc3 and 8 m to Loc 4 on two successive trials, then the path-length-defined parameter has a value of |10 − 8| = 2 m, whereas the location-defined parameter has a value of 4 m for self-motion cues and 1 (= different locations) for landmarks. In other words, path-length-based adaptation means that brain activation for the current trial is proportional to the absolute difference in path length between two successive trials.

In the RSC, the unique contribution of path length was not significant for either cue type (landmark, *t*_(19)_ = 1.185, *p*_1-tailed _= 0.125, BF_10_ = 0.430^j1^; self-motion, *t*_(19)_ = 1.374, *p*_1-tailed _= 0.093, BF_10_ = 0.525^j2^), after accounting for allocentric location. On the contrary, the unique contribution of allocentric location was significant for both cue types (landmark, *t*_(19)_ = 2.980, *p*_1-tailed _= 0.004, BF_10_ = 12.694^j3^; self-motion, *t*_(19)_ = 1.929, *p*_1-tailed _= 0.034, BF_10_ = 2.102^j4^), after accounting for path length.

In the PHC, in the self-motion condition, the unique contribution of allocentric location to adaptation was not significant after accounting for path length (*t*_(19)_ = 0.464; *p*_1-tailed _= 0.324; BF_10_ = 0.342^k1^), although the path-length-based adaptation was not significant itself (*t*_(19)_ = 1.183; *p*_1-tailed _= 0.126; BF_10_ = 0.739^k2^).

Given that the trial sequences were designed based on allocentric positions of the test locations to maximize the relative detection power in the first place (DP_rel_; Aguirre et al., 2011), we evaluated whether differential detection power could have led to the observed dominance of allocentric location in adaptation (Extended Data 1, Section 1.7). As expected, the relative detection power was significantly greater for allocentric location than for path length (*F*_(1,19)_ = 9.184; *p* < 0.001; $\eta_{p}^{2}$ = 0.326^l^), but the magnitude of the difference was minimal (mean DP_rel _= 64.2% vs 60.6%; mean difference, 3.6%). This suggests that the differential adaptation effects associated with allocentric location and path length are unlikely to have been caused by differences in detection power.

In brief, these results indicate that adaptation effects for both cue types in the RSC predominantly reflected neural coding for allocentric location and were not confounded by the path to the test location. In contrast, the adaptation effect in the PHC for self-motion cues could not be disentangled from path length.

##### Response adaptation

Although our task design ensured that choosing a given test location was not associated with any consistent motor behavior (Materials and Methods), it is important to examine whether the adaptation was confounded by possible response-related adaptation based on participants' subjective recognition of the test location. Trial-specific subjective response—reflecting a participant's belief of the currently occupied location—allowed for a trial-wise dissociation between objective location and subjective response. We directly compared location and response by including the location-defined parametric regressor and the response-defined parametric regressor in the same GLM1 (Mumford et al., 2015).

In the RSC, the unique contribution of response was not significant in either cue condition, after accounting for location (landmark: *t*_(19)_ = 0.117, *p*_1-tailed _= 0.454, BF_10_ = 0.254^m1^; self-motion: *t*_(19)_ = −0.781, *p*_1-tailed _= 0.778, BF_10_ = 0.143^m2^). In contrast, the unique contribution of location was significant in both cue conditions (landmark, *t*_(19)_ = 2.093, *p*_1-tailed _= 0.025, BF_10_ = 2.694^m3^; self-motion, *t*_(19)_ = 2.137, *p*_1-tailed _= 0.023, BF_10_ = 2.892^m4^), after accounting for response. These results indicate that objective location rather than subjective response was the driving factor of the adaptation effects in the RSC for both cue types.

In the PHC, in the self-motion condition, the unique contribution of location was not significant after accounting for response (*t*_(19)_ = 0.583; *p*_1-tailed _= 0.283; BF_10_ = 0.383^n1^), although the response-based adaptation was not significant itself (*t*_(19)_ = 1.118; *p*_1-tailed _= 0.139; BF_10_ = 0.683^n2^).

Although the location-defined trial sequences had significantly higher empirical DP_rel_ than the response-defined trial sequences, as expected (*F*_(1,19)_ = 19.007; *p* < 0.001; $\eta_{p}^{2}$ = 0.500^o^), the magnitude of the difference was negligible (mean DP_rel_ = 64.2% vs 63.1%). This indicates the differences in adaptation associated with location and response were not caused by differences in detection power.

Finally, we conducted the voxel-wise analysis to investigate adaptation in the entire volume (Extended Data 1, Fig. E7). In posterior cingulate areas (including RSC proper and the putative retrosplenial complex), adaptation appeared to be stronger when based on location than response in both cue conditions. This trend also existed in other brain regions, for example, precuneus, calcarine, and angular gyrus.

However, one potential limitation of this control analysis pertains to the high behavioral accuracy exhibited by our participants, particularly in the landmark condition. The dissociation between objective location and subjective response was positively proportional to the number of mistakes committed by participants. Nonetheless, we consider the results obtained from this analysis valid, for the following three reasons. First, the reliability of beta estimates for the GLM regressors is determined by the correlations between the parametric regressors modeling the location-based adaptation and the response-based adaptation, which turned out to be generally lower than the behavioral accuracy levels (Extended Data 1, Fig. E8). This discrepancy holds particularly true for the landmark condition, where the parametric regressors modeled location identity instead of continuous inter-location distance (Fig. E8*a*). Second, the robustness of beta estimates of the GLM regressors was bolstered by the large number of trials included in the current task design, which could mitigate adverse effects of multicollinearity to some extent. Third, the pattern of results was consistent across both cue types. Nevertheless, we acknowledge that future investigations employing more challenging tasks are needed to validate our findings.

##### Temporal distance

Neural adaptation is highly sensitive to the temporal intervals between successive events (Barron et al., 2016). In the paradigm used in our previous study (Chen et al., 2019), we demonstrated that adaptation effects in the EC were not confounded by the temporal delay between successive trials, because temporal delay and spatial relation were minimally correlated in the first-level GLM. Here, we conducted the same analysis to contrast these two variables by including the parametric modulation regressors defined by each of them in the same first-level GLMs (Mumford et al., 2015). For example, if the participant visited Loc1 and Loc2 in two successive trials, and the temporal delay between the two location occupation events was 8 s, then the temporal-distance-defined parameter had a value of 8, and the location-defined parameter had a value of 4 m for self-motion trials and 1 (= different locations) for landmark trials.

Same as in our previous study, the location-defined parameter and the temporal-distance-defined parameter had minimal correlations with each other ((|rs| < 0.1; ps > 0.1; *n* = 256). Furthermore, in the RSC, the unique contribution of location was significant after accounting for temporal distance for both cue types (landmark, *t*_(19)_ = 3.399, *p*_1-tailed_ = 0.002, BF_10_ = 28.206^p1^; self-motion, *t*_(19)_ = 3.082, *p*_1-tailed_ = 0.003, BF_10_ = 15.359^p2^). Conversely, after accounting for location, the unique contribution of temporal distance was not significant in the landmark condition (*t*_(19)_ = 1.171; *p*_1-tailed _= 0.128; BF_10_ = 0.728^p3^) and was significant in the self-motion condition (with one outlier winsorized, *t*_(19)_ = 2.044; *p*_1-tailed _= 0.028; BF_10_ = 2.488^p4^). However, unlike location, the unique contribution of temporal distance for self-motion cues was significant in all the other ROIs (*p*s_1-tailed _< 0.04; BFs_10_ > 2), except the right alEC (*t*_(19)_ = 1.287; *p*_1-tailed _= 0.107; BF_10_ = 0.841^p5^). Together, these results indicate that the positional coding observed in the RSC was not confounded by the temporal distance between successive location occupation events.

In the PHC, in the self-motion condition, the unique contribution of location was significant after accounting for temporal distance (*t*_(19)_ = 1.961; *p*_1-tailed _= 0.032; BF_10_ = 2.186^q1^), as was the unique contribution of temporal distance (*t*_(19)_ = 2.309; *p*_1-tailed _= 0.016; BF_10_ = 3.841^q2^).

In brief, all the adaptation effects observed in the RSC and PHC were not confounded by temporal distance between successive location occupation events, because our task design ensured that temporal distance and inter-location relation were largely dissociated and minimally correlated with each other. Interestingly, there existed neural coding of temporal distance for self-motion cues in most of our ROIs after accounting for location; however, the interpretation of this phenomenon was not pursued here.

##### Passive movement phase

In the location identification task, the location occupation phase—which was used to assess adaptation-based positional coding and was rendered the same for the two cue types—was preceded by a passive movement, during which the sensory inputs differed between the cue types. Given the slow temporal dynamics of the hemodynamic response, it is possible that the adaptation effect measured at the location occupation phase could have reflected effects in the passive movement stage.

To address this issue, in our main analyses, to account for potential influences of the passive movement phase that preceded the location occupation phase, the passive movement phase was modeled in the first-level GLMs (Materials and Methods). Here, we conducted additional analyses in which the passive movement phase was not modeled in the GLM and found that the adaptation results remained unchanged: the RSC showed significant adaptation for landmarks, *t*_(19)_ = 3.624, *p*_1-tailed _< 0.001, BF_10 _= 43.706^r1^; the RSC showed significant adaptation for self-motion cues, *t*_(19)_ = 3.026, *p*_1-tailed _= 0.003, BF_10 _= 13.818^r2^; the PHC showed significant adaptation for self-motion cues, *t*_(19)_ = 2.767, *p*_1-tailed _= 0.006, BF_10_ = 8.572^r3^; when comparing the voxel-to-voxel distributed pattern of adaptation between the cue types, the within-day adaptation distinction score was significantly >0 in the RSC (*t*_(29)_ = 2.694; *p*_1-tailed_ = 0.007; BF_10_ = 7.511^r4^), indicating cue-specific spatial representations in this region.

To thoroughly assess how preceding navigation experiences influence location-based adaptation during the location occupation phase, we created another GLM, in which we extended the regressors that represent the “movement” phase to encompass the entire navigation process, that is, including Phases 1, 2, and 3. Our primary findings remained stable with this revised GLM1. First, the univariate adaptation analyses showed that the RSC contained significant categorical location identity coding in the landmark condition (*t*_(19)_ = 3.462; *p*_1-tailed_ = 0.001; BF_10_ = 31.856^r5^) and continuous inter-location distance coding in the self-motion condition (*t*_(19)_ = 3.149; *p*_1-tailed_ = 0.003; BF_10_ = 17.435^r6^). The PHC showed significant adaptation in the self-motion condition (*t*_(19)_ = 2.836; *p*_1-tailed _= 0.005; BF_10_ = 9.724^r7^). Second, the multivariate analysis showed that in the RSC, the within-day adaptation pattern distinction score was significantly >0 (*t*_(19)_ = 2.508; *p*_1-tailed_ = 0.011; BF_10_ = 5.400^r8^), which was driven by the within-cue pattern similarity being >0 (*t*_(19)_ = 2.721; *p*_1-tailed_ = 0.007; BF_10_ = 7.880^r9^).

These results mean that the passive movement phase should not confound the adaptation-based positional coding assessed during the location occupation stage, as whether modeling the passive movement stage or not in the GLM did not affect the results. This is expected, because same as our previous study (Chen et al., 2019), the current task design ensured minimal correlation between the event regressor modeling the passive movement phase and the parametric regressor modeling the inter-location spatial relation for the location occupation phase in the first-level GLM (|rs| < 0.011). In brief, the adaptation effects observed in the RSC and PHC should not have been confounded by the passive movement phase preceding the location occupation phase.

#### Successful navigation involves brain regions different from our previous study (Chen et al. 2019)

So far, the current study exhibited a different pattern of results on adaptation from our previous study (Chen et al., 2019). To further illustrate potential differences between the two studies, we assessed whether the ROIs were involved in the navigation task by exhibiting the successful navigation effect (Pessoa et al., 2002; Chrastil et al., 2015), that is, stronger activation in correct trials than in incorrect trials (Materials and Methods).

**Figure 9.**
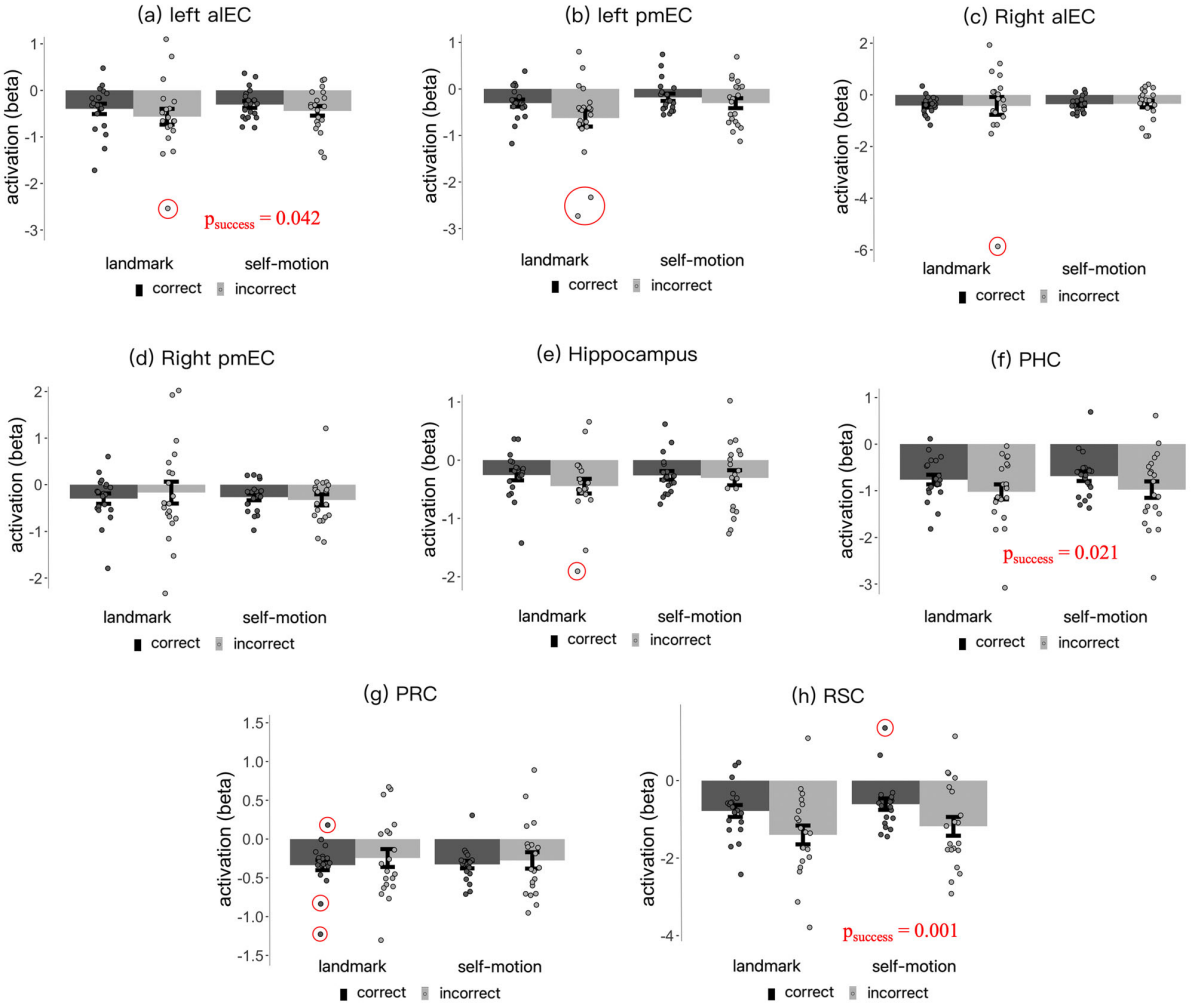
***a–h***, Effects of successful navigation in all ROIs. Brain activation is plotted as a function of cue type (landmark vs self-motion) and navigation success (correct vs incorrect). The left alEC, RSC, and PHC showed significant main effect of navigation success. Error bars represent ±SE.

As shown in Figure 9, the ROI-based analysis revealed significant successful navigation effects in the RSC (*F*_(1,19)_ = 17.267; *p* = 0.001; $\eta_{p}^{2}$ = 0.476^s1^), PHC (*F*_(1,19)_ = 6.282; *p* = 0.021; $\eta_{p}^{2}$ = 0.248^s2^) and left EC (with one outlier winsorized, *F*_(1,19)_ = 4.765; *p* = 0.042; $\eta_{p}^{2}$ = 0.201^s3^). These brain areas were more strongly activated in correct trials than in incorrect trials. Other ROIs did not show significant successful navigation effects (Fs < 1.5; *p* > 0.2; $\eta_{p}^{2}$ < 0.08). We did not observe any main effects of cue type (Fs < 3; *p* > 0.11; $\eta_{p}^{2}$ < 0.13) or interaction effects between cue type and correctness (Fs < 0.7; *p* > 0.4; $\eta_{p}^{2}$ < 0.04).

In brief, the results showed stronger recruitment of the RSC, PHC, and left alEC during successful trials compared to failed trials, underscoring their crucial role in facilitating accurate navigation. It is worth noting that different from the current study, our previous study (Chen et al., 2019) observed that all the MTL regions and the RSC exhibited significant successful navigation effect (Table E3). This discrepancy in successful navigation effect further indicates that dissimilar neural mechanisms were engaged in the navigation processes in our two studies.

## Discussion

The current study investigated whether landmark-based navigation and path integration recruit cue-specific or cue-independent spatial representations in the human brain, using a modified version of the simple navigation task employed in our previous investigation (Chen et al., 2019). The results showed the following: first, the RSC displayed adaptation-based positional coding for both landmarks and self-motion cues; second, the positional coding in the RSC primarily reflected allocentric spatial position rather than the path to the spatial location, indicating authentic allocentric spatial representations; and third, the voxel-to-voxel distributed pattern of adaptation differed between the cues, suggesting cue-specific positional coding. In addition to the RSC, we also observed positional coding for self-motion cues in the PHC, albeit smaller in magnitude and not dissociable from the path to the spatial location. Although we did not replicate our previous observation of adaptation-based positional coding in EC subregions under the current new experimental conditions, the brain regions contributing to successful navigation also differed from those observed in our previous study, indicating overall differential neural mechanisms between the two studies. In brief, to the best of our knowledge, the current study provides the first evidence for the existence of cue-specific spatial representations in the human RSC during the performance of a navigation task in the same spatial context. Furthermore, the current study underscores the dependency of spatial representations in the brain on specific experimental setups.

The following discussion will be organized as follows: first, we will discuss the positive findings of adaptation-based positional coding in the RSC and PHC. Second, we will analyze the key methodological differences between the current study and our previous study (Chen et al., 2019) to understand the discrepancies in the recruited neural mechanisms.

### Allocentric positional coding in the RSC

The central finding of the current study is the adaptation-based positional coding in the RSC for both cue types. This finding aligns with many studies in humans and nonhuman animals that have implicated the RSC in landmark-based navigation and path integration. For instance, human neuroimaging studies have demonstrated that the RSC encoded permanence of visual landmarks (Auger et al., 2015), that RSC activity was modulated by the navigator's instantaneous Euclidean distance from the path origin in a loop-closure path integration task (Chrastil et al., 2015), and that RSC activity encoded location identity for spatial locations defined by landmarks in real-world environments (Morgan et al., 2011). Similarly, studies in rodents have shown that firing patterns of retrosplenial place-cell–like neurons were influenced by both visual and self-motion information (Fischer et al., 2020; Mao et al., 2020) and that RSC lesions caused deficits in both path integration (Elduayen and Save, 2014) and landmark-based navigation (Clark et al., 2010). Collectively, these findings provide strong evidence that the RSC processes both landmark and self-motion information during spatial navigation (Todd and Bucci, 2015), which is consistent with the RSC's role as a polymodal midline structure receiving inputs from various (sub-)cortical areas that process different sensory inputs (Kobayashi and Amaral, 2003; Parvizi et al., 2006; Vann et al., 2009).

While broadly consistent with previous research, our study offers novel evidence that the RSC mediates spatial representations in both navigation modes in the same task. Furthermore, spatial coding in the RSC was not confounded by the length of the path taken by the participant to reach the location, meaning that it reflects a genuine positional code of allocentric locations. This finding aligns with the recent discoveries of place-cell–like neurons in the RSC, which, similar to hippocampal place cells, fire when a rodent occupies a specific location in the environment (Mao et al., 2017; Miller et al., 2021). Finally, the navigation task employed in the current study solely involved positional estimation on a one-dimensional linear track without requiring head direction estimation, emphasizing the RSC's role in positional processing without angular computations (Chrastil et al., 2016; Mao et al., 2017, 2020). This finding complements the well-documented role of the RSC in head direction representations (Taube, 1998; Marchette et al., 2014; Shine et al., 2016).

While the RSC exhibited positional coding for both landmarks and self-motion cues, our results showed different representational formats between them. For landmarks, positional coding primarily distinguished between the same and different locations, indicating neural coding of location identity. In contrast, the RSC's positional coding in the self-motion condition reflected inter-location distance in a continuous manner. Notably, the spatial distance information contained in RSC activity for self-motion cues was precise enough to allow for accurate recovery of the original physical space.

Why should the representational format differ between the two cue types? One possible explanation is that the underlying neuronal tuning curves associated with test locations were sharper for landmarks than for self-motion cues (Fang et al., 2007). This explanation is in line with our behavioral finding that participants performed better with landmarks than with self-motion cues, because sharper tuning curves typically lead to better discrimination among locations. When tuning curves for individual locations are very sharp, as hypothesized for landmarks, there is complete neural overlap when successive locations are the same. However, this overlap quickly reduces to minimal levels when the successive locations differ, resulting in location identity coding. In contrast, when tuning curves are broader, as hypothesized for self-motion cues, the neural overlap between successive locations decreases only gradually with increasing inter-location distance, resulting in inter-location distance coding.

The tuning curve explanation relates the adaptation effect in the RSC to participants' overt behavioral performance, which may appear contradictory to our finding that the adaptation effect was driven by the stimulus input (i.e., objective location) rather than the retrieved memory reflected in participants' behavior (i.e., subjective response). However, this contradiction is not necessarily valid, as stimulus input and behavioral performance are intertwined; for example, high-quality stimulus can facilitate memory retrieval. It is conceivable that different parameters of the presumed tuning curves contribute to different aspects of the adaptation effect and participants' behavior. The means of the tuning curves dictate their positions in space, determining whether objective location or subjective response fits the adaptation effect better. Meanwhile, the standard deviations of the tuning curves denote the precision of the sensory information, influencing the format and magnitude of adaptation, which, in turn, relates to behavioral accuracy and is likely impacted by memory stage.

Nevertheless, sharp tuning curves alone may not fully account for the location identity coding for landmarks in the RSC. First, previous fMRI adaptation studies have reported that with very high behavioral accuracy, the RSC can exhibit either continuous inter-location distance coding (Sulpizio et al., 2014; Kim and Maguire, 2018) or location identity coding (Morgan et al., 2011). Second, our previous study reported continuous inter-location distance coding for both landmarks and self-motion cues in the EC in terms of adaptation, when behavioral accuracy was higher for landmarks than for self-motion cues (Chen et al., 2019), albeit still considerably lower than the current study (Extended Data 1, Fig. E3). Finally, these two different formats of adaptation-based neural coding have been observed in different brain regions in the same experiment (Fang et al., 2007; Morgan et al., 2011). We speculate that besides behavioral accuracy, other factors such as intrinsic neuronal response properties of the brain region may influence the adaptation format. For instance, location identity coding in the RSC, which is probably driven by sharp tuning curves, might reflect the RSC's crucial role in the spatial processing of landmarks—the RSC might be particularly tuned to extracting precise spatial information from landmarks to support discrete landmark-based navigation (Yoder et al., 2011; Auger et al., 2012; Fischer et al., 2020). Moreover, our explicit instruction to retrieve the identities of individual test locations might have emphasized the extraction of discrete location identity information, at least for landmarks.

Furthermore, the tuning curve explanation should be taken with caution, given the complex relationship between fMRI adaptation and neuronal activity (Larsson et al., 2016). Further investigation is needed to fully elucidate the factors influencing the format of spatial representation in various brain regions.

In addition to the RSC, we also observed positional coding in the PHC for self-motion cues. This finding is consistent with previous research that has demonstrated the involvement of the PHC in path integration based on optic flow (Chrastil et al., 2015, 2016). However, unlike the RSC, we could not determine this coding in the PHC to be truly allocentric, since location adaptation was not dissociable from adaptation to path length.

### Positional coding in the RSC is cue-specific

Crucially, beyond showing that RSC activity contained allocentric positional information extracted from both landmarks and self-motion cues, our study also revealed the positional coding to be cue-specific rather than cue-independent, because the distributed pattern of adaptation was distinct between the two cue types. This finding suggests the existence of distinct underlying neural units that code spatial information for different cue types (Norman et al., 2006).

It is important to keep in mind that in our task, the sole difference between different cue conditions was the type of spatial information available. Therefore, cue-specific spatial representations in the RSC were not confounded by extrinsic factors such as task requirements or reward setups (Radvansky et al., 2021) but rather reflected the effects of spatial cues used for navigation. Furthermore, the cue-specific adaptation-based positional coding in the RSC was mainly driven by correlated distributed adaptation patterns within the same cue type, which indicates that the significant univariate adaptation effects in the RSC are unlikely to be false-positive results. This is because pure noise should be independent across different fMRI scanning runs, resulting in zero voxel-to-voxel correlations across runs (Walther et al., 2016).

Two related studies have investigated cue specificity of spatial representations in the RSC, even though studies alike abound on the HIPP (Markus et al., 1994; Geva-Sagiv et al., 2016; Radvansky et al., 2021). The first study reported that the place-cell–like population vector coding of spatial locations in the rodent RSC remained largely unchanged when the light was switched on versus off, indicating cue-independent spatial representations (Mao et al., 2017). The second study is a human fMRI study, which also reported cue-independent spatial representations in the RSC, because several readouts of brain activity (e.g. overall brain activity, inter-region functional connectivity pattern, multi-voxel similarity of brain activation) did not differ between different virtual environments learned with different degrees of body-based self-motion cues (Huffman and Ekstrom, 2019).

How can we reconcile the current finding of cue-specific spatial representations with these two studies? First, a clear dissociation of the cues, as demonstrated in the cue-specific behavioral accuracy profiles across test locations in the current study, may be critical for inducing cue-specific representations. Specifically, we observed that in the landmark condition, behavioral performance improved as the test location approached the landmark, whereas the opposite pattern emerged in the self-motion condition. This cue-specific behavioral performance pattern can be attributed to the fact that spatial precision of landmark-based navigation diminishes as the distance from the landmark increases (Chamizo et al., 2006; Chen et al., 2019), whereas path integration becomes more prone to noise as the navigator traverses the path away from its anchoring point—the starting position of movement (Stangl et al., 2020). These findings align with previous research demonstrating a relative independence of path integration and landmark-based navigation in behavioral studies (Etienne et al., 1996; Chen et al., 2017), indicating that our cue dissociation manipulation successfully elicited distinct navigational strategies with different spatial cues.

In this context, it is important to note that in the study by Mao et al. (2017), self-motion cues were present both when the light was on and off, along with other possible uncontrolled cues (e.g., somatosensory and olfactory cues). Therefore, the observed cue-independent spatial representations may have rather reflected the coding of certain common spatial information shared by different cue conditions.

Second, experiencing the sensory information to retrieve locations from memory might be another key to evoking cue-specific representations. Unlike our on-line navigation task in which participants actually navigated with sensory information available within the virtual environment, Huffman and Ekstrom (2019) asked participants to imagine themselves occupying previously learned locations, without perceiving the sensory inputs that obviously differed between different cues.

Finally, we utilized fMRI adaptation to evaluate positional coding. It is worth noting that fMRI adaptation often exhibits heightened sensitivity to the low-level physical characteristics of stimuli, as opposed to measures such as the representational similarity of activation vectors or the multi-voxel pattern analysis in general (Epstein et al., 2003; Epstein and Morgan, 2012; O’Connell et al., 2018). This observation aligns with our discovery of cue-specific location-based adaptation in the RSC, because the sensory inputs evidently differed between cue types at a level lower than abstract positional representations. One plausible explanation for the stimulus sensitivity of fMRI adaptation is that it might reflect the synaptic processing (Sawamura et al., 2006; Epstein and Morgan, 2012). However, the elusive relationship between BOLD signals and neuronal activity (Arthurs and Boniface, 2002), combined with the finding that BOLD signals generally correlate better with synaptic inputs than spiking outputs of neurons (Logothetis et al., 2001), leaves the precise reason for stimulus sensitivity of fMRI adaptation unclear. Nevertheless, it is conceivable that fMRI adaptation interrogates different aspects of the underlying neural codes compared with other measures, such as univariate mass activation and multi-voxel activation pattern similarity in the study of Huffman and Ekstrom (2019) and neuronal spiking activity in the study of Mao et al. (2017). These methodological differences may explain the disparities between the current study and the two prior studies, highlighting the importance of employing complementary measurements to obtain a more complete picture of how the brain processes various spatial cues during navigation (Chen et al., 2022).

### Distinct neural mechanisms between the current study and our previous study (Chen et al. 2019)

Despite employing similar task paradigms and identical MRI scanning sequences to contrast landmarks and self-motion cues in spatial navigation, the current study yielded divergent fMRI results from our previous investigation in terms of both adaptation and successful navigation effects (Chen et al., 2019). Regarding adaptation, while our previous study observed adaptation in the right EC, the present study only revealed adaptation in the RSC and PHC, but no adaptation in the EC. The absence of significant adaptation in the EC could not be explained by signal quality, as the temporal signal-to-noise ratio in the EC was actually lower in our previous study (≅11.3) than in the current study (≅13.5; Fig. E10). Regarding successful navigation, the overall participation of MTL was substantially reduced in the current study, whereas the participation of the RSC remained comparable between the two studies.

These discrepancies can be attributed to two notable methodological differences between our two studies. First, compared with our previous study (Chen et al. 2019), participants in the current study were at a relatively late memory stage during functional scanning: participants initially showed learning effects on the Pre-scan_day, reaching a performance plateau that remained stable throughout the two sessions of functional scanning (Fig. E2). This finding was likely due to two factors: first, participants were trained intensively on a separate day prior to the scanning; and second, participants' spatial memories probably had been consolidated by sleep between the days. In contrast, participants in our previous study did not exhibit any learning effects, indicating they were fixed at an early memory stage (Fig. E3). There is abundant evidence indicating that memories transition from MTL to cortical areas over time (Alvarez and Squire, 1994). Prior spatial navigation studies have also demonstrated a shift in the locus of spatial representations from the HIPP to RSC over time, as hippocampal activity reflects the learning rate, whereas the retrosplenial activity is associated with the fidelity of acquired spatial knowledge (Wolbers and Büchel, 2005; Diersch et al., 2021). Therefore, our findings of reduced involvement of MTL in navigation task completion and strong spatial representations in the RSC might be attributable to the relatively late memory stage.

The second critical difference was the longer temporal intervals between location occupation events in the current study compared with our previous study (Chen et al. 2019; mean interval≅7.2 s vs ≅3 s). This difference stemmed from the wider spacing of test locations in the current study, while movement speeds remained unchanged. fMRI adaptation is highly sensitive to temporal intervals; the longer ones may attenuate neuronal adaptation (Barron et al., 2016). However, since varying temporal intervals should not affect participants' behavioral performance and hence the involvement of the brain regions in accurate navigation, temporal intervals are unlikely to be the sole factor contributing to the discrepancies between our two studies.

In summary, our previous observation of adaptation-based spatial coding in the entorhinal subregions but not in the RSC and other MTL regions cannot be generalized to the current experimental context, highlighting the dynamic and complex nature of spatial information processing in the brain. Future studies are warranted to elucidate the factors influencing neural coding during different navigation modes.

### Limited involvement of the HIPP

We observed little hippocampal involvement in the positional coding. While we identified one cluster showing adaptation to landmarks (Fig. E4, a.2) and one cluster showing successful navigation effect extending into the HIPP (Fig. E9*c*), their peak voxels did not reside within the HIPP according to AAL template denotation. These results echo with our previous study, where a cluster showing adaptation to self-motion cues extended into the HIPP, but its peak voxel fell within the thalamus (Chen et al., 2019; Fig. 5).

Interestingly, although the univariate analysis did not reveal adaptation for either cue type in the HIPP (Fig. 4*b*), the multivariate analysis revealed cue-specific adaptation patterns in this region (Fig. E12). Additionally, the HIPP showed strong functional connectivity with the RSC (Fig. E13). These results implicate the potential existence of weak positional coding in the HIPP (Norman et al., 2006).

In summary, with only one spatial cue type for navigation, the HIPP appears to exhibit limited positional coding. This finding aligns with rodent studies indicating that robust positional coding in the HIPP requires the integration of different spatial cues (Brun et al., 2008; Lu et al., 2013).

### Conclusions

Our study aimed to address a fundamental question in spatial navigation: Do landmark-based navigation and path integration utilize shared or separate spatial representations in the brain? We tested the generalizability of our previous finding of spatial coding in the right EC under a different experimental condition. Although not replicating this previous finding in the new paradigm, we discovered strong and cue-specific allocentric spatial representations in the RSC. Further investigations should clarify the elements that impact neural encoding of spatial information during different navigation modes.

## Synthesis

Reviewing Editor: Ifat Levy, Yale School of Medicine Decisions are customarily a result of the Reviewing Editor and the peer reviewers coming together and discussing their recommendations until a consensus is reached. When revisions are invited, a fact-based synthesis statement explaining their decision and outlining what is needed to prepare a revision will be listed below. The following reviewer(s) agreed to reveal their identity: Li Zheng.

This paper expands previous research from the same group (Chen et al 2019). The authors have previously reported that different sub-regions of enthorhinal cortex (EC) encode position information for landmark and self-motion cues. Here, using univariate adaptation analysis, the previous results in EC were not replicated, but the authors report location information in the retrosplenial cortex (RSC). Within this area, activation patterns were cue-specific, indicating separate neural mechanisms for landmark-based and self-motion-based navigation.

Overall, this is an interesting extension of the 2019 study. The aim of this study is clear, the experiment is well-designed with thorough controls for potential confounds, and the manuscript is well written. The main results remain consistent in both ROI-based and voxel-wise analyses and offer additional evidence to enhance our understanding of neural mechanisms during navigation. However, reviewers had some concerns regarding the methodology and the interpretation of the results which should be addressed.

- Could the authors provide more details about GLM1? In the model, they have included phase 4 and the movement phase as regressors, but what about the other phases, such as phases 1, 3, 5, and 6? Are they also treated as separate regressors, or are they simply left together with the null event as the default baseline?

- Regarding the GLM used for "fMRI analysis of successful navigation", did it utilize the same parametric analysis employed in the adaptation analysis, or was it solely based on the univariate activation analysis, with a focus on distinguishing between correct and incorrect trials? Given that the authors compared these results to their 2019 study, is this the same GLM that the author used in that study?

- In the ROI analysis, the authors found location-identity coding in the landmark condition and more gradual distance effect in the self-motion condition. Are landmark and self-motion cues encoded in anatomically separated parts of RSC? Although the authors conducted voxel-based analysis and reported significant clusters in RSC (page 15), the main text does not provide information on the location of these clusters. A main figure overlaying the clusters from the landmark and self-motion conditions (similar to Fig. 5 in the 2019 paper) would be very helpful.

- In addition, an appropriate way to compare the cue-specificity of adaptation effect is to contrast the self-motion adaptation map and cue-specificity adaptation map (a simple contrast in univariate analysis) for the differences, and conjunction analysis for the similarities.

- The authors conducted a new analysis that mixes multivoxel pattern similarity analysis (MVPA) with a univariate adaptation effect. They measured whether the multivoxel adaptation pattern was consistent for each cue type across scanning blocks. Reviewers were concerned about this analysis. Imagine a simple scenario where voxel A shows strong adaptation for distance in (self-motion, nature 1st) and (self-motion, nature 2nd) runs, and zero adaptation for all landmark runs. In contrast, voxel B shows near zero adaptation to distance in both self-motion and landmark runs. Then the pattern distinction score would be positive, and the authors conclude that "we observed distinct distributed adaptation patterns between landmarks and self-motion cues in RSC... This effect occurred within the same scanning session, and was regardless of the environment in which these cues were experienced." (page 19, line 25). However, in this case the adaptation effect for landmark is clustered around voxel A, rather than distributed. We also cannot tell whether the same voxel shows the same level of adaptation for both nature and city environment, because the authors compared the adaptation patterns within nature environments (Fig 6c) or within city environments (Fig 6d), but not explicitly between nature and city.

- Why didn't the authors use a conventional MVPA approach to test whether neural responses are more similar between the same location regardless of cue. E.g. (location1 in Landmark) is more similar to (location1 in Self-motion) than to (location 2-4 in Landmark or Self-motion).

- In the landmark condition, RSC showed a distinct pattern of location without a linear distance effect, and the authors cited the location-repetition effect in Morgan et al. (2011). Another study, however, showed a more gradual distance effect in the right RSC, but not in the left RSC. In Kim & Maguire (2018, Cortex, Fig 5B), right RSC showed higher activity when participants successively visited a diagonal room than a vertical room (distance was longer for diagonal than vertical condition). Was there a difference in left and right RSC in the current dataset?

- As the authors mention, sharper tuning curves could be the reason for independent neural representations without gradual distance effect. They note the higher accuracy in the landmark condition as supporting different tuning curves. However, in their previous study, both the landmark and self-motion conditions showed gradual distance effect in EC and accuracy was again higher in the landmark condition. Behavior accuracy doesn't seem like a deciding factor for the format of representation.

- Unlike in Chen et al. (2019), here a unique color was associated with each location and participants probably imagined the color. Is it possible that because of this, participants encoded the locations in a more categorical manner, resulting in the location identity code, instead of gradual distance code (at least for the landmark condition)?

- Based on the control analysis of response adaptation, the authors posited that in the RSC, the driving factor behind the adaptation effects for both cue types was objective location rather than subjective response, because after controlling for the subjective response, the unique contribution of location was still significant. If this is the case, does it imply that the observed adaptation effects are not related to memory but rather to the perception of position during online navigation? In other words, does this suggest that irrespective of participants' ability to recall positions from memory, the RSC may automatically encode positional information as long as they focus on perceiving task-provided cues? The question arose because when the authors attempted to explain why the current study did not replicate their previous study from 2019, they asserted that "participants in the current study were in a relatively late memory stage during MRI scanning." As a result, it is possible that memories had already transferred from the MTL to cortical areas (i.e., RSC here). This is a plausible interpretation, but it appears to conflict with their conclusion that "RSC codes were driven by objective location rather than subjective response". Also, given the high behavioral accuracy, shouldn't the objective location and subjective location (response) be highly correlated?

- Relatedly, how was the performance in Chen et al. (2019)? Did participants show a learning effect? Please include performance information either in the main or supplementary material, allowing the comparison of two experiments.

- In Chen et al. (2019), cue reliability was manipulated, 2 trees vs. 1 tree. They found the distance coding in EC only in the high reliability condition. Is there a reason why only 1 tree is included in the current experiment? Could it be a reason for the absence of distance coding in EC?

- Fig S3, a.2 shows a cluster in hippocampus, not parahippocampal gyrus. A.3 MNI coordinate of (-22,-42,-5) lies between the lingual gyrus and the posterior hippocampus. Thus, the hippocampus seems to contain the location information, at least in the landmark condition, in line with many previous studies.

- Also, hippocampal voxels are visible in Fig S5 c (correct > incorrect contrast). Did they not survive the multiple comparison correction?

- Univariate adaptation analysis suggests that "RSC shows strong evidence for positional coding for both landmarks and self-motion cues." Reviewers wondered whether there was any evidence from multivariate RSA analysis that supports positional coding in RSC, such as higher within-position pattern similarity compared to between-position pattern similarity? Perhaps the authors could explore this by utilizing the beta-values used in the neural space reconstruction analysis?

- In page 27, the authors argue that fMRI adaptation might reflect the synaptic inputs, more so than firing rate, overall brain activation, or connectivity (the measures in previous literatures). RSC is indeed known for integrating multiple inputs at dendritic levels (Voigts & Harnett 2019 Neuron) and sensory inputs are different between the cue types. But it is important to note that not only adaptation, but the BOLD hemodynamic signal in general is a better correlate of inputs than spiking output. Various differences in experimental paradigms may be a more likely explanation for the differences in RSC finding, rather than fMRI adaptation vs. overall BOLD activity/RSA.

Minor points

-page 4, line22, 'refer to video' ->please add the link or hyperlink to the video here

-page 14, line2-7: what does s stand for in 'ts', 'ps'?

-page 25, line 11: Morgan et al. (2012) should be Morgan et al. (2011)

-supplementary material shows tSNR across two days and ROIs. tSNR was higher on 2nd day than 1st day. What could be the reason for this?

-supple Fig1 legend, " as depicted in (c.1)", what is (c.1)? Does it refer to the graphical illustration (panel a in Fig.S1)?

-supple. Fig1 D, can you please clarify the observed value and simulated value in panel (d)? Are these 4x4 values from confusion matrix of 4 location? But the data points seem much fewer than 16.

-Comparing detection power in control analysis was good.

## Author Response

Synthesis of Reviews:

Computational Neuroscience Model Code Accessibility Comments for Author (Required):

N/A Synthesis Statement for Author (Required):

The authors are praised for conducting a thorough revision and additional analyses. The manuscript is now stronger and the core outcomes and interpretations are very well argued.

Our response: We appreciate the positive feedback on our revised manuscript and are pleased that the reviewers are satisfied with our revisions. We are grateful for the reviewers' valuable comments, which have helped us improve the manuscript.

Comment (1) Reviewers said:

In their response the authors mention a preprint reporting RSA analysis - it would be very useful if they cite the preprint in the discussion of the current manuscript.

Our response:

We appreciate the reviewers' suggestion. Now we have added the suggested citation to our discussion on the potential use of different fMRI measurements to enhance our understanding of spatial representations in the brain. On P 40, L 7-11, we now state: "These methodological differences may explain the disparities between the current study and the two prior studies, highlighting the importance of employing complementary measurements to obtain a more complete picture of how the brain processes various spatial cues during navigation (Chen et al., 2022). " Comment (2) Reviewers said:

A minor point: Fig E8 legend: "ranked by behavioural accuracy" should be "ranked by regressor correlation" Our response:

We apologize for the oversight. This mistake has now been corrected in the updated manuscript
