# Supplementary material for: Repetition Suppression Reveals Cue-Specific Spatial Representations for Landmarks and Self-Motion Cues in the Human Retrosplenial Cortex
Source: eNeuro. 2024 Apr 8;11(4):ENEURO.0294-23.2024. doi: 10.1523/ENEURO.0294-23.2024 (PMC11007318; doi:10.1523/ENEURO.0294-23.2024)
Supplement: Extended Data 1 — Download Extended Data 1, DOCX file. [file eneuro-11-ENEURO.0294-23.2024-s003.docx]

Extended Data 1

1. Supplemental methods

*1.1. Learning task*

The learning task helped participants learn the four test locations. Participants used an MRI-compatible joystick to navigate around in the virtual environments and give responses. First, participants were shown the four test locations that were evenly spaced on the linear track (Figure 1a). Four balls of different color were positioned at the four test locations. Participants needed to remember the colors of the balls associated with the test locations. Next, only one of the four balls were shown, and the participant needed to ‘move’ to the position of the ball by pressing a button on the joystick. (see the video – the part “LEARNING”). Throughout the learning task, both the landmark and self-motion information were available.

*1.2. Behavioral training (Pre-scan day)*

The behavioral training allowed the participants to get familiar with the virtual reality environment and to learn the four test locations. The training consisted of three parts. Each part had a learning stage and a test stage. During the learning stage participants completed the aforementioned learning task (Section 1.1) (see the video – the part “LEARNING”). First, they learned the colors of the balls positioned at the four test locations and were tested on their memory of the colors. Next, they learned the locations of the four balls. In each trial, one ball was displayed, and the participant actively moved from a randomized starting position to the ball’s location. Both the landmark and self-motion cues were available, meaning the arrows, the tree, and the ground texture of the linear track were all visible. Each ball was learned twice, with the order of the four balls counterbalanced. The learning stage was performed twice for each environment, with the order of the two environments counterbalanced. The learning stage was identical for all the three parts in the Pre-scan day. During the test stage, the participant performed the ‘location identification task’ as described in the preceding section (Figure 1b; also see the video – the part “TEST: location identification task”). There were four blocks in total (counterbalanced), corresponding to the four combinations of environment (city vs. nature) and cue condition (self-motion vs. landmark).

In the first part, each block had four trials, corresponding to the four ball locations (counterbalanced). In the second and the third parts, during the test stage, each block had 16 trials, with four trials for each ball location (counterbalanced). The experimenter carefully instructed the participants from the beginning to the end during the first part of the Pre-scan training. For the remaining two parts of the training, participants were left alone to perform the tasks, but were attended by the experimenter when needed.

*1.3. Permutation-based multiple comparison correction for t tests*

To correct for multiple comparisons, we adopted the maximum-t-statistic approach and performed the permutation-based one sample direction t tests. First, in each permutation, every entry in each measurement was randomly multiplied by -1 or +1, and the t statistic was calculated for the permuted data of each measurement. Next, the maximum t statistic was obtained out of all the measurements. After 5000 permutations, we obtained a surrogate distribution of maximum t statistic, to which we compared the observed t statistic calculated from the actual data in each measurement. The significance level (i.e., p_corrected_) equaled to the proportion of values in the surrogate distribution of maximum t statistic that were greater than the observed t statistic.

*1.4. Group-level anatomical mask for small volume correction in voxel-wise analysis*

We created a group-level anatomical mask encompassing bilateral MTL and RSC. First, participant-specific anatomical masks of all ROI were combined. Then, the participant-specific combined mask was normalized to the MNI template. Next, the participant-specific normalized masks were averaged across participants. The mean anatomical mask therefore consisted of values greater than 0 and no greater than 1. Finally, the mean mask was thresholded by a value of > 0, so that a given voxel was included in the group-level anatomical mask if it was included in the combined mask of at least one participant.

*1.5. Adaptation-based neural space reconstruction analysis*

To evaluate whether the fMRI adaptation effect contains fine-grained distance information between different test locations, we adapted the neural space reconstruction analysis to fMRI adaptation (Marchette et al., 2014; Peer & Epstein, 2021; Persichetti & Dilks, 2019).

This analysis consisted of three steps (Figure 5c). In Step 1, we constructed the neural distance matrix, whose elements denote pairwise neural distances between the test locations. The neural distance between two test locations could be quantified as the brain region’s activation level for one location when preceded by the other location - the lower the region’s activation to the current location when preceded by the other location, the larger the repetition suppression effect, the closer the two test locations would be positioned to each other in the neural space. We constructed a GLM, which modeled the location occupation phase in individual single trials with separate regressors. To construct the neural distance matrix, these trials were classified into 4**×**4 = 16 groups based on the combination of two locations visited in succession; the beta estimates for trials from the same group were averaged, resulting in the 4×4 neural distance matrix. In the matrix, rows represent the previous location, columns represent the current location, and each element represents the activation level at the current location when preceded by the previous location.

To keep it consistent with the main fMRI adaptation analysis, the neural space reconstruction analysis was restricted to trials that could be modeled for adaptation (i.e., locations not preceded by the null event and not the first event in the sequence). Nevertheless, to confirm that the baseline activation level was comparable for the four test locations in our ROIs, we estimated activation levels for the four test locations using the trials that were not included in the parametric regressors modeling fMRI adaptation (i.e., test locations preceded by the null event and the first event in the sequence). We observed no significant differences among the four locations in either RSC (p = 0.531), or the hippocampus (p = 0.459), or any other ROIs in the medial temporal lobe (ps > 0.1). This verifies our choice of using the estimated brain activation level for the current location as an indicator of the neural distance between the current location and the preceding location.

In step 2, we normalized the neural distance matrix to render all the elements within the range [0, 1]. Elements that were diagonally symmetrical to each other in the matrix were averaged.

In step 3, the normalized neural distance matrix was subjected to multidimensional scaling and the Procrustes analysis. The multi-dimensional scaling recovers the spatial coordinates of the locations in the neural space, following the basic principle that locations with greater representational similarity are positioned closer to each other in the neural space (Kruskal & Wish, 1978). Subsequently, the Procrustes analysis mapped the estimated coordinates of the locations to the original physical space through rotations and reflections (Gower & Dijksterhuis, 2004).

To assess whether the neural space resembled the original physical space, we adopted a nonparametric permutation-based test. The normalized neural distance matrix was averaged across participants to obtain the grand group-level neural distance matrix for the four test locations (Marchette et al., 2014; Peer & Epstein, 2021; Persichetti & Dilks, 2019), which was then subjected to multidimensional scaling and the Procrustes analysis. The nonparametric permutation test was conducted as follows. First, we obtained the actual Procrustes distance calculated from the group-level neural distance matrix, which indicates the deviation of the reconstructed neural space from the original physical space. Second, we applied the permutation procedure to obtain the surrogate distribution of Procrustes distance, to which the actual Procrustes distance would be compared. Specifically, in each permutation, we randomly shuffled the entries in the grand group-level neural distance matrix. Note that to allow for more permutations, this shuffling was done prior to the averaging of symmetrical off-diagonal elements in the neural distance matrix. We obtained the Procrustes distance by applying multidimensional scaling and the Procrustes analysis to the shuffled neural distance matrix. This process was repeated 5000 times, resulting in a surrogate distribution of Procrustes distance. Third, the actual Procrustes distance was compared to the surrogate distribution. The significance level (i.e., p value) was calculated as the proportion of values in the surrogate distribution being smaller than the actual Procrustes distance, analogous to directional one-sample t test. Significant results would indicate that the group-level neural space resembled the original physical space.

*1.6. Connectopic mapping in the retrosplenial cortex*

*Data pre-processing in the current study*. The analyses were performed on the native (unnormalized) residual functional data after extraction of task-related activity using GLM1 on the data acquired on MRI_day1 (see ‘Methods’ section for details). Mean time series of CSF and white matter were modeled as regressors of no interest in the GLM. Outlier scans were identified using the SPMUP toolbox in terms of both framewise displacement and global intensity (https://github.com/CPernet/spmup). Each of the outlier scans was modeled with a separate spike regressor in the GLM. The residual fMRI data served as a proxy of the resting-state brain activity. Residual functional data underwent band-pass filtering (0.01–0.1 Hz).

*Data acquisition and pre-processing in a separate dataset*.

A high-resolution whole-brain T1-weighted structural scan was acquired with the following MP-RAGE sequence: TR = 2500 ms, TE 1 = 3.55 ms, TE 2 = 5.00 ms, flip angle = 7º, slices = 192, orientation = sagittal, resolution = 1 mm isotropic. The functional resting-state scans were acquired with a T2*-weighted 2D echo planar image slab centered on the hippocampus and parallel to its long axis (TR = 2200 ms, TE 1 = 13 ms, TE 2 = 38 ms, flip angle = 15º, slices = 80, orientation = sagittal, resolution = 2 mm isotropic, echo spacing = 0.68 ms). A total of 200 functional scans were obtained, and the total acquisition time was about 6 minutes 30 seconds. The first 5 functional scans were discarded, resulting in a total of 195 funcitonal scans in the data analysis. At the same time, a high-resolution T2-weighted structural scan (0.4*0.4*1.6 mm) was also acquired, which was not used in the current data analysis.

The functional resting-state data was de-noised using the FIX artifact removal procedure implemented in FSL 6.0. We first trained a multi-level classifier using manually labeled data from 20 participants. The classifier was then applied to automatically identify and remove noise components in the remaining subjects' data. The clean processed data was subsequently registered to MNI template for further analysis.

*Data-driven connectivity analysis.* We utilized ConGrads (Haak et al., 2018), a toolbox enabling data-driven exploration of functional connectivity changes within a specified brain region. The fMRI time series from the retrosplenial cortex (RSC) and gray-matter voxels outside the RSC were transformed into matrices, with dimensionality reduction applied to the latter using SVD. Voxel-wise connectivity fingerprints within the RSC were determined through correlation with SVD-transformed data, and similarities were quantified using the η2 coefficient. The LE algorithm processed the similarity matrix, producing vectors representing dominant modes of connectivity change. In the current study, participant-specific results were obtained by running the LE algorithm on the participant-specific similarity matrices. In the separate functional resting-state dataset acquired at a 3T scanner, these computations were carried out at the group-level. The left and right hemispheric RSC regions were analyzed separately. Topology preservation was assessed using Spearman’s rank correlation coefficient, emphasizing the LE algorithm's intrinsic focus on preserving connectivity order. Correlation coefficients are reported without p-values due to the algorithm's emphasis on maximizing topology preservation.

*1.7. Analysis of empirical relative detection power of fMRI adaptation*

In the current study, we used the eight de Bruijn sequences from Chen et al. (2019). These sequences were generated based on objective locations, and hence, were theoretically optimized in terms of ${DP}_{rel}$ with respect to objective locations. However, participants’ responses or trial-wise path lengths could not be known in advance, leading to the possibility that ${DP}_{rel}$ was reduced for the subjective-response-defined or path-length-defined sequences compared to the objective-location-defined sequences.

To address this issue, we calculated the empirical ${DP}_{rel}$ for objective-location-defined, subjective-response-defined, and path-length-defined sequences separately, based on the first-level general linear models (GLMs). These GLMs included regular regressors modeling the location occupation events as a measure of the direct stimulus effect, and a parametric regressor modeling objective location, or subjective response, or path length as a measure of the adaptation effect.

Specifically, ${DP}_{rel}$is calculated as follows (Aguirre et al., 2011),

${DP}_{rel}= \frac{{var}_{0}}{{var}_{1}}$

in which ${var}_{0}$ represents the hypothesized neural modulation after HRF convolution and high-pass filtering (e.g., f > 1/128), and ${var}_{1}$ represents the original hypothesized neural modulation prior to HRF convolution and high-pass filtering.

To calculate ${var}_{1}$, we constructed these first-level GLMs with no HRF convolution and no high-pass filtering applied. We then converted the simulated BOLD signal of the parametric regressor from the time domain to the frequency domain, using the fast Fourier transform (FFT). The variance of the hypothesized neural modulation for the parametric regressor (i.e., ${var}_{1}$) was calculated as the area under curve (AUC) using the frequency-domain data.

To calculate ${var}_{0}$, we convolved the predicted fMRI time series for the parametric regressor with the canonical hemodynamic response function (HRF), and adopted a high-pass filter with a cut-off at 1/128s = 0.0078 Hz. The variance of the convolved and filtered signal for the parametric regressor (i.e., ${var}_{0}$) was calculated in the same way as var_1_. Finally, to obtain ${DP}_{rel}$, we divided ${var}_{0}$ over ${var}_{1}$.

2. Additional results

*2.1. Cognitive modeling to recover representational precision from behavioral performance*

To dissociate representational precision from response bias and attentional failure in behavioral performance, we applied an extension of signal detection theory to our location identification task with four choices. In the modeling, we included eight free parameters to model i) the four standard deviations of the underlying representations of the four test locations (*S_1_, S_2_, S_3_, S_4_*), ii) the three response criteria (*C_12_, C_23_, C_34_*), and iii) the lapse rate ($lr$). The lapse rate represents the proportion of trials in which participants completely failed in attention and simply chose a response randomly (Zhang & Luck, 2008). The centers of the representation distributions (*μ_1_, μ_2_, μ_3_, μ_4_*) were assumed to be at the true positions of the test locations (i.e., *μ_1_* = -6m, *μ_2_ =* -2m, *μ_3_ =* 2m, and *μ_4_ =* 6m).

In each simulation, we constructed a behavioral confusion matrix, given a set of algorithm-generated values for the eight free parameters. Specifically, for the $1-lr$ proportion of the trials, we randomly sampled a sensory input $x$ from the normal distribution of the underlying representation corresponding to the test location presented in that trial, *N(μ_r_, S_r_)*. Then, a response $R(x)$ was made by comparing the sensory input $x$ to the three response criterions:

$$R(x)=\left\{ \begin{aligned} Loc1, x<C_{12} \\ Loc2, &C_{12}\ll x <C_{23} \\ Loc3,C_{23}\ll x<C_{34} \\ Loc4, x >C_{34} \end{aligned} \right.$$

For the remaining $lr$ proportion of the trials, we randomly selected one of the four choices as the response, regardless of the current sensory input.

In each simulation, we simulated 10000 trials for each of the four test locations to construct the 4x4 predicted behavioral confusion matrix. We normalized the predicted confusion matrix so that elements in the matrix ranged from 0 to 1, each representing the probability of a response falling to a certain cell of the matrix, e.g., $P_{r,c}$represents the probability of location $r$ recognized as location $c$. We then compared the actual behavioral confusion matrix to the predicted confusion matrix, by computing the probability of observing each actual response given the predicted confusion matrix. Finally, we summed the log-transformed probabilities of all actual responses,

$$\sum_{n=1}^{N} {log(P}_{r,c})$$

in which n represents the trial number and N represents the total number of trials in the actual experiment. We repeated the simulation to maximize this summed log-transformed probability (i.e., maximum likelihood estimation). We used the Hooke & Jeeves hill-climbing algorithm for model optimization (Hooke & Jeeves, 1961), as implemented in Matlab_R2020a. To avoid the potential local-minima problem, the model-fitting procedure was repeated 20 times with randomized starting values for the parameters each time, and the parameter estimates with the best fit were selected.

We performed bootstrapping to estimate variabilities of the estimates for these free parameters. In each iteration, we randomly sampled the same number of responses from the actual responses with replacement for each test location. We then submitted the sampled data to the abovementioned model fitting procedure, and obtained the estimates for the free parameters. The procedure was repeated 1000 times, resulting in distributions for all the eight free parameters. 95% confidence intervals of these estimates were obtained from these bootstrapped distributions.

To evaluate how well the model fitted the data, we simulated the behavioral confusion matrix, using the best-fitting values of the eight parameters. We simulated 1000 trials for each test location. We then calculated Pearson correlation between the simulated confusion matrix with the actual confusion matrix. R-squared was taken as a measurement of goodness-of-fit of the model, i.e., the proportion of variance in the data explained by the model. Because the numbers of correct vs. incorrect trials differed substantially, we evaluated the model fit separately for correct and incorrect trials, as well as separately for the landmark condition and the self-motion condition.

Results are displayed in Figure S1.


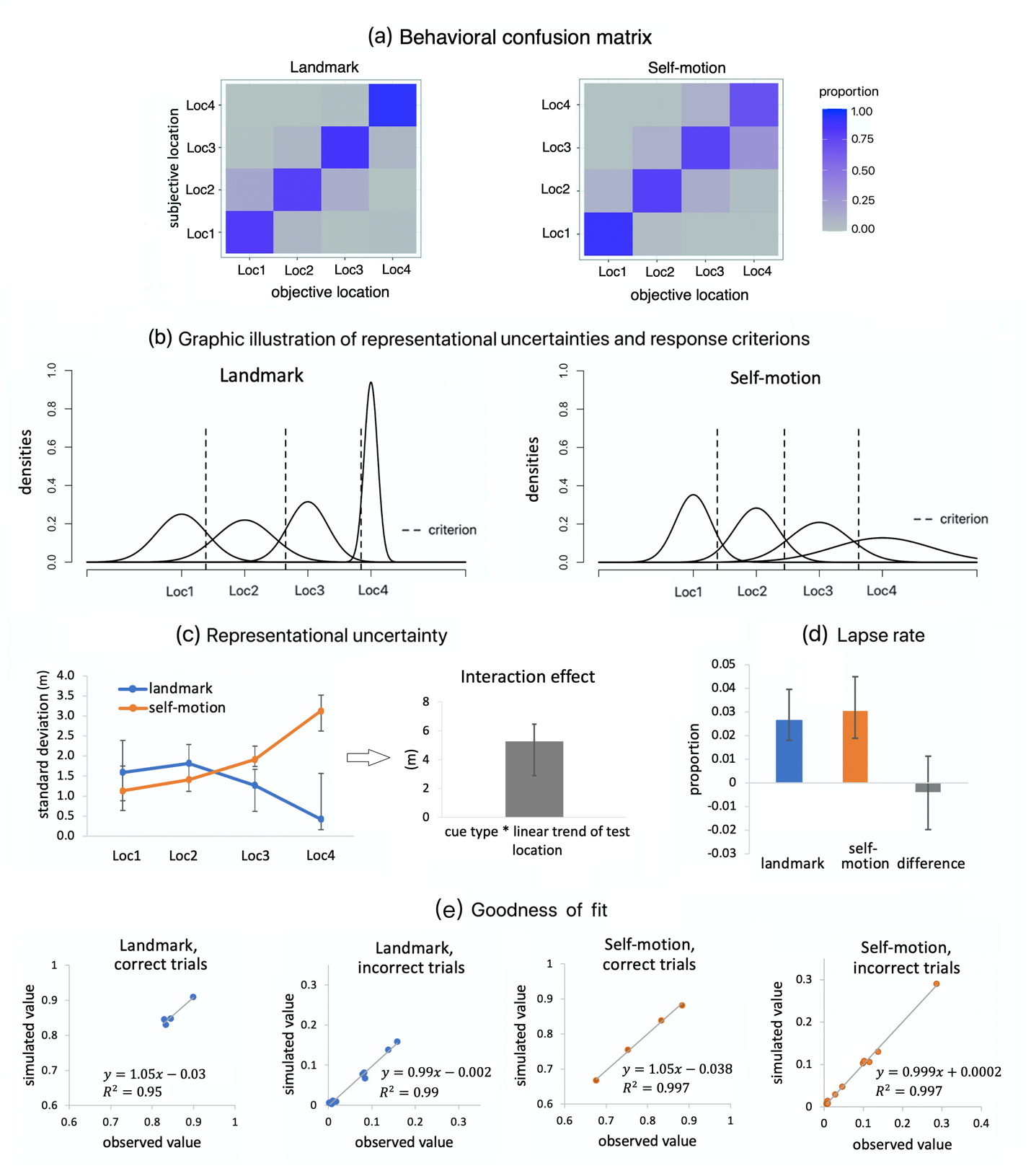


**Figure E1. Disentangling representational precision and response bias via modeling.**

1. Behavioral confusion matrix. Columns represent the actual test location occupied by participants (objective location), and rows represent the location participants thought they occupied (subjective location). Cell color represents frequency, i.e., the proportion of trials falling into the cell.
2. Graphic illustration of the estimated underlying representations and response criteria.
3. Estimated representational uncertainty (i.e., standard deviations of the Gaussian distributions in (b)) is plotted as a function of location and cue type. Error bars represent 95% confidence intervals obtained through a bootstrapping procedure. We further found that the interaction effect between cue type and the linear trend of test location on representational uncertainty was significant (i.e., the 95% confidence interval of the interaction effect did not contain zero).
4. Lapse rate was significantly higher than zero in both the landmark condition and the self-motion condition (i.e., the 95% confidence intervals did not contain zero), indicating that participants failed to pay adequate attention to the task occasionally. The difference in lapse rate was not significant between the two cue types. Error bars represent 95% confidence intervals obtained through a bootstrapping procedure.
5. Goodness-of-fit of the model. Regarding the behavioral confusion matrices, the observed values in the observed matrices are plotted against the simulated values generated by the model using the optimal values of the parameters, separately for landmarks and self-motion cues, and separately for correct and incorrect trials. Correct trials correspond to the four diagonal elements of each behavioral matrix in (a), and incorrect trials correspond to the 12 off-diagonal elements of each behavioral matrix in (a). For incorrect trials, some data points overlap with one another due to their very close values, which renders the plot appear to have fewer than 12 data points. The linear regression line, R^2^ (i.e., goodness-of-fit), and the regression equation are displayed in each scatterplot.

*2.2. Behavioral performance first improved, and then reached plateau prior to MRI scanning*

To evaluate potential learning effects in the current study, we submitted behavioral accuracy of all three days to a repeated-measures ANOVA, with day (Pre-scan_day vs. MRI_day1 vs. MRI_day2) and cue type (landmark vs. self-motion), and run (4 runs) as independent variables. As shown in Figure S2, The main effect of day was significant (F(2,38) =13.697 p < 0.001, η_p_^2^ = 0.419). Post-hoc tests showed that the two MRI scanning sessions did not differ from each other (p_holm_ = 0.306), whereas the two scanning sessions had significantly higher accuracy than the pre-scan day (ps_holm_ = 0.001). This indicates that while participants’ performance improved on the first scanning session compared to the pre-scan day, their performance stayed unchanged during the two scanning sessions. Main effect of cue type was significant (p < 0.001). Furthermore, the interaction between cue and day was not significant (p = 0.476), meaning the performance change pattern was similar for both cue types. No effects involving run were significant (ps > 0.1), meaning that behavioral performance remained relatively stable across time within each day. Other effects were not significant (p > 0.1).

We noticed that while behavioral accuracy seemed to increase linearly across runs in the Pre-scan day, it remained unchanged in the two scanning days. Therefore, within the omnibus ANOVA test, we calculated a contrast that compared the linear trend of run between the pre-scan day and the two scanning days combined (specification of the contrast coefficients: Pre-scan_day, run1*(-3) + run2*(-1) + run3*1 + run4*3; each scanning day, run1*1.5 + run2*0.5 + run3*(-0.5) + run4*(-1.5)), which yielded significant interaction (t(114) = 2.432, p = 0.017). Specially, the linear trend was significant in Pre-scan_day (t(170) = 3.518, p < 0.001), but not MRI_day1 (t(170) = 0.359, p = 0.720) or MRI_day2 (t(170) = 0.659, p = 0.511). This indicates that while the behavioral performance improved within the Pre-scan_day, the behavioral performance remained stable within each scanning day.

To further localize the performance improvement in time, we compared the last run of the Pre-scan_day and the first run of the first scanning day, with cue type as the second independent variable. There were no significant differences between these two runs (F(1,19) = 1.109, p = 0.306, η_p_^2^ = 0.055), and no significant interaction between run and cue type (F(1,19) = 0.573, p = 0.458, η_p_^2^ = 0.029). This indicates that no performance change occurred during the interval between Pre-scan_day and MRI_day1. Similar results were obtained when comparing the last run of the first scanning session and the first run of the second scanning session, indicating no performance change occurred during the interval between MRI_day1 and MRI_day2.

Together, these results showed that participants’ behavioral performance first improved, and then reached a plateau that remained stable over the course of the two scanning sessions. This stability is evident as performance improvements were observed exclusively during the Pre-scan day.


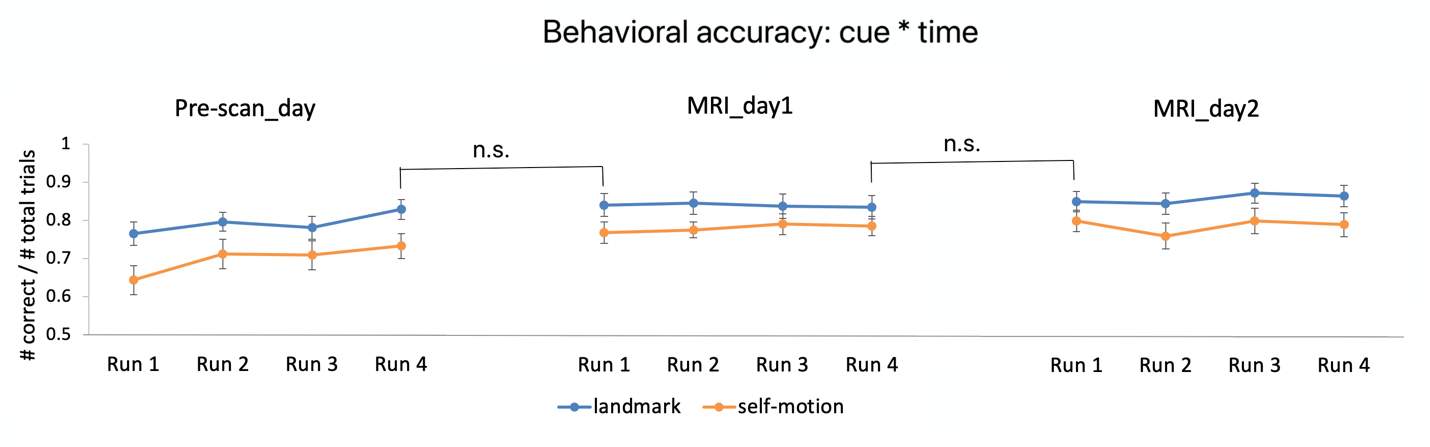


**Figure E2. Behavioral performance change across time.**

Next, to systematically compare participants’ performance change across time between the current study and our previous study (Chen et al., 2019), we conceived three different stages in each of the study: training, 1^st^ part of functional scanning (scan_p1), and 2^nd^ part of functional scanning (scan_p2). In the previous study, training refers to the practice during the structural scan prior to the functional scan; scan_p1 and scan_p2 refer to the 1^st^ run and 2^nd^ run of functional scanning, respectively. In the current study, training refers to the training in the Pre-scan_day; scan_p1 and scan_p2 refer to MRI_day1 and MRI_day2, respectively.

To account for different behavioral accuracy baselines between the studies, we computed “corrected behavioral accuracy”, which equaled to (accuracy – baseline)/(1- baseline); the baseline was 0.5 in our previous study and 0.25 in the current study. In the previous study, corrected behavioral accuracy was averaged across the high reliability condition and the low reliability condition for each cue type. We then submitted corrected behavioral accuracy to a repeated-measures ANOVA, with study, stage, and cue type as independent variables.

As shown in Figure S3, we observed a significant interaction between study and stage, F(2, 80) = 5.066, p = 0.008, $\eta_{p}^{2}$= 0.112. Following-up analysis showed that in the previous study, behavioral accuracy remained stable over time (F(2,42) = 0.115, p = 0.892, $\eta_{p}^{2}$= 0.005), meaning no learning effect occurred. On the contrary, as already described in the original manuscript, in the current study, behavioral accuracy improved over time (F(2, 38) = 14.824, p < 0.001, partial = 0.438), meaning learning effect occurred. Post-hoc comparisons showed that scan_p1 and scan_p2 were significant higher in accuracy than training (ts > 4, ps < 0.001), whereas scan_p1 and scan_p2 did not differ from each other (t = 0.843, p = 0.405).

These results indicate that in our previous study participants were fixed at the early memory stage during functional scanning, because no learning occurred. On the contrary, in the current study participants first showed learning effects in the Pre-scan day, then their behavioral performance reached plateau and remained stable throughout the two sessions of functional scanning, indicating that they were at a relatively late memory stage during functional scanning.

Another finding is that, we observed that the behavioral performance was significantly better in the current study than the previous study during the functional scanning. This is expected given that i) participants performed better in the current study at the training stage already, because the test locations were easier to discriminate perceptually in the current study participants had been extensively trained one day prior to the functional scanning, and ii) participants showed learning effect in the current study.


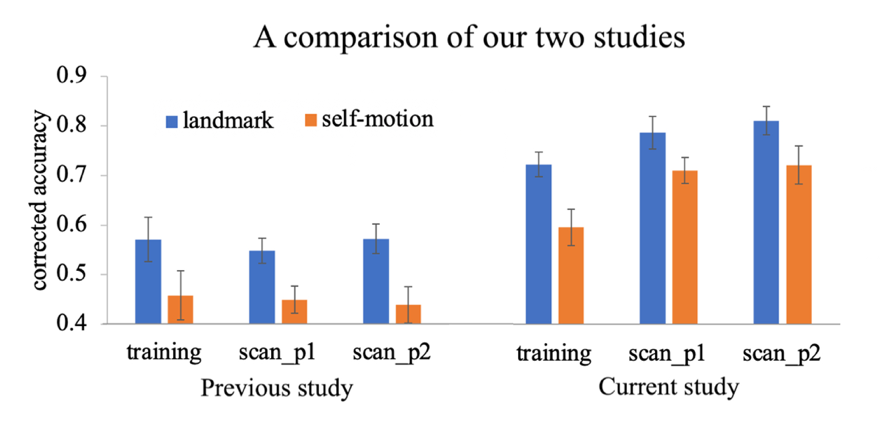


**Figure E3. Comparing performance change over time between our two studies.**

Corrected accuracy = (accuracy – baseline)/(1- baseline), in which baseline is equal to 0.5 in our previous study and 0.25 in the current study. In the previous study, training refers to the practice during the structural scan prior to the functional scan; scan_p1 and scan_p2 refer to the 1^st^ run and 2^nd^ run of functional scanning, respectively. In the current study, training refers to the training in the Pre-scan_day; scan_p1 and scan_p2 refer to MRI_day1 and MRI_day2, respectively. In our previous study, Behavioral accuracy was averaged across the high reliability condition and the low reliability condition for each cue type.

*2.3. Voxel-wise analysis of adaptation*

**
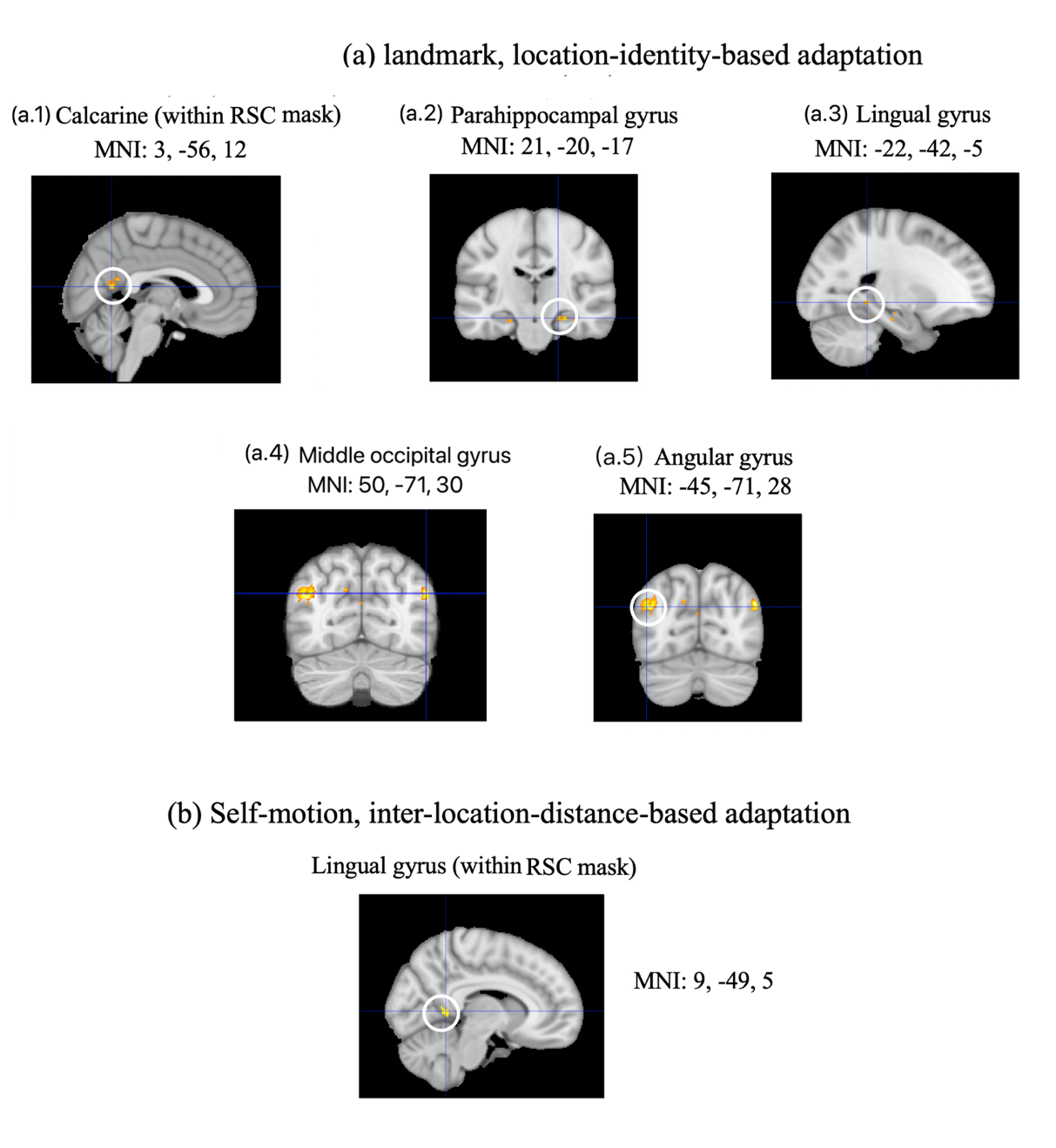
**

**Figure E4. Results of voxel-wise analysis of fMRI adaptation.**

The multiple comparison correction was performed using the non-parametric permutation-based test. The cluster-level inference approach was adopted. The group-level mask that encompassed all of our ROIs (i.e., bilateral medial temporal lobe plus RSC) was used for small-volume-correction. The results were displayed on the MNI template, thresholded at T > 3.

**Table E1. Voxel-wise analysis of adaptation with cluster inference.**

Multiple comparisons were corrected either within the group-level anatomical mask consisting of all ROIs, or across the entire search volume, using nonparametric voxel-inference (Nichols & Holmes, 2002). Cluster size was determined at voxel-wise T > 3. In correspondence to Figure S4.

| Brain Region | MNI Coordinate | | Voxel level (T) | Cluster size (k) | p_1-tailed_ (cluster-level) |
| --- | --- | --- | --- | --- | --- |
|  | RH | LH |  |  |  |
| *Landmark condition, location-identity-based adaptation* | | | | | |
| *MTL+RSC mask for small volume correction* | | | | | |
| Calcarine (within RSC mask) | 3, -56, 12 |  | 6.76 | 1029 | < 0.001 |
| Calcarine (within RSC mask) |  | -12, -46, 7 | 5.84 |  |  |
| Precuneus (within RSC mask) | 4, -50, 18 |  | 4.34 |  |  |
| Parahippocampal gyrus | 21, -20, -17 |  | 4.92 | 122 | 0.036 |
| Lingual gyrus |  | -22, -42, -5 | 4.83 | 135 | 0.028 |
| Fusiform gyrus |  | -23, -39, -17 | 4.10 |  |  |
| Parahippocampal gyrus |  | -28, -35, -11 | 3.55 |  |  |
| *Multiple comparisons correction across the entire search volume* | | | | | |
| Occipital_Mid_R | 50, -71, 30 |  | 6.84 | 556 | 0.043 |
| Occipital_Mid_R | 40, -79, 35 |  | 5.68 |  |  |
| Occipital_Mid_R | 45, -77, 27 |  | 4.15 |  |  |
| Calcarine (within RSC mask) | 3, -56, 12 |  | 6.76 | 4085 | 0.002 |
| Calcarine (within RSC mask) |  | -12, -46, 7 | 5.84 |  |  |
| Precuneus | 1, -63, 24 |  | 5.69 |  |  |
| Angular gyrus |  | -45, -71, 28 | 6.31 | 2388 | 0.004 |
| Middle occipital gyrus |  | -34, -84, 33 | 4.59 |  |  |
| Middle temporal gyrus |  | -41, -58, 18 | 4.51 |  |  |
| *Self-motion condition, inter-location-distance-based adaptation* | | | | | |
| *MTL+RSC mask for small volume correction* | | | | | |
| Lingual gyrus (within RSC mask) | 9, -49, 5 |  | 4.98 | 153 | 0.015 |
| *Multiple comparisons correction across the entire search volume* | | | | | |
| No significant voxels |  |  |  |  |  |

*2.4. Comparing the two hemispheres in ROI-based analysis of adaptation*


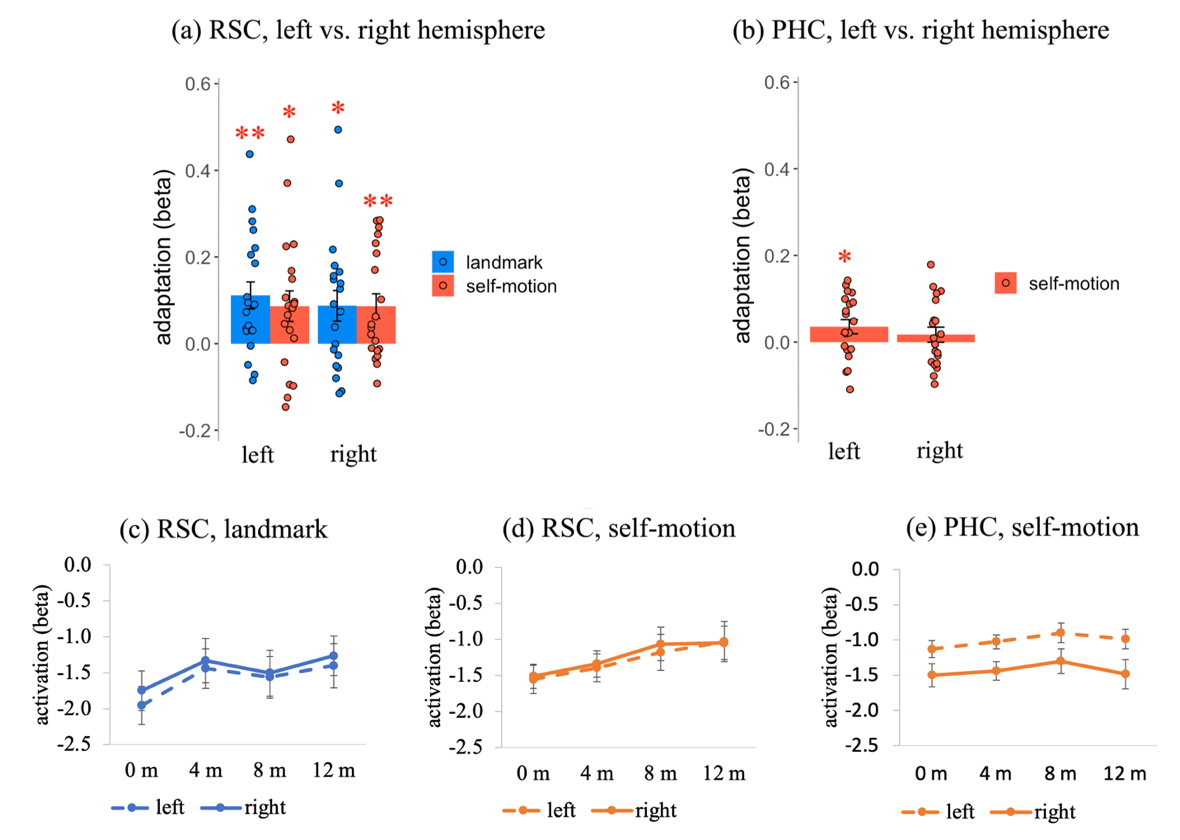


**Figure E5. Comparing two hemispheres in location-based adaptation**

Note: location-identity-based adaptation for landmarks and continuous-inter-location-distance-based adaptation for self-motion cues in (a) and (b). No differences were observed between the two hemispheres in any conditions (ps_2-tailed_ > 0.45).

*2.5. Additional results on functional-connectivity-based segmentation of RSC*


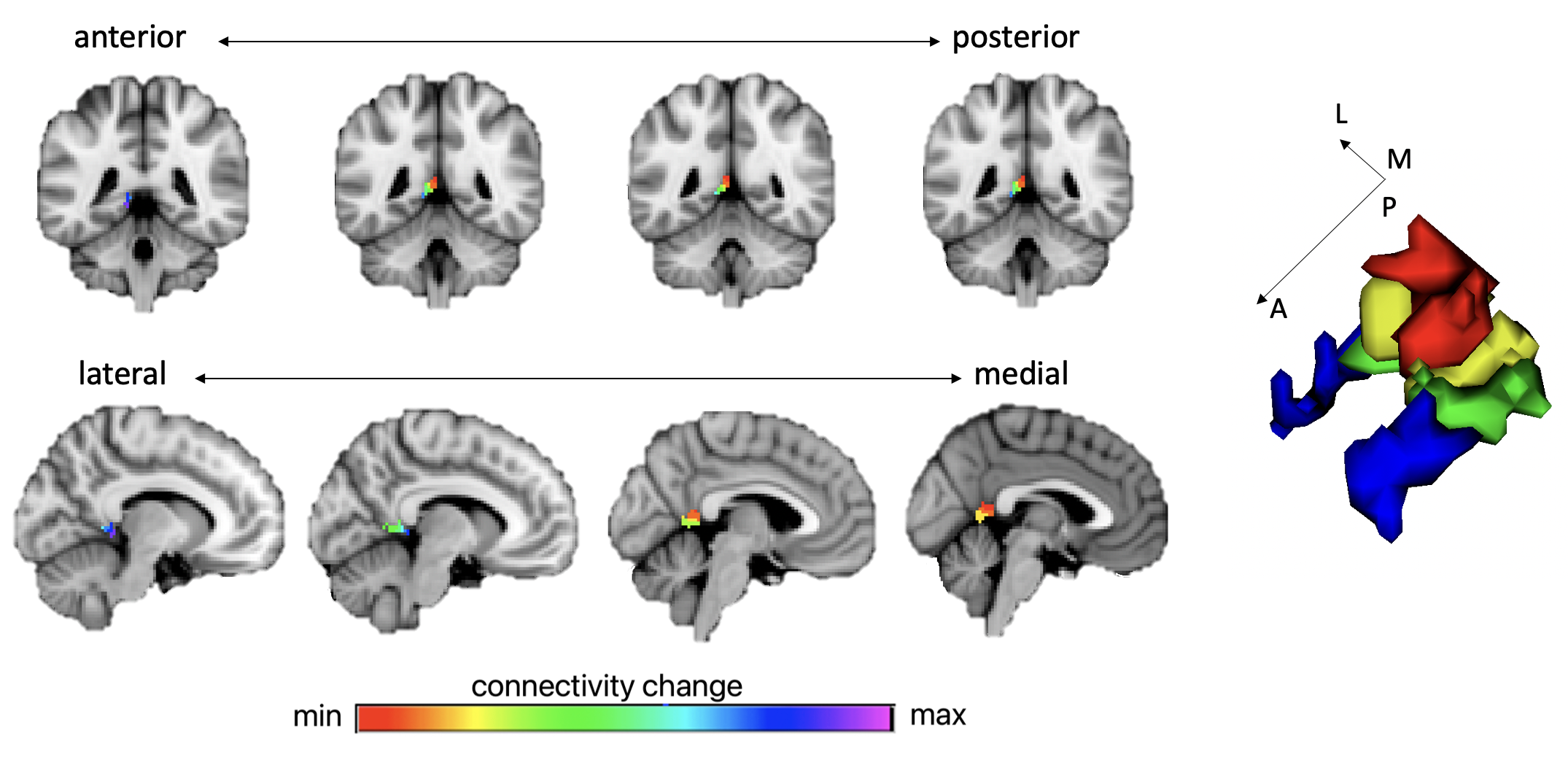


**Figure E6. Functional segmentation of RSC in an independent dataset acquired at 3T fMRI scanner.**

The left panel represents a visualization of the gradient in RSC based on the dominant connectopy. In the right panel, RSC was segmented into four equally-sized portions instead of ten portions, due to the lower spatial resolution in this dataset (i.e., 2 mm isotropic). The resting-state fMRI dataset was acquired in a separate study at a 3T scanner (Siemens, MAGNETOM Prisma). This analysis was conducted at the group-level in the MNI template, meaning that the RSC structure shown here was defined by the group-level anatomical mask of RSC.

*2.6. Comparing location and response in adaptation in the voxel-wise analysis*


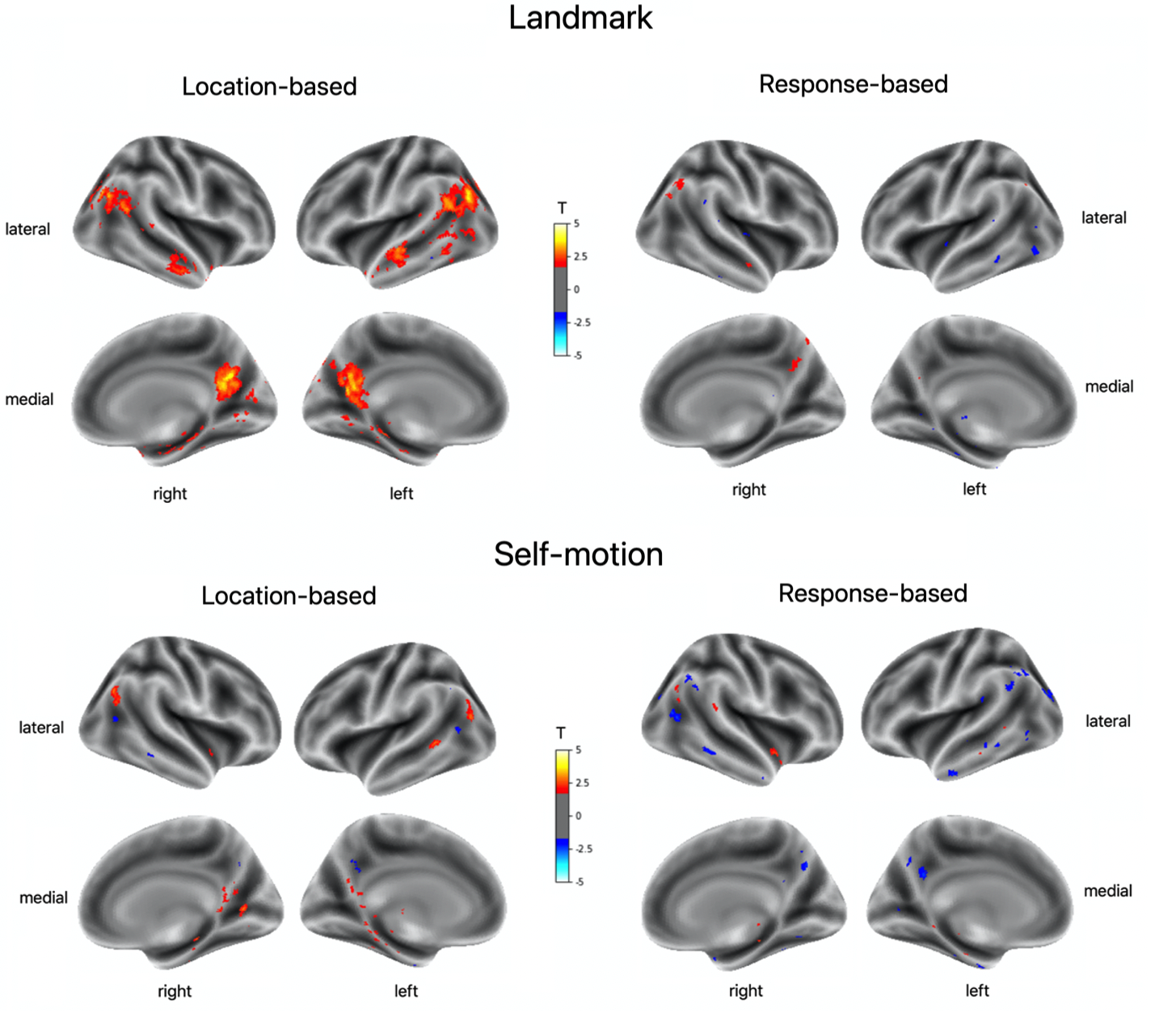


**Figure E7. Comparing location and response in the voxel-wise analysis of adaptation.**

Results are displayed for the landmark condition (upper) and the self-motion condition (lower), objective-location-based adaptation (left) and subjective-location-based adaptation (right). The parametric regressors modeled location identity (i.e., same vs. different locations) in the landmark condition, and inter-location distance in the self-motion condition. The participant-specific maps of adaptation were normalized to the MNI template and spatially smoothed with 3mm isotropic FWHM. For the 2^nd^ level analysis, we conducted directional one-sample t test against 0. The parametric t maps were overlaid on the MNI template and projected to the brain surface. Here, results are thresholded at p_uncorrected_ < 0.05.

*2.7. Correlations between parametric modulation regressors defined by objective location and subjective response.*

*
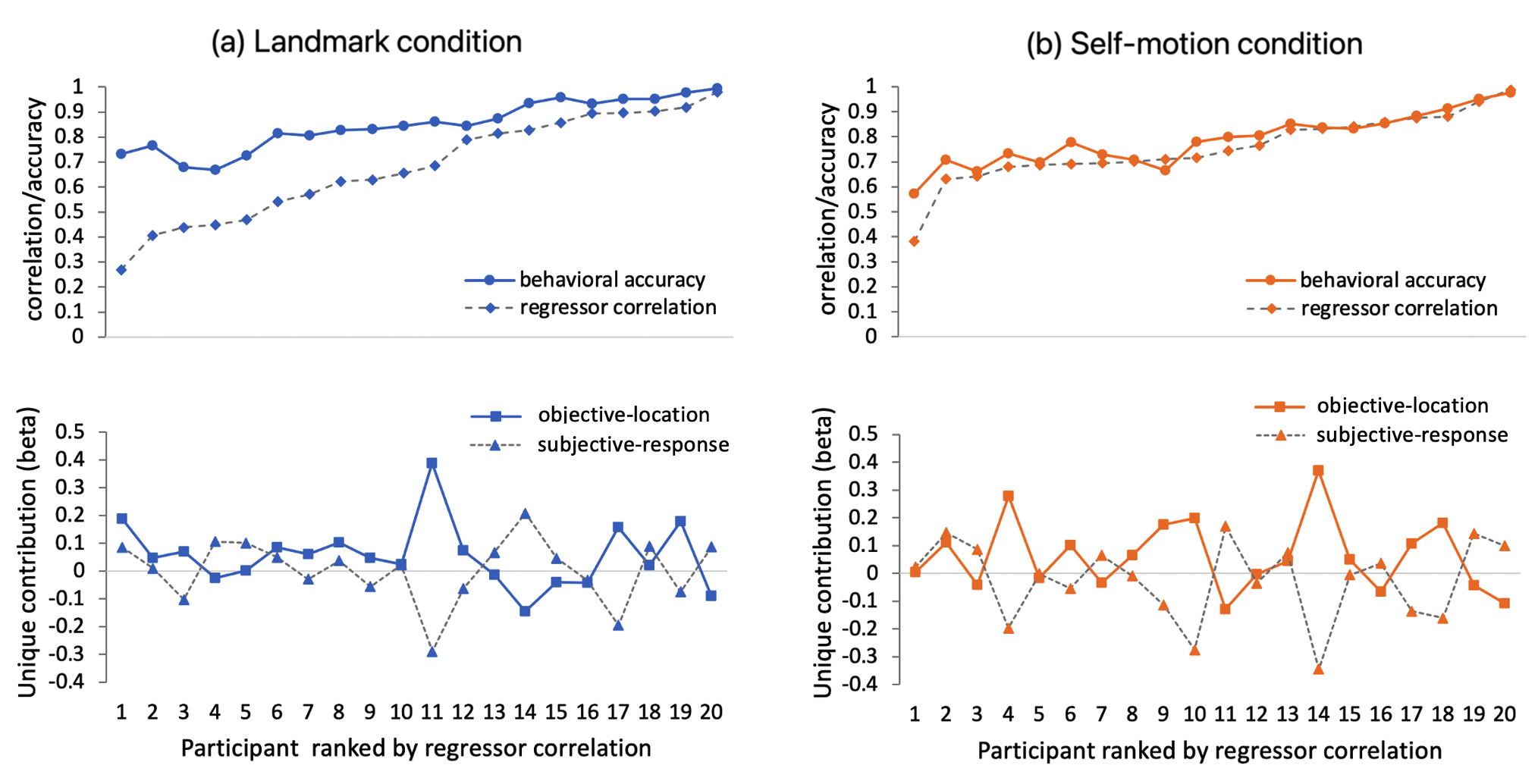
*

**Figure E8. Correlations between parametric modulation regressors defined by objective location and subjective response.**

(a) depicts results for the landmark condition, and (b) depicts results for the self-motion condition. Upper panels, behavioral accuracy and the Pearson correlation between the parametric modulation regressors defined by objective location and subjective response in the first-level GLM. Participants are ranked from left to right by regressor correlation. Lower panels plot the unique contributions of objective location and subjective response, with participants also ranked by regressor correlation.

*2.8. Analysis of successful navigation effect*


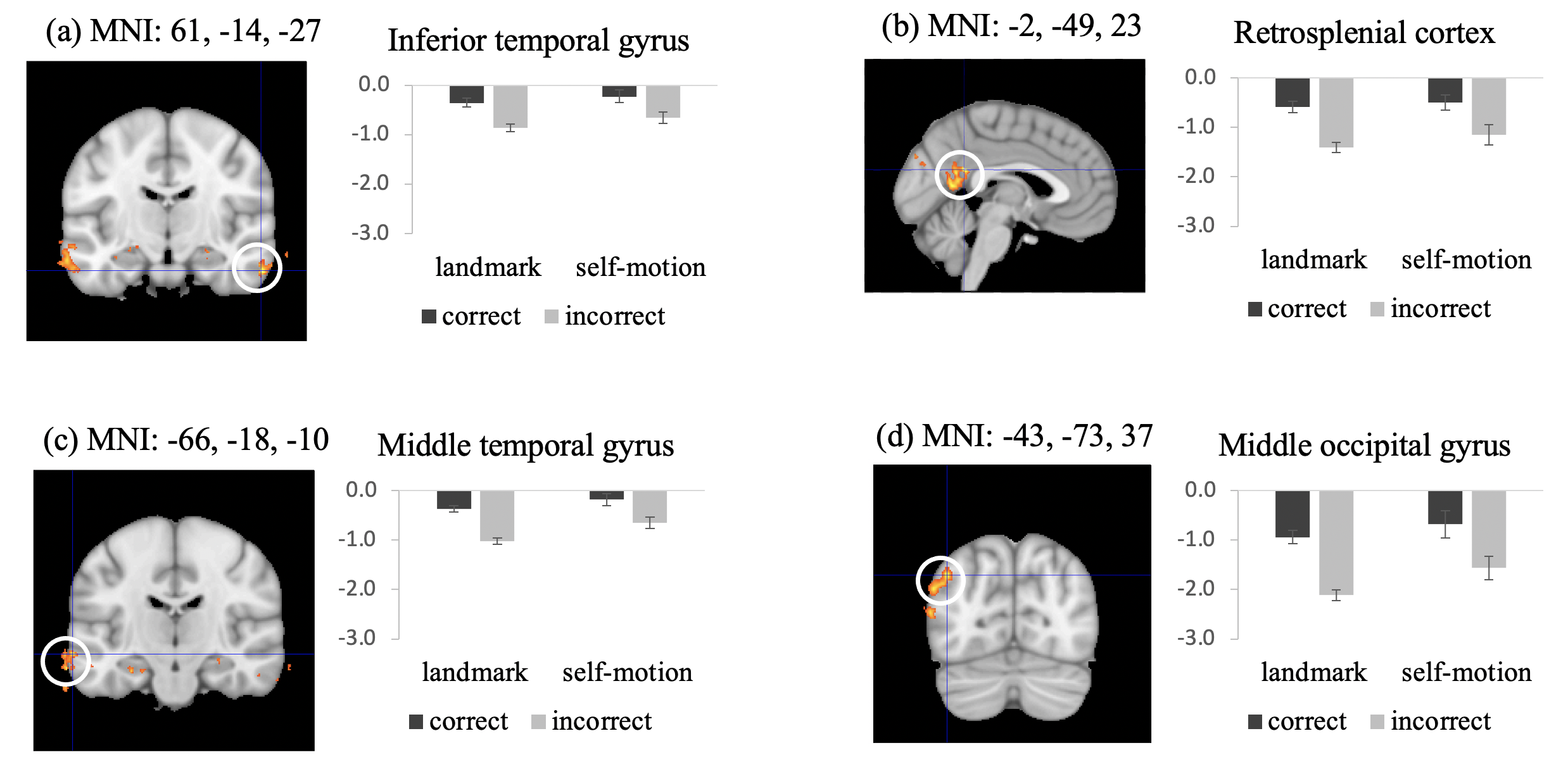


**Figure E9. Results of voxel-wise analysis of successful navigation.**

Four significant clusters showing significantly greater activation during correct trials than incorrect trials. The voxel-wise analysis was performed using the non-parametric permutation-based test. The cluster-level inference approach was adopted to correct for multiple comparisons across the entire search volume, with a cluster-defining threshold T > 3. The results were displayed on the MNI template, thresholded at T > 3. To visualize the successful navigation effect, mean brain activation was extracted for each significant cluster, which was then plotted as a function of cue type and trial correctness.

**Table E2. Voxel-wise analysis of successful navigation with cluster inference.**

Multiple comparisons were corrected either within the group-level anatomical mask consisting of all ROIs, or across the entire search volume, using nonparametric voxel-inference (Nichols & Holmes, 2002). Cluster size was determined at voxel-wise T > 3. In correspondence to Figure S9.

| Brain Region | MNI | | Voxel Level (T) | Cluster Size (k) | P_2-tailed_ (cluster-level) |
| --- | --- | --- | --- | --- | --- |
|  | RH | LH |  |  |  |
| *MTL+RSC mask for small-volume-correction* | | | | | |
| Retrosplenial cortex |  | -2, -49, 23 | 7.99 | 1442 | < 0.001 |
| Lingual gyrus |  | -13, -47, 2 | 6.25 |  |  |
| Calcarine |  | -6, -51, 5 | 6.19 |  |  |
| *Multiple comparisons correction across the entire search volume* | | | | | |
| Inferior temporal gyrus | 61, -14, -27 |  | 8.18 | 801 | 0.050 |
| Middle temporal gyrus | 62, -5, -17 |  | 5.59 |  |  |
| Inferior temporal gyrus | 58, -8, -33 |  | 4.45 |  |  |
| Retrosplenial cortex |  | -2, -49, 23 | 7.99 | 2550 | 0.004 |
| Calcarine |  | -18, -49, 6 | 6.87 |  |  |
| Calcarine |  | -6, -51, 5 | 6.19 |  |  |
| Middle temporal gyrus |  | -66, -18, -10 | 5.86 | 2344 | 0.004 |
| Middle temporal gyrus |  | -60, -2, -22 | 5.42 |  |  |
| Middle occipital gyrus |  | -43, -73, 37 | 5.79 | 6303 | < 0.001 |
| Middle temporal gyrus |  | -52, -62, 4 | 5.64 |  |  |
| Middle temporal gyrus |  | -56, -64, 11 | 5.61 |  |  |

**Table E3. ROI-based analysis of navigational success, in** **comparison with Chen et al. (2019).**

Significant results are highlighted in bold. Since there were slightly more participants in Chen et al. (2019) than in the current study (20 vs. 22 participants), the effect size (i.e., η_p_^2^) is more comparable between the two studies. Results with ROI-specific statistical outliers winsorized are in parentheses.

| Brain Region | Current Study | | | Chen et al., 2019 | | |
| --- | --- | --- | --- | --- | --- | --- |
|  | F | p | η_p_^2^ | F | p | η_p_^2^ |
| Entorhinal cortex (right) | <0.001  (0.474) | 0.982  (0.500) | <0.001  (0.024) | **9.344**  **(9.01)** | **0.006**  **(0.007)** | **0.308**  **(0.311)** |
| Entorhinal cortex (left) | **3.829**  **(4.765)** | **0.065**  **(0.042)** | **0.168**  **(0.201)** | **6.250** | **0.021** | **0.229** |
| Retrosplenial cortex | **17.267**  **(16.047)** | **0.001**  **(< 0.001)** | **0.476**  **(0.458)** | **20.028**  **(20.196)** | **<0.001**  **(<0.001)** | **0.488**  **(0.502)** |
| Hippocampus | 1.375  (1.958) | 0.255  (0.178) | 0.068  (0.093) | **11.886** | **0.002** | **0.361** |
| Parahippocampal cortex | **6.282** | **0.021** | **0.248** | **12.309**  **(11.449)** | **0.002**  **(0.003)** | **0.370**  **(0.364)** |
| Perirhinal cortex | 0.823  (0.682) | 0.376  (0.419) | 0.775  (0.035) | **15.304** | **< .001** | **0.422** |

*2.9. Temporal signal-to-noise ratio*

Temporal signal-to-noise ratio (tSNR) was calculated for each voxel, which was then averaged across all voxels in the brain region. The results are depicted in Figure S11. To preview, we found that overall tSNR was significantly higher on the 2^nd^ scanning day than the 1^st^ scanning day, and that ROIs differed in tSNR.

First, we submitted tSNR to a repeated-measures ANOVA test, with brain region (= 5; the four EC subregions were grouped together), day, and run as independent variables. The main effect of brain region was significant (F(4,76) = 65.432, p < 0.001, η_p_^2^ = 0.775), meaning that the regions differed in tSNR. Post-hoc comparisons with Bonferroni-Holm multiple comparisons correction showed that PRC had higher tSNR than RSC, hippocampus, and PHC (ps_corrected_ < 0.001), which in turn had higher tSNR than EC (ps_corrected_ < 0.001). The main effect of day was significant (F(1,19) = 16.422, p < 0.001, η_p_^2^ = 0.464), and there were no significant interaction effects involving day, meaning that for all regions, tSNR significantly improved on the 2^nd^ than the 1^st^ scanning day. The interaction between region and run was significant (F(28,532) = 2.634, p < 0.001). Following-up analyses showed that for RSC and PHC, the main effect of run was significant (ps < 0.02), meaning that tSNR decreased linearly across runs (the linear trend of run was significant, RSC, t=4.905, p < 0.001; PHC, t = 4.383, p < 0.001), whereas the main effect of run was non-significant for other regions (ps > 0.07).

We also looked more closely at EC by dividing it to four subregions, which were submitted to a repeated-measure ANOVA test, with hemisphere (left vs. right) and entorhinal subregion (alEC vs. pmEC) as independent variables. The main effect of hemisphere was significant (F(1,19) = 39.179, p < 0.001, η_p_^2^ = 0.673), meaning that the left EC had higher tSNR than the right EC. The main effect of subregion was not significant (F(1,19) = 1.503, p = 0.235, η_p_^2^ = 0.073). The interaction between hemisphere and subregion was significant (F(1,19) = 4.560, p = 0.046, η_p_^2^ = 0.194), in that alEC showed greater hemispheric specificity than pmEC.

**Figure E10. Temporal signal-to-noise ratio (tSNR).**

‘d1’ – 1^st^ scanning day; ‘d2’ – 2^nd^ scanning day; ‘r1’ to ‘r8’ – run 1 to run 8. Error bars represent ±SE.

We tested whether the general increase in tSNR on the 2^nd^ scanning compared to the 1^st^ scanning day was related to potentially differential magnitudes of head motion on the two days. we quantified the magnitude of head motion by calculating framewise displacement, using the SPMUP toolbox (https://github.com/CPernet/spmup). As shown in Figure S12a, the repeated-measure ANOVA test with day and run as independent variables showed that the main effect of day was not significant (F(1,19) = 0.542, p = 0.471, $\eta_{p}^{2}$= 0.028), indicating no difference in head motion magnitude between the two days. The main effect of run was significant (F(7, 133) = 8.750, p < 0.001, $\eta_{p}^{2}$= 0.315), meaning that as the time went by within the same day, participants moved their heads to a larger extent. Similar results were obtained when we analyzed the number of outlier scans defined by framewise displacement (Figure S12b): main effect of day, F(1,19) = 0.101, p = 0.754, $\eta_{p}^{2}$= 0.005; main effect of run, F(7,133) = 5.176, p < 0.001, $\eta_{p}^{2}$= 0.214.

The results showed that head motion magnitude could not explain the increase of tSNR on the 2^nd^ scanning day. We speculated that there should be other factors at play. The most possible factor is habituation and familiarity. The novelty and anxiety associated with the first day of scanning can lead to heightened stress or tension. On the second day, participants might be more relaxed and familiar with the process. Habituation and familiarity might also have helped participants developed more effective strategies of controlling head motion on the 2^nd^ scanning day, meaning that they spent less efforts on maintaining the same magnitude of head motion on the 2^nd^ day of scanning compared to the 1^st^ scanning day.


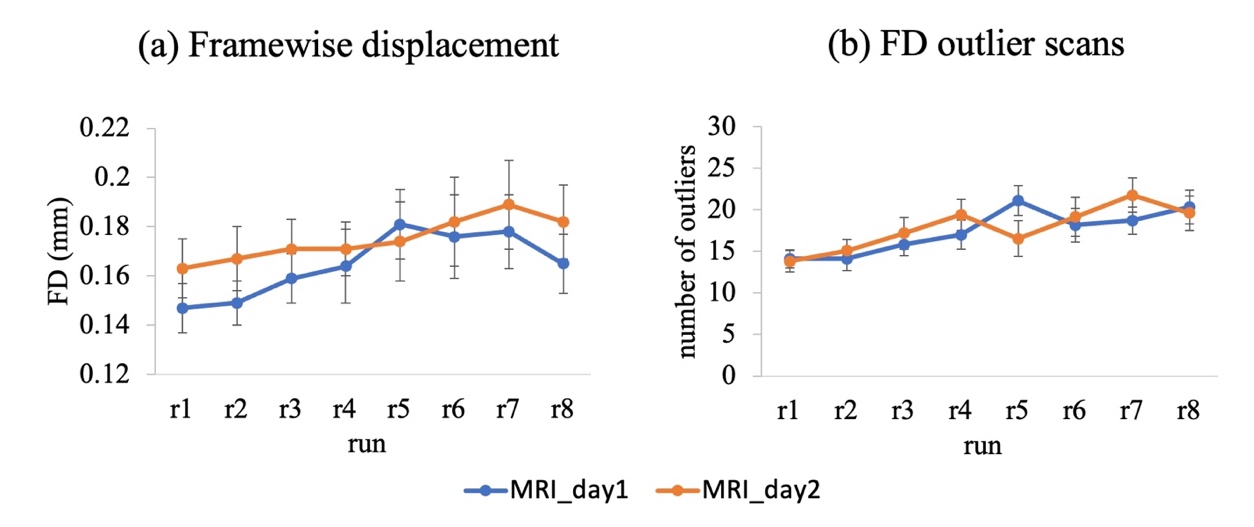


**Figure E11. Head motion.**

1. The mean framewise displacement was calculated for each run in each scanning day, using the SPMUP toolbox.
2. The number of scans identified as outliers based on framewise displacement (FD), using the SPMUP toolbox.

Error bars represent ±SE.

*2.10. Adaptation pattern analysis in the hippocampus*


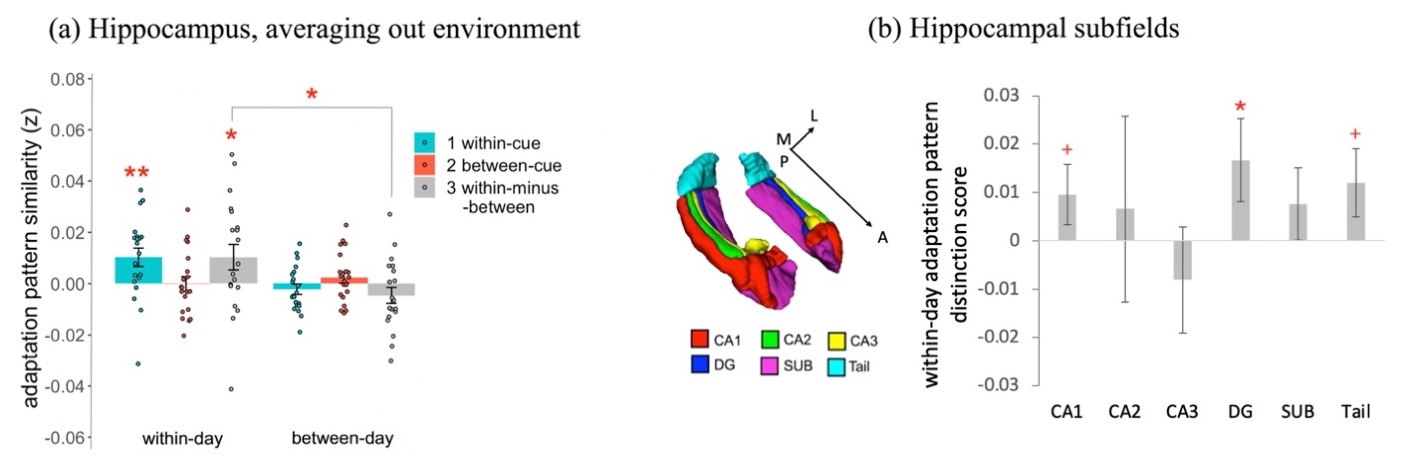


**Figure E12. Adaptation pattern analysis in the hippocampus.**

1. Adaptation pattern similarity is plotted as a function of day (within-day vs. between-day) and cue relation (within-cue vs. between-cue vs. within-minus-between-cue). Adaptation vectors were averaged across the two environments prior to the calculation of adaptation pattern similarity. In correspondence to Figure 8b.
2. Results of the hippocampal subfields. The left panel shows the manual segmentation of an exemplar participant’s hippocampus. The right panel shows the within-day adaptation pattern distinction score for each subfield (= within-cue minus between-cue adaptation pattern similarity). The score reached statistical significance for DG (p_1-tailed_ = 0.034, uncorrected).

‘*’ represents p_1-tailed/2-tailed_ < 0.05; ‘+’ represents p_1-tailed_ < 0.1; ‘DG’ – dentate gyrus; ‘SUB’ – subiculum.

*2.11. Strong functional connectivity between RSC and the hippocampus*


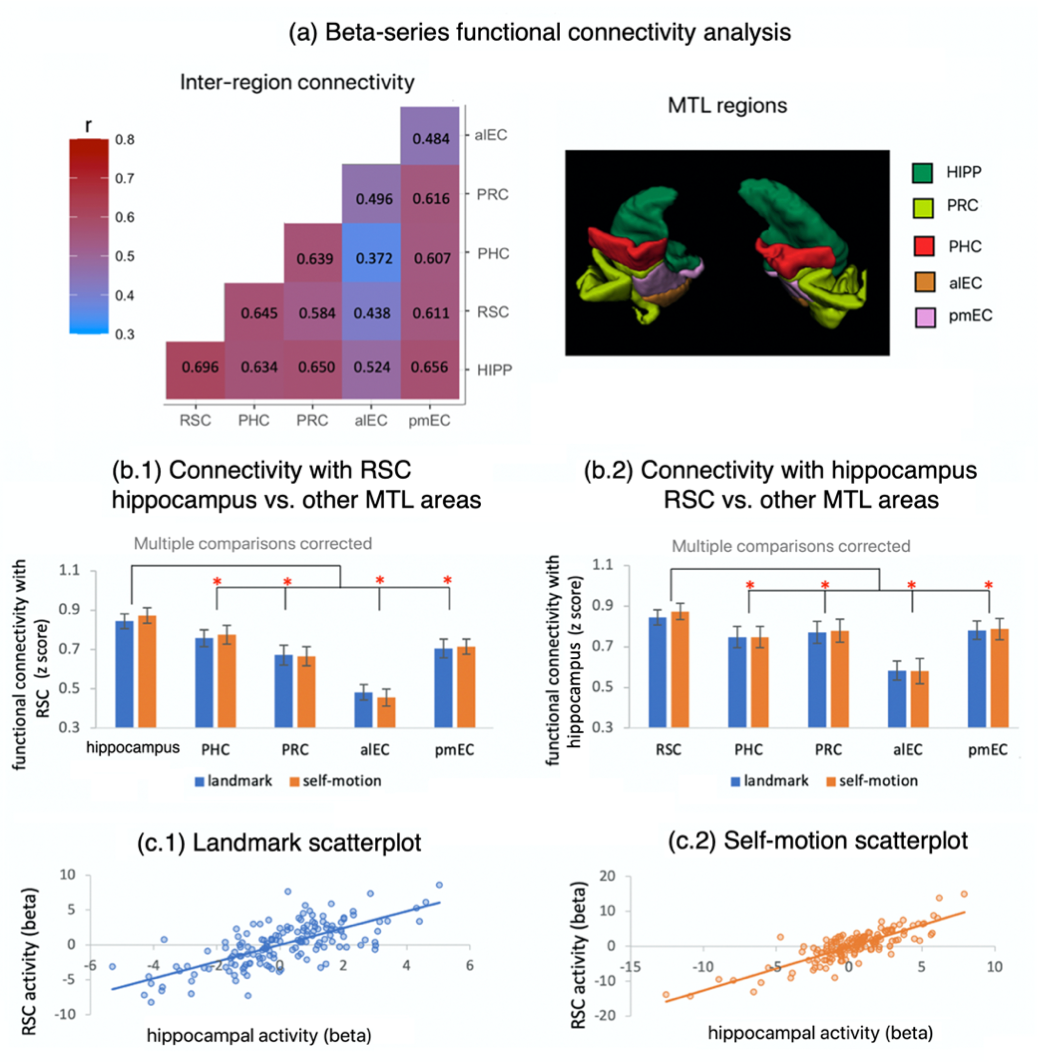


**Figure E13. Functional connectivity between retrosplenial cortex and hippocampus.**

1. Results of the beta-series functional connectivity analysis. Displayed on the left is the mean pairwise simple correlations among RSC and the medial temporal lobe (MTL) regions. Displayed on the right are anatomical masks of MTL regions for an exemplary participant. We assessed the functional connectivity between these regions using the beta-series connectivity analysis (Cisler et al., 2014), using MVPS-GLM-single-cue that modeled individual trials with separate regressors. For each brain region, we obtained a temporal sequence of activation estimates concatenated across individual trials, which were mean-centered within each run prior to the trial concatenation. We then calculated pairwise Pearson r correlations (fisher-transformed) between the temporal sequences of these regions for each participant. There existed strong functional coupling between RSC and hippocampus in both the landmark condition (p2-tailed < 0.001, BF10 > 1000) and the self-motion condition (p2-tailed < 0.001, BF10 > 1000).
2. (b.1) RSC-hippocampus connectivity was compared to RSC’s connectivity with other ROIs. The five pairs differed significantly in connectivity (F(4,76) = 36.079, p < 0.001, $\eta_{p}^{2}$= 0.655). Planned comparisons showed that RSC-hippocampus connectivity was significantly stronger than RSC’s connectivity with other MTL regions. (b.2) RSC-hippocampus connectivity was compared to the hippocampus’s connectivity with other ROIs. The five pairs differed significantly in connectivity (F(4,76) = 15.549, p < 0.001, $\eta_{p}^{2}$= 0.450). Planned comparisons showed that RSC-hippocampus connectivity was significantly stronger than the hippocampus’s connectivity with other MTL regions. Effects involving cue type were not significant. * denotes pholm,2-tailed < 0.05.
3. Scatterplot of trial-by-trial activation of the hippocampus and RSC in the landmark condition (c.1) and the self-motion condition (c.2) in an exemplary participant.

RSC: retrosplenial cortex; HIPP: hippocampus; PHC: parahippocampal cortex; PRC: perirhinal cortex; alEC: anterior-lateral entorhinal cortex; pmEC: posterior-medial entorhinal cortex.

References

Aguirre, G. K., Mattar, M. G., & Magis-Weinberg, L. (2011). De Bruijn cycles for neural decoding. *NeuroImage*, *56*(3), 1293–1300.

Gower, J. C., & Dijksterhuis, G. B. (2004). Procrustes problems. In *Oxford Statistical Science Series*. Oxford University Press.

Haak, K. V., Marquand, A. F., & Beckmann, C. F. (2018). Connectopic mapping with resting-state fMRI. *NeuroImage*, *170*, 83–94.

Hooke, R., & Jeeves, T. A. (1961). “Direct search” solution of numerical and statistical problems. *Journal of Association of Computing Machinery*, *8*, 212–229.

Kruskal, J., & Wish, M. (1978). *Multidimensional Scaling*. SAGE Publications, Inc.

Marchette, S. A., Vass, L. K., Ryan, J., & Epstein, R. A. (2014). Anchoring the neural compass: Coding of local spatial reference frames in human medial parietal lobe. *Nature Neuroscience*, *17*(11), 1598–1606.

Peer, M., & Epstein, R. A. (2021). The human brain uses spatial schemas to represent segmented environments. *Current Biology*, *31*(21), 4677-4688.e8.

Persichetti, A. S., & Dilks, D. D. (2019). Distinct representations of spatial and categorical relationships across human scene-selective cortex. *Proceedings of the National Academy of Sciences*, *116*(42), 21312–21317.

Zhang, W., & Luck, S. J. (2008). Discrete fixed-resolution representations in visual working memory. *Nature*, *453*(7192), 233–235.
